# Effectiveness of a scalable, remotely delivered stepped-care intervention for psychological distress among Polish migrant workers in the Netherlands: The RESPOND randomised controlled trial

**DOI:** 10.1371/journal.pmed.1005197

**Published:** 2026-08-17

**Authors:** Rinske Roos, Anke B. Witteveen, José Luis Ayuso-Mateos, Corrado Barbui, Richard A. Bryant, Zlata Dontsova, Mireia Felez-Nobrega, Zofia Głowacka, Josep Maria Haro, Raffael Kalisch, Vincent Lorant, David McDaid, Roberto Mediavilla, Maria Melchior, Pablo Nicaise, Emilia Olechno, A-La Park, Papoula Petri-Romão, Marianna Purgato, Aleksandra M. Sobolewska, Annemieke van Straten, Andrea Tortelli, James Underhill, Marit Sijbrandij, on behalf of the RESPOND Consortium

**Affiliations:** 1 Department of Clinical, Neuro- and Developmental Psychology, WHO Collaborating Center for Research and Dissemination of Psychological Interventions, Vrije Universiteit Amsterdam, Amsterdam, The Netherlands; 2 Amsterdam Public Health Research Institute, Amsterdam, The Netherlands; 3 Department of Psychiatry, Universidad Autónoma de Madrid, Madrid, Spain; 4 Centro de Investigación Biomédica en Red de Salud Mental (CIBERSAM), Instituto de Salud Carlos III, Madrid, Spain; 5 WHO Collaborating Centre for Research and Training in Mental Health and Service Evaluation, Department of Neurosciences, Biomedicine and Movement Sciences, Section of Psychiatry, University of Verona, Verona, Italy; 6 School of Psychology, University of New South Wales, Sydney, New South Wales, Australia; 7 Black Dog Institute, Sydney, New South Wales, Australia; 8 Parc Sanitari Sant Joan de Déu, Institut de Recerca Sant Joan de Déu (IRSJD), Sant Boi de Llobregat, Barcelona, Spain; 9 Centro de Investigación Biomédica en Red en Salud Mental (CIBERSAM), Madrid, Spain; 10 Leibniz Institute for Resilience Research, Mainz, Germany; 11 Neuroimaging Center (NIC), Focus Program Translational Neuroscience (FTN), Johannes Gutenberg University Medical Center, Mainz, Germany; 12 Institute of Health and Society (IRSS), UCLouvain, Brussels, Belgium; 13 Care Policy and Evaluation Centre, Department of Health Policy, The London School of Economics and Political Science, London, United Kingdom; 14 Sorbonne Université, INSERM, Institut Pierre Louis d'Épidémiologie et de Santé Publique, IPLESP, Épidémiologie Sociale, Santé Mentale et Addictions, Paris, France; 15 GHU Psychiatrie Neurosciences, Paris, France; 16 Independent Research Consultant, Brighton, United Kingdom; University of Saskatchewan, CANADA

## Abstract

**Background:**

Many international migrant workers (IMWs) in the Netherlands experience symptoms of anxiety and depression, often linked to adverse work and living conditions, yet underutilise mental healthcare. Stepped-care approaches may improve access by offering low-intensity support, followed by more intensive treatment if needed. We evaluated the effectiveness of a culturally adapted, remotely delivered, scalable stepped-care intervention combining guided online self-help (Doing What Matters in Times of Stress; DWM; step 1) and individual psychological support via videoconferencing (Problem Management Plus; PM+; step 2) both delivered by non-professional helpers, among Polish migrant workers living in the Netherlands.

**Methods and findings:**

In this parallel-group, two-arm, superiority randomised controlled trial, conducted in the Netherlands, Polish-speaking migrant workers aged 18 years or older with elevated psychological distress (Kessler Psychological Distress Scale; K10 ≥ 16) were recruited between May 2022 and January 2024 via social media and Polish community spaces. All participants received Psychological First Aid (PFA) before being informed about their allocation. Participants were randomly assigned 1:1 to care-as-usual or stepped-care (DWM, followed by PM+ if distress persisted; K10 ≥ 16) using computer-generated randomisation with permuted blocks in Castor Electronic Data Capture (Castor EDC). Participants and the main researcher were not blinded after allocation; outcomes were self-reported, and research assistants handling later incidental follow-up contact remained blinded. Assessments occurred at four timepoints: baseline (week 1), post-DWM (week 7), post-PM+ (week 13), and 2-month follow-up (week 21). The primary outcome was a composite measure of anxiety and depression (Patient Health Questionnaire-Anxiety and Depression Scale; PHQ-ADS), measured at all timepoints, with the primary endpoint at follow-up. Primary analyses followed the intention-to-treat (ITT) principle using linear mixed models adjusted for baseline PHQ-ADS values. In total, 218 participants were randomised and included in the ITT analysis (109 in each group); PHQ-ADS data at the primary endpoint were available for 94 participants in the intervention group and 101 in the control group. At follow-up, adjusted mean PHQ-ADS scores were lower in the intervention group than in the control group (14.8 versus 21.3), with a between-group mean difference adjusted for baseline PHQ-ADS score of −6.52 (95% confidence interval [CI] [−8.73, −4.31]; *p* < 0.001; Cohen’s *d* = −0.57), reflecting a moderate effect. Significant between-group differences adjusted for baseline PHQ-ADS were also observed post-DWM (−5.90; 95% CI [−8.12, −3.69]; *p* < 0.001; *d* = −0.56) and post-PM+ (−4.87; 95% CI [−7.18, −2.56]; *p* < 0.001; *d* = −0.44). Most participants in the intervention group who completed the post-DWM assessment still met criteria for PM+ (85/95, 89.5%). Eight serious adverse events occurred (intervention: *n* = 2; control: *n* = 6), and none were considered related to the intervention. The main limitations were limited generalisability, as most participants were women and had lived in the Netherlands for several years, and limited conclusions about longer-term effects due to the short follow-up period.

**Conclusion:**

The DWM/PM+ stepped-care intervention significantly reduced symptoms of anxiety and depression among Polish migrant workers in the Netherlands. These findings support the use of a remotely delivered stepped-care model combining scalable psychological interventions for migrant workers with elevated psychological distress.

## Author summary

**Why was this study done?:**

- Many international migrant workers in the Netherlands experience symptoms of anxiety and depression but often face barriers to accessing mental healthcare.
- Scalable psychological support delivered remotely may help reach people who might otherwise not receive help, but evidence for such approaches among international migrant workers is limited.
- We tested whether a remotely delivered stepped-care programme, combining guided online self-help *(Doing What Matters in Times of Stress; DWM)* with brief individual psychological support via video calls *(Problem Management Plus; PM+)* when needed, could reduce symptoms of anxiety and depression among Polish migrant workers.

**What did the researchers do and find?:**

- We conducted a randomised controlled trial including 218 Polish migrant workers living in the Netherlands who reported psychological distress, who were randomly assigned to receive either care-as-usual or a remotely delivered stepped-care psychological intervention (DWM/PM+).
- Participants receiving the intervention showed greater reductions in symptoms of anxiety and depression over time than those receiving care-as-usual.
- Many participants improved after the online self-help step (DWM), while additional individual support (PM+) helped reduce symptoms further among those who continued to experience higher levels of distress.

**What do these findings mean?:**

- Remotely delivered stepped-care psychological support may help reduce symptoms of anxiety and depression among international migrant workers.
- Such programmes may help expand access to mental health support for people who may have difficulty accessing traditional services or who are waiting for specialist care.
- Because most participants were women and had already lived in the Netherlands for several years, and because the study only followed participants for a short time, more research is needed to know whether this programme also works for other migrant populations and over longer periods.

## Abstract 3

In a randomised controlled trial of a remotely-delivered intervention, Rinske Roos and colleagues report that guided online self-help followed by psychological support by video-conferencing reduced symptoms of anxiety and depression in migrant workers in the Netherlands.

## Introduction

In 2022, 167.7 million international migrant workers (IMWs) comprised 4.7% of the global labour force [1]. IMWs are foreign-born individuals aged ≥15 years who were part of the labour force of their country of residence [2]. Nearly a quarter of IMWs live in Northern, Southern, and Western Europe, comprising 17.4% of the labour force. In the Netherlands, nearly 1 million employees (13.0%) are foreign-born, over half from the European Union (EU), primarily Poland [3,4]. IMWs in the Netherlands are typically EU citizens employed in temporary, low-paid, physically demanding jobs in sectors such as agriculture, logistics, and industry [5,6].

IMWs often live in overcrowded or substandard housing, experience high stress levels, and face barriers to healthcare [6–9]. A meta-analysis found that anxiety and depression prevalence among IMWs is 39% and 27%, respectively [10]. Risk factors include poor living conditions, occupational stressors (e.g., physically demanding work, job insecurity), barriers to healthcare, and limited social support [10]. Despite this burden and ongoing marginalisation, IMWs make limited use of mental health services due to language barriers, limited awareness of available care, stigma, and inflexible work schedules [6,11]. The COVID-19 pandemic exacerbated these vulnerabilities, exposing IMWs to high infection risk, job loss, housing insecurity, and mobility disruption [6,12,13].

The World Health Organization (WHO) has developed several scalable, transdiagnostic psychological interventions to reduce psychological distress in populations affected by adversity, using a task-shifting approach where trained non-specialists, so-called ‘helpers’, deliver care. Two such interventions are Doing What Matters in Times of Stress (DWM) [14] and Problem Management Plus (PM+) [15]. DWM is an illustrated stress management guide based on acceptance and commitment therapy (ACT), originally developed to complement Self-Help Plus (SH+), a group-based stress management course [16]. It was adapted into a guided self-help intervention delivered over five weekly modules via a web application, with optional weekly guidance from a trained helper to support engagement and practice of the strategies [16]. PM+ is a brief intervention rooted in cognitive behavioural therapy (CBT), typically delivered in five individual sessions by a trained helper [15]. PM+ has shown effectiveness in reducing symptoms of depression, anxiety, and post-traumatic stress disorder (PTSD) among people experiencing psychological distress [17], while SH+ has been found to prevent the onset of mental disorders in populations with elevated distress [18].

Stepped-care expands access to care while conserving resources: individuals start with a low-intensity, evidence-based treatment (lower burden in time and cost for individuals and providers). Progress is monitored; only those not (significantly) responding step up to higher-intensity treatment [19]. Guided self-help interventions can have similar effects to face-to-face therapy, supporting its use as the first step [20]. Digital interventions such as DWM are flexible and remove travel barriers. Given the high prevalence of anxiety, depression, and related psychological distress among IMWs and their limited access to specialist services, stepped-care offers a pragmatic strategy to match treatment intensity to need. Accordingly, we selected guided DWM as step 1, and PM+ as step 2 for those with persistent distress, a sequence with promising results among migrants in Italy [21], and healthcare workers in Spain [22].

Before evaluating the effectiveness of this stepped-care programme, we conducted a formative qualitative study to explore IMWs’ problems, daily functioning, and help-seeking behaviour in the Netherlands and to inform the cultural adaptation of DWM/PM+ [6]. Most migrant worker participants in that study were Polish, and many professionals primarily worked with Polish migrant workers. Participants described multiple, interrelated stressors, including precarious employment, housing precarity, administrative barriers, and difficulties accessing (mental) healthcare. Based on these findings, the prominent position of Polish migrant workers in the Netherlands, and practical considerations regarding translation of materials and language-specific intervention delivery, we therefore decided to focus the present study on Polish-speaking migrant workers.

### Objective

This study aimed to evaluate the effectiveness of the DWM/PM+ stepped-care intervention, compared with care-as-usual, in reducing symptoms of anxiety and depression among Polish migrant workers living in the Netherlands.

## Methods

### Study design and participants

This parallel-group, two-arm, superiority randomised controlled trial (RCT) was conducted in the Netherlands by Vrije Universiteit Amsterdam. It was registered in the Dutch Trial Register (NL9630; https://www.onderzoekmetmensen.nl/en/trial/27052, 08/08/2021). Full details are available in the published protocol [23]. No substantial changes were made, except for a change in the method of obtaining written informed consent, which switched from printed consent returned by mail to digital consent mid-recruitment, and the method of imputation (see Statistical analysis section). Results are reported in accordance with Consolidated Standards of Reporting Trials (CONSORT) 2025 guidelines (S1 Checklist) [24].

Eligible participants were Polish migrant workers (≥ 18 years) living in the Netherlands, with elevated psychological distress (Kessler Psychological Distress Scale; K10 ≥ 16) [25,26], sufficient Polish language skills, and internet access. As only Polish-speaking individuals were enrolled, all procedures and materials in this study were provided exclusively in Polish. Exclusion criteria included: intention to move abroad within six months, acute medical conditions, imminent suicide risk, severe mental disorder (e.g., psychotic disorder), cognitive impairment (e.g., severe intellectual disability), current specialist psychological treatment (e.g., CBT), or recent psychotropic medication changes (past two months).

### Randomisation and masking

Participants were randomly assigned (1:1) to the intervention or control group via Castor Electronic Data Capture (Castor EDC) [27]. The random allocation sequence was computer-generated within Castor EDC using permuted blocks (sizes 4 and 6), with no stratification. After participant inclusion, the main researcher manually initiated randomisation in Castor EDC, after which group allocation was revealed to this researcher. Before being informed about their randomisation group, all participants received Psychological First Aid (PFA) (see Procedures section). The research assistant delivering PFA checked the participant’s allocation only after completing the PFA-support conversation, and then disclosed it to the participant. This was done by a research assistant not involved in later participant follow-up contact. Research assistants handling incidental participant follow-up contact (e.g., safety procedures, participant queries) remained blinded.

### Procedures

Recruitment targeted Polish migrant workers primarily via social media and Polish community spaces (e.g., supermarkets, beauty salons, non-governmental organisations) across the Netherlands. All materials and procedures were in Polish; participant contact was handled by Polish-speaking research assistants. Interested individuals had an initial telephone call with the research assistants, during which the study was explained and they could ask preliminary questions. If they remained interested, they received the participant information sheet and informed consent form, either digitally or on paper, and a second call was scheduled at least one week later to answer any remaining questions. During this second call, they could ask further questions and indicate whether they wanted to participate. Those who decided to participate then provided formal written informed consent, either digitally or on paper. After informed consent had been obtained, participants completed a screening assessment (*t*_0_): a self-administered online questionnaire via Castor EDC [27] and a brief video interview.

Subsequent assessments (*t*_1_–*t*_4_) were self-administered via Castor, and included sociodemographic, clinical, and contextual measures (e.g., COVID-19 impact, stressors, service use). In both groups, follow-up assessments were intended to fall at approximately week 7, 13, and 21. However, the operational triggers for *t*_2_ and *t*_3_ differed slightly between arms to ensure that, in the intervention group, these assessments closely followed completion of each treatment step. In the control group, *t*_2_ was triggered 42 days (6 weeks) after completion of the baseline assessment (*t_1_*, week 1), *t*_3_ 42 days after completion of *t*_2_, and *t*_4_ 140 days (20 weeks) after completion of baseline (*t_1_*). In the intervention group, *t*_2_ was sent 6 weeks after participants received access to DWM, and *t*_3_ was sent 1 week after completion of the last PM+ session. The follow-up assessment *t*_4_ was again scheduled 140 days (20 weeks) after completion of baseline, as in the control group. Thus, although the intended timing of *t*_2_ and *t*_3_ assessments was comparable across arms, in the stepped-care arm assessments were aligned with completion of the intervention steps (DWM and PM+) resulting in some real-world variability around the nominal weeks 7 and 13. Accordingly, *t*_2_ represents the immediate post-DWM assessment in the stepped-care arm (corresponding approximately to week 7 assessment in the control arm), *t*_3_ represents the immediate post-PM+ assessment in the stepped-care arm (corresponding to week 13 assessment in the control arm), and *t*_4_ represents the two-month follow-up assessment in both groups. Assessments remained open for 14 days, with reminders on day 2, 5, and 10. If < 14 days remained before *t*_4_, *t*_3_ was skipped. Participants received €10 (gift voucher) per completed assessment.

Three days after baseline, all participants (i.e., both intervention and control group), received PFA. PFA is a brief support strategy for psychological distress, providing humane, practical help. Key elements include identifying and addressing basic needs, providing support, and promoting safety [28]. This ensured all participants received some initial support. PFA was delivered by phone and the call lasted ±15 min. The intervention group then received DWM, and PM+ if distress persisted at *t*_2_ (K10 ≥ 16). All participants had unrestricted access to monitored care-as-usual [23]. Both the PFA session and the subsequent stepped-care intervention (DWM/PM+) were fully remote and in Polish.

DWM is a five-week guided self-help programme delivered via a mobile-friendly web application. A new module unlocks weekly, regardless of module completion, offering audio exercises and stress management techniques. Modules cover ACT-based strategies (e.g., present-moment awareness, cognitive defusion, acceptance of unpleasant feelings, values clarification, committed action, self-compassion) [16]. Participants received a 15-minute introductory call and optional weekly guidance (phone or asynchronous messages, per participant preference).

PM+ is a five-session, weekly individual intervention (60 min) focused on practical strategies (e.g., problem-solving, behavioural activation) [15]. Printed hand-outs were sent by post and additionally provided by email as PDF attachments, to support engagement with videoconference-based sessions. Missed sessions were followed up by helpers to reschedule.

PFA was delivered by psychology students (research assistants), and DWM and PM+ by volunteer native Polish speakers in the Netherlands (i.e., non-specialist helpers). They received a hybrid Training of Helpers using WHO protocols: 2 days for DWM and 7–8 days for PM+, including role plays and script practice. Training was delivered by WHO-trained professionals. Three training rounds were held (2022–2023). Of 16 helpers who started training, 14 (all female) completed and worked as helpers (10 DWM/PM+, 4 (psychology students) DWM only); 2 male helpers discontinued during/after training. Clinical psychologists provided weekly online supervision, with WHO-trainer support as needed. Participants were paired with a helper based on availability, and thus could have different helpers for DWM and PM+.

Both DWM and PM+ were culturally adapted for Polish migrant workers, informed by formative qualitative research, guided by Bernal and colleagues’ framework [29]. Key adaptations included remote delivery, shortened PM+ sessions (90 → 60 min), and culturally tailored content (see [6]).

### Outcomes

The K10 (10 items, 1–5 Likert, range 10–50; strong psychometric properties [25]) was used to assess non-specific psychological distress at *t*_0_ (screening) and was repeated at *t*_2_ (post-DWM) to determine eligibility for step-up to PM+ . At *t*_0_, suicide risk (suicidal thoughts interview from the PM+ manual) and cognitive impairment (PM+ observation checklist) were also assessed.

The primary outcome was symptoms of anxiety and depression at follow-up (*t*_4_), measured with the 16-item Patient Health Questionnaire-Anxiety and Depression Scale (PHQ-ADS), a composite of the Patient Health Questionnaire-9 (PHQ-9, 0–27) [30] and Generalized Anxiety Disorder-7 (GAD-7, 0–21) [31] (0–3 Likert scale; total range 0–48). The primary endpoint was the two-month follow-up assessment (*t*_4_). Treatment effects on PHQ-ADS were prespecified to be estimated at all post-baseline assessments (*t*_2_, *t*_3_, and *t*_4_). The validated Polish PHQ-9 was used [32]. The Polish GAD-7 is not yet formally validated, but has shown high internal consistency in several COVID-19 studies [33–35].

Secondary outcomes included symptoms of depression (PHQ-9) [30], anxiety (GAD-7) [31], and post-traumatic stress (PTSD Checklist for DSM-5, PCL-5, 8-item version; range 0–32) [36] at *t*_2_, *t*_3,_ and *t*_4_. Clinical cut-offs were ≥10 for probable anxiety and depression [31,37], and ≥ 19 for probable PTSD [36]. The Polish 20-item PCL-5, with good psychometric properties [38], served as the basis for the 8-item version used in this study [36]. Across all scales, higher scores indicated greater symptom severity. Baseline Cronbach’s alpha values were 0.92 (PHQ-ADS), 0.83 (PHQ-9), 0.89 (GAD-7), and 0.87 (PCL-5).

At baseline (*t_1_*) and follow-up (*t*_4_), participants completed a 10-item version of the Brief Trauma Questionnaire (BTQ) [39], translated into Polish by the study team per WHO guidelines [40]. At *t_1_*–*t*_4_, participants completed additional prespecified measures, including a COVID-19 impact questionnaire. The BTQ and COVID-19 impact items are included in this manuscript as contextual variables and/or exploratory moderators, as described in the Statistical analysis section.

Four additional prespecified measures were collected at *t*_1_–*t*_4_ but are not reported in the present manuscript because they address separate research questions related to resilience-related mechanisms and cost-effectiveness. These measures will be reported in separate planned manuscripts. For the resilience-related analysis, positive appraisal style was assessed using the Positive Appraisal Style Scale - content focused (PASS-content) [41], and stressor exposure was assessed using a questionnaire adapted from the Mainz Inventory of Microstressors (MIMIS) [42] and similar measures [43]. The measures collected for the cost-effectiveness analysis are health-related quality of life assessed using the EuroQol 5-dimensional descriptive system—5-level version (EQ-5D-5L) [44], and service use assessed using the Client Service Receipt Inventory (CSRI) [45].

Safety monitoring included a suicide screening question at *t*_2_–*t*_4_. If positive, the suicidality module of the Mini-International Neuropsychiatric Interview (MINI) [46] was administered by telephone; scores ≥ 17 (high-risk) were considered serious adverse events and reported to the Medical Ethics Committee. Participants were monitored until stabilised and referred to their general practitioner if needed. No Data Safety Monitoring Board was involved.

Only the PHQ-9, GAD-7, and suicide screener were mandatory. No outcome changes were made after trial initiation. Treatment fidelity (i.e., helpers’ adherence to intervention manuals) was monitored throughout. With participant consent, calls/sessions were recorded; every 10th recording was reviewed using a fidelity checklist, stratified by helper. Most were reviewed by one rater; a subset by two independent raters.

### Statistical analysis

Sample size calculations (power = 0.95, *α* = 0.05, two-sided) were based on detecting a small-to-medium effect group × time interaction effect of Cohen’s *f* = 0.30 on the primary outcome (PHQ-ADS at *t*_4_), informed by prior PM+ trials [47,48]. Using G*Power, we modelled the group × time (baseline versus *t*_4_) interaction with an *F* test in a 2 × 2 repeated-measures analysis of variance (ANOVA), as a standard approximation of the planned mixed-effects analysis. The effect size was specified as Cohen’s *f* = 0.30 for the within-between interaction, using G*Power specification in which the correlation among repeated measurements is treated implicitly. This gave a required sample of 148 participants with complete data (74 per group). Allowing 30% attrition, the target sample size was set at 212 participants (106 per group). The study was powered for the primary outcome only; secondary and exploratory analyses were not powered and should be interpreted cautiously.

Missing scale items were handled for the PHQ-9, GAD-7, and PCL-5. The PHQ-9 and GAD-7 together constitute the PHQ-ADS primary outcome; therefore, item-level imputation on these scales directly informed the PHQ-ADS total score. Imputation was performed if ≤ 50% were missing. When a single item was missing, person mean substitution (proration) was applied, whereby the missing item was replaced by the mean of the participant’s completed items, rounding to the nearest whole number (PHQ-9: *n* = 1; GAD-7: *n* = 0; PCL-5: *n* = 13). This approach is consistent with scoring recommendations for instruments such as the PHQ-9, where prorating is permitted when item-level missingness is low (e.g., < 20%–25%) [49]. For multiple missing items (≤ 50%), multiple imputation by chained equations (MICE) [50] with predictive mean matching and five imputations was used (PCL-5: *n* = 14). If > 50% of items within a scale were missing, the scale score at that specific assessment was treated as missing rather than imputed, to avoid generating scores based predominantly on imputed values. When participants did not complete a questionnaire at a given follow-up assessment, the outcome at that time point was recorded as missing. Sum scores were calculated only when all items were available following imputation. BTQ scores reflected the number of endorsed items (range 0–10).

Baseline characteristics were summarised descriptively by randomisation group; no formal statistical tests of between-group baseline differences were performed, as such tests are not recommended in randomised trials [51]. To examine whether realised assessment timing differed between arms, the observed number of days between completed assessments (*t*_1_–*t*_2_, *t*_2_–*t*_3_, *t*_3_–*t*_4_, and *t*_1_–*t*_4_) was calculated using Castor completion dates. Mean intervals were compared between randomisation groups using Welch two-sample t-tests.

Primary analysis followed an intention-to-treat (ITT) approach, including all randomised participants. PHQ-ADS was the primary outcome, with *t*_4_ as the primary endpoint. The primary estimate was the between-group difference in PHQ-ADS scores at *t*_4_, estimated using linear mixed models (LMMs) adjusting for baseline PHQ-ADS values. This approach estimates the treatment effect at *t*_4_ while adjusting for baseline PHQ-ADS values and is statistically equivalent to analysing change from baseline under standard assumptions. Secondary outcomes (PHQ-9, GAD-7, and PCL-5) were analysed using the same modelling framework. Intervention completers were defined as those completing ≥3 DWM modules and, if eligible for PM+, attending ≥4 sessions. Per-protocol (PP) analyses were conducted as sensitivity analyses including intervention completers only; control participants were analysed as randomised.

LMMs included fixed effects for time (categorical; *t*_2_–*t*_4_), randomisation group, the respective baseline score as covariate, and time × group interaction, with a random intercept for participant to account for within-person correlation. The random intercept accounts for within-person correlation across repeated assessments, whereas inclusion of the baseline score as a covariate adjusts for initial severity. This specification allows estimation of treatment effects at follow-up while adjusting for baseline severity. All randomised participants were included in the primary outcome model. LMMs were estimated using maximum likelihood, which incorporates all available outcome data under a missing-at-random assumption and allows inclusion of all randomised participants without requiring separate imputation of missing follow-up scores.

Two models were estimated: a baseline-adjusted model as the primary analysis, and a covariate-adjusted model as a sensitivity analysis, additionally including gender, age, education level, relationship status, work hours per week (based on work sector), trauma history (any versus none), and COVID-19 infection. Covariates were derived from baseline questionnaire data and, where required, recoded or grouped for analysis. They were selected a priori based on clinical relevance to assess the robustness of the primary findings. These variables were treated as covariates in the sensitivity analyses, whereas potential moderators were examined separately in prespecified moderation analyses. Estimated marginal means (EMMs) were calculated per group and time point, averaged across levels for categorical covariates and estimated at the sample mean for continuous covariates. At each time point, between-group differences with 95% confidence intervals (CIs), *p*-values and effect sizes (Cohen’s *d*) were calculated. Effect sizes were based on LMM-estimated EMMs (adjusted for baseline and covariates) and the pooled standard deviation (*SD*) from raw (unadjusted) data at that time point. All tests were two-tailed. P-values are reported alongside effect sizes and 95% confidence intervals to allow interpretation beyond statistical significance alone.

PHQ-ADS moderation analyses at *t*_4_ (ITT) used the same modelling framework, both unadjusted and adjusted for baseline scores. Potential effect modification by baseline characteristics was examined in separate moderator analyses by including interaction terms between randomisation group, time, and the respective potential moderator. Moderators included baseline PHQ-ADS, gender, age, relationship status, education, duration of stay in the Netherlands, current work sector, prior mental health service consultation, number of traumatic events (BTQ), and negative psychological impact of COVID-19. Where needed, moderator variables were derived from the original questionnaire responses by collapsing categories, recoding open-text responses, or creating grouped variables for analysis. When evidence of effect modification was observed, subgroup analyses were conducted to support interpretation of the interaction by estimating intervention effects separately within levels of the moderator.

Reliable change and clinical recovery (ITT) on PHQ-ADS were calculated as exploratory outcomes using the Reliable Change Index (RCI), based on baseline Cronbach’s alpha and *SD* [52]. Recovery was defined as reliable improvement (RCI < −1.96) and a score below the clinical cut-off (2 *SD*s below baseline mean).

Further exploratory analyses examined factors influencing intervention uptake or outcomes, including subgroup analyses by baseline distress (K10: 16–29 (low-to-moderate) versus 30–50 (severe) [53]), and within-group comparisons (DWM completers versus non-completers, PM+ eligible versus non-eligible, PM+ completers versus non-completers). In these analyses, we compared groups on sociodemographic characteristics, symptoms of anxiety and depression (PHQ-ADS), PHQ-ADS change scores, and explored whether helper continuity was associated with PM+ completion and *t*_4_ PHQ-ADS scores.

All statistical analyses were conducted in R (version 4.4.2), using RStudio (version 2024.12.1). All analyses followed the published protocol [23], unless indicated as exploratory (not prespecified). No interim analyses or stopping guidelines were planned.

## Results

### Participants

**Fig 1:**
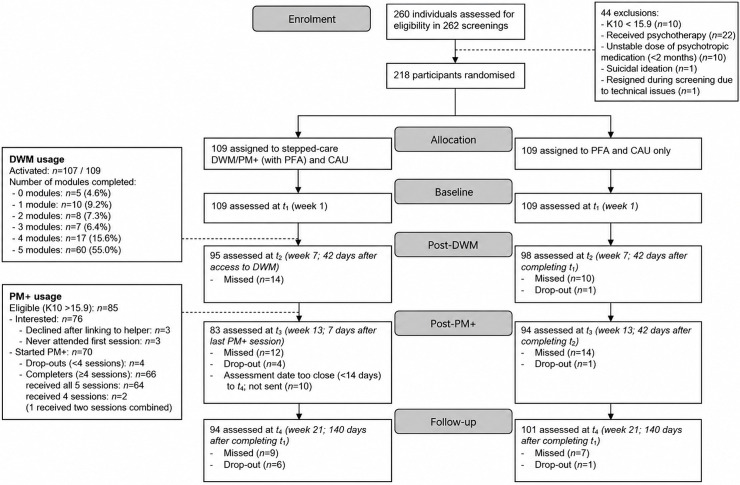
CONSORT flow diagram. *Note.* All participants assessed were included in the intention-to-treat primary outcome analysis. "Drop-out” includes participants who actively withdrew from the study; “Missed” includes participants who missed the assessments but remained enrolled. Abbreviations: CAU, care-as-usual; PFA, Psychological First Aid; DWM, Doing What Matters in Times of Stress; PM+ = Problem Management Plus; K10 = Kessler Psychological Distress Scale.

Between May 2022 and January 2024, 1,190 individuals contacted the research team. Many could not be reached or declined after receiving study information. Of 481 who received a consent form, 280/481 (58.2%) provided consent. Twenty did not complete screening (e.g., lost contact). Of 260 screened, 218 were randomised (109 per group; Fig 1). The first participant was enrolled on June 3, 2022, and the last participant on January 25, 2024. Recruitment ended once the planned minimum target sample size of 212 participants had been reached. The final sample slightly exceeded this target because several individuals had already provided informed consent and had screening appointments scheduled and were therefore still screened and enrolled if eligible. Follow-up assessments were completed up to June 19, 2024.

Most participants identified as female (*n* = 162, 74.7%) with a mean age of 38.8 years. Most lived permanently in the Netherlands (*n* = 204, 94.0%; mean 6.1 years), though some alternated residence between countries. Nearly half worked in physical/industrial sectors (*n* = 92, 47.2%), and 105 participants (64.0%) had regular daytime schedules. Mean baseline PHQ-ADS score was 24.7 (*SD* = 9.8), indicating moderate symptoms of anxiety and depression. K10 screening scores were high (*M* = 30.01, *SD* = 7.53, range: 16–48), with no group difference (*p* = 0.427). Most participants (*n* = 131, 64.9%) had previously consulted mental health services. Cut-offs for probable anxiety, depression, and PTSD were met by 130 (59.6%), 159 (72.9%), and 119 (55.1%), respectively; 159 (72.9%) reported a traumatic experience. See Table 1 for baseline characteristics; S1 Table for the full overview.

**Table 1:** Baseline sociodemographic, clinical, and COVID-related characteristics of participants by randomisation group.

Variables	Randomisation group		
	Intervention *n* = 109	Control *n* = 109	Full sample *N* = 218
Gender, *n (%)*			
Female	86 (78.9)	76 (70.4)	162 (74.7)
Male	23 (21.1)	30 (27.8)	53 (24.4)
Other	0	2 (1.9)	2 (0.9)
Age, *M (SD)* (range)	37.5 (9.1) (20–61)	40.0 (9.5) (20–61)	38.8 (9.4) (20–61)
Relationship status, n (%)*			
In a steady relationship/married	73 (67.0)	67 (61.5)	140 (64.2)
Not in a relationship	36 (33.0)	42 (38.5)	78 (35.8)
Children, n (%)*			
Yes	48 (44.0)	48 (44.4)	96 (44.2)
No	61 (56.0)	60 (55.6)	121 (55.8)
Educational level, n (%)*			
Primary or secondary education	44 (43.6)	45 (44.6)	89 (44.1)
Professional education or university	57 (56.4)	56 (55.4)	113 (55.9)
Country of residence pattern, n (%)*			
Permanent residence in the Netherlands	102 (93.6)	102 (94.4)	204 (94.0)
Alternating residence in the Netherlands and another country	7 (6.4)	6 (5.6)	13 (6.0)
Duration of stay in the Netherlands (in months), *M (SD)* (range)	69.1 (57.7) (3–240)	77.7 (56.3) (3–216)	72.8 (57.0) (3–240)
Speaks Dutch and/or English, *n (%)*			
Yes	92 (85.2)	84 (84.0)	176 (84.6)
No	16 (14.8)	16 (16.0)	32 (15.4)
Income, *n (%)*			
Yes	91 (91.9)	91 (91.9)	182 (91.9)
No	8 (8.1)	8 (8.1)	16 (8.1)
Current work sector, n (%)*			
Administrative and support sectors	7 (7.3)	7 (7.1)	14 (7.2)
Commercial sectors	13 (13.5)	17 (17.2)	30 (15.4)
Education, health care, and uniformed professions	14 (14.6)	16 (16.2)	30 (15.4)
Physical and industrial sectors	47 (49.0)	45 (45.5)	92 (47.2)
Other	15 (15.6)	14 (14.1)	29 (14.9)
Hours work/week, *M (SD)* (range)†	35.3 (10.7) (2–70)	35.9 (9.1) (0–52)	35.6 (9.9) (0–70)
Work schedule, *n (%)*†			
Regular daytime hours	54 (66.7)	51 (61.4)	105 (64.0)
Shift work (changing hours)	27 (33.3)	32 (38.6)	59 (36.0)
Ever consulted a mental health service, *n (%)*			
Yes	66 (65.3)	65 (64.4)	131 (64.9)
No	35 (34.7)	36 (35.6)	71 (35.1)
Anxiety/depression symptoms (PHQ-ADS score, 0–48), *M (SD)* (range)	23.89 (9.16) (8–48)	25.49 (10.44) (5–46)	24.69 (9.83) (5–48)
Anxiety symptoms (GAD-7 score, 0–21), *M (SD)* (range)	10.72 (4.85) (3–21)	11.44 (5.44) (1–21)	11.08 (5.15) (1–21)
Probable anxiety disorder (GAD-7 ≥10), *n (%)*	63 (57.8)	67 (61.5)	130 (59.6)
Depression symptoms (PHQ-9 score, 0–27), *M (SD)* (range)	13.17 (5.09) (5–27)	14.05 (5.65) (4–26)	13.61 (5.38) (4–27)
Probable depressive disorder (PHQ-9 ≥10), *n (%)*	79 (72.5)	80 (73.4)	159 (72.9)
Post-traumatic stress symptoms (PCL-5 score, 0–32), *M (SD)* (range)	17.54 (6.88) (3–31)	19.23 (6.79) (2–32)	18.38 (6.87) (2–32)
Probable PTSD (PCL-5 ≥19), *n (%)*	56 (51.4)	63 (58.9)	119 (55.1)
Traumatic experiences (brief BTQ score, 0–10), *M (SD)* (range)	1.82 (1.88) (0–8)	2.39 (2.48) (0–10)	2.10 (2.21) (0–10)
At least 1 traumatic experience (BTQ), *n (%)*	79 (72.5)	80 (73.4)	159 (72.9)
Negative psychological impact of COVID-19, n (%)*			
Yes	57 (54.3)	62 (62.6)	119 (58.3)
No or don’t know	48 (45.7)	37 (37.4)	85 (41.7)

*Note.* Missing values per variable and randomisation group (*i* = intervention group, *c* = control group): Gender (*c*: 1 [0.9%]), Age (*i*: 2 [1.8%]; *c*: 1 [0.9%]), Speaks Dutch and/or English (*i*: 1 [0.9%]; *c*: 9 [8.3%]), Duration of stay in the Netherlands (*i*: 45 [41.3%]; *c*: 61 [56.0%]), Ever consulted a mental health service (*i*: 8 [7.3%]; *c*: 8 [7.3%]), Educational level (*i*: 8 [7.3%]; *c*: 8 [7.3%]), Country of residence (*c*: 1 [0.9%]), Current work sector (*i*: 13 [11.9%]; *c*: 10 [9.2%]), Hours work/week (*c*: 1 [0.9%]), Work schedule (*c*: 3 [2.8%]), Income (*i*: 10 [9.2%]; *c*: 10 [9.2%]), Children (*c*: 1 [0.9%]), Post-traumatic stress symptoms (PCL-5)/Probable PTSD (PCL-5 ≥ 19) (*c*: 2 [1.8%]), COVID-19 psychological impact (*i*: 4 [3.7%]; *c*: 10 [9.2%]).

* Response categories were combined where applicable. S1 Table shows the full baseline categories and, for ‘Current work sector’, the grouping of detailed categories into the broader sectors presented here.

† Only presented to participants who reported current employment (81 intervention (74.3%), 86 control (78.9%)). Abbreviations: *M*, mean; *SD*, standard deviation; PHQ-ADS, Patient Health Questionnaire-Anxiety and Depression Scale; PHQ-9, Patient Health Questionnaire-9; GAD-7, Generalized Anxiety Disorder-7; PCL-5, PTSD Checklist for DSM-5; BTQ, Brief Trauma Questionnaire.

PHQ-ADS (primary outcome) data were available for all 218 participants (100.0%) at *t*_1_ (baseline), 193 (88.5%) at *t*_2_ (post-DWM), 177 (81.2%) at *t*_3_ (post-PM+), and 195 (89.4%) at *t*_4_ (follow-up), with no group differences in attrition. Across outcome measures, completion ranged from 216/218 to 218/218 (99.1%–100%) at *t*_1_, 192/218 to 193/218 (88.1%–88.5%) at *t*_*2*_, 170/218 to 178/218 (78.0%–81.7%) at *t*_3_, and 193/218 to 195/218 (88.5%–89.4%) at *t*_4_. PCL-5 (non-mandatory) had the most missing data. In the intervention group, drop-outs (*n* = 6) were less likely to speak Dutch/English (*p* = 0.004) and tended to have lived in the Netherlands longer (*p* = 0.021) than non-drop-outs (*n* = 103).

Twenty-six participants (intervention: *n* = 9; control: *n* = 17) required a total of 36 suicide assessments. Of these, 28 assessments were conducted, while eight assessments involving seven participants were not conducted due to non-response or refusal. Eight of the 28 conducted assessments (28.6%) were considered a serious adverse event (intervention: *n* = 2; control: *n* = 6). No adverse events were considered related to the intervention.

The realised assessment timing differed between randomisation groups at *t*_2_, *t*_3_, and *t*_4_. The mean interval from *t*_1_–*t*_2_ was 45.5 days in the control group versus 57.1 days in the intervention group, and from *t*_2_–*t*_3_ 45.0 versus 53.3 days (both *p* < 0.001). The interval from *t*_3_–*t*_4_ was 53.3 days in the control group and 34.0 days in the intervention group (*p* < 0.001). The overall interval from *t*_1_–*t*_4_ was similar across groups (143.7 versus 145.3 days; *p* = 0.041).

### Intervention uptake

In the intervention group (*n* = 109), 107/109 participants (98.2%) activated the DWM app, and 84/109 (77.1%) completed ≥ 3 modules (Fig 1). These DWM completers finished on average 4.63 modules (*SD* = 0.64), against 1.13 (*SD* = 0.76) for non-completers (*n* = 25). Completion rates declined across modules, from 94/109 (86.2%) for module 1 to 64/109 (58.7%) for module 5.

Of 95 intervention participants completing *t*_2_, 85/95 (89.5%) qualified for PM+ (K10 ≥ 16). Of these, 76/85 (89.4%) were linked to a helper (27 to the same as DWM) and 70/85 (82.4%) started PM+. Reasons for not starting included travel, workload, starting therapy, or finding DWM sufficed. Of the 85 participants eligible for PM+, 66/85 (77.6%) completed ≥ 4 PM+ sessions (Fig 1).

On average, participants received access to DWM 8 days after being informed of group allocation (range: 1–24; median: 7), and started PM+ 14 days after being informed of eligibility (range: 2–46; median: 11). Of the 64 who completed all PM+ sessions, 48 (75.0%) did so within 5–6 weeks (*M* = 5.6, range: 4–9), with some delays (e.g., no internet credit, driving lessons in Poland). T4 follow-up occurred on average 36.9 days (*SD* = 11.5, range: 9–58) after the last PM+ session. Helpers supported on average 7.3 DWM participants and 6.0 PM+ completers (range: 2–12 for both).

### Treatment fidelity

In total, 103/109 participants (94.5%) consented to DWM call recordings, resulting in 339 recordings; 55/70 (78.6%) of those who started PM+ consented to PM+ session recordings, resulting in 208 recordings. Fidelity was assessed for 33 DWM support calls and 21 PM+ sessions. On average, 91.2% of DWM and 82.0% of PM+ protocol components were delivered. Inter-rater agreement was 96.0% (DWM) and 100% (PM+; 15 and 8 double-rated, respectively).

### Primary outcome

**Fig 2:**
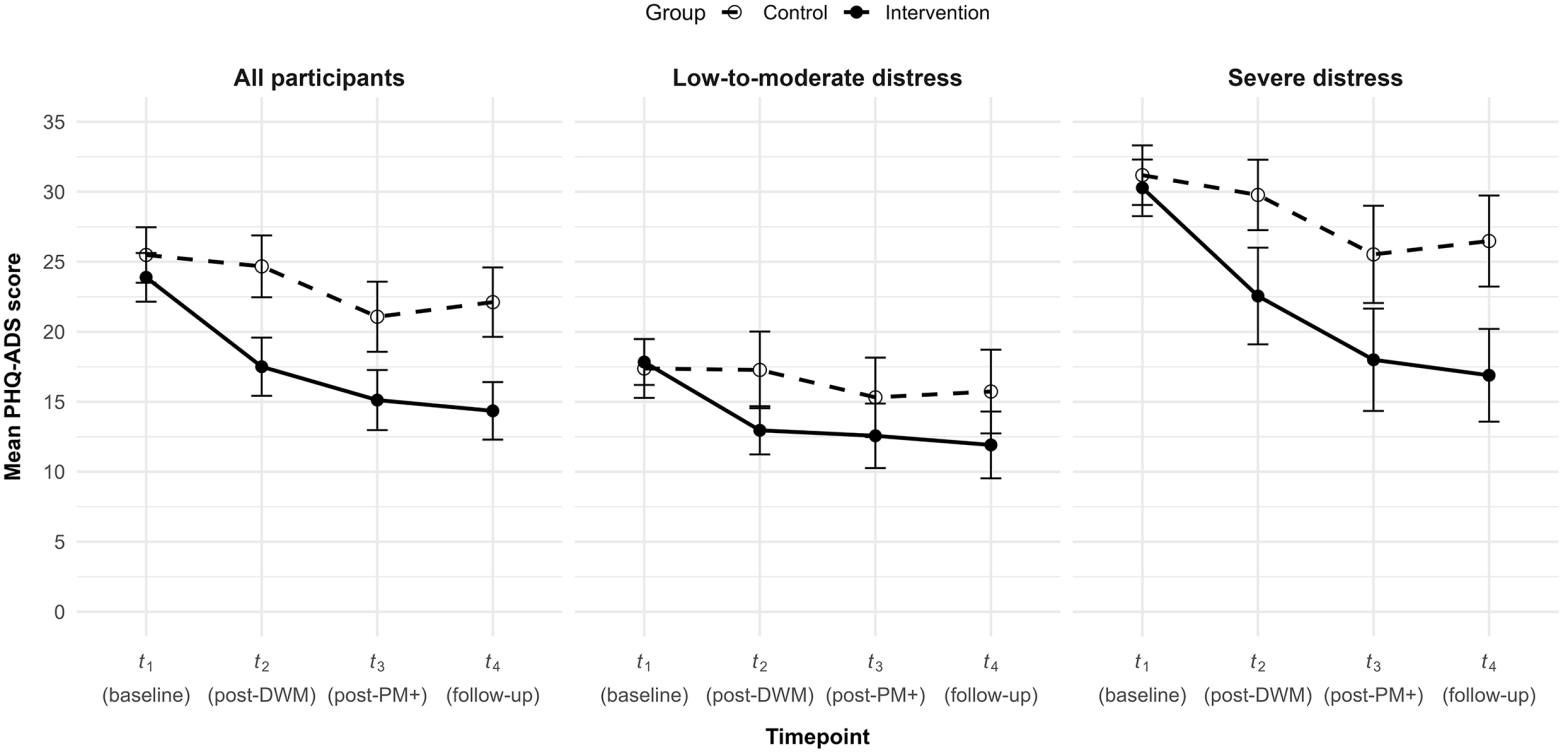
Mean PHQ-ADS scores over time per randomisation group (intention-to-treat) with 95% confidence intervals, for all participants and by baseline psychological distress severity (K10; low-to-moderate: K10 16–29, n = 101; severe: K10 30–50, n = 117). Intervention group = solid line; control group = dashed line. *Note.* PHQ-ADS, Patient Health Questionnaire-Anxiety and Depression Scale; K10, Kessler Psychological Distress Scale; DWM, Doing What Matters in Times of Stress; PM+, Problem Management Plus.

At *t*_4_ (follow-up; primary endpoint), PHQ-ADS scores (symptoms of anxiety and depression) were significantly lower in the intervention group than in the control group, with a baseline-adjusted mean difference of −6.52 (95% confidence interval [CI] [−8.73, −4.31]; *p* < 0.001; Cohen’s *d* = −0.57), indicating a moderate effect. Significant between-group differences for the primary outcome were also found at *t*_2_ (post-DWM; −5.90; 95% CI [−8.12, −3.69]; *p* < 0.001; *d* = −0.56), and at *t*_3_ (post-PM+; −4.87; 95% CI [−7.18, −2.56]; *p* < 0.001; *d* = −0.44; see Fig 2 and Table 2).

**Table 2:** PHQ-ADS (primary outcome) summary statistics, estimated marginal means, and effect sizes per timepoint (intention-to-treat, baseline-adjusted linear mixed model).

		Intervention			Control					
K10 group	Timepoint	*N*	*M (SD)*	EMM (95% CI)	*N*	*M (SD)*	EMM (95% CI)	Difference (95% CI)	*p*-value	Cohen’s *d*
All participants (K10: 16–50)	*t*_1_ (baseline, week 1)	109	23.9 (9.2)		109	25.5 (10.4)				
	*t*_2_ (post-DWM, week 7)	95	17.5 (10.2)	18.3 (16.7, 19.8)	98	24.7 (11.0)	24.2 (22.6, 25.7)	−5.90 (−8.12, −3.69)	<0.001	−0.56
	*t*_3_ (post-PM+, week 13)	83	15.1 (9.8)	16.0 (14.3, 17.7)	94	21.1 (12.2)	20.9 (19.3, 22.4)	−4.87 (−7.18, −2.56)	<0.001	−0.44
	*t*_4_ (follow-up, week 21)	94	14.4 (10.0)	14.8 (13.2, 16.4)	101	22.1 (12.6)	21.3 (19.8, 22.8)	−6.52 (−8.73, −4.31)	<0.001	−0.57
Low-to-moderate distress (K10: 16–29)	*t*_1_ (baseline, week 1)	56	17.8 (6.1)		45	17.4 (7.0)				
	*t*_2_ (post-DWM, week 7)	50	13.0 (6.1)	13.0 (11.1, 14.9)	40	17.3 (8.6)	17.3 (15.2, 19.4)	−4.24 (−7.07, −1.40)	0.004	−0.58
	*t*_3_ (post-PM+, week 13)	44	12.6 (7.6)	12.7 (10.7, 14.7)	41	15.3 (9.0)	15.7 (13.6, 17.8)	−2.99 (−5.89, −0.09)	0.043	−0.36
	*t*_4_ (follow-up, week 21)	48	11.9 (8.2)	11.9 (10.0, 13.8)	41	15.7 (9.5)	15.7 (13.6, 17.8)	−3.83 (−6.67, −0.98)	0.009	−0.43
Severe distress (K10: 30–50)	*t*_1_ (baseline, week 1)	53	30.3 (7.3)		64	31.2 (8.5)				
	*t*_2_ (post-DWM, week 7)	45	22.6 (11.5)	22.8 (20.4, 25.3)	58	29.8 (9.6)	29.9 (27.7, 32.1)	−7.05 (−10.34, −3.76)	<0.001	−0.67
	*t*_3_ (post-PM+, week 13)	39	18.0 (11.3)	18.4 (15.8, 21.0)	53	25.5 (12.6)	25.3 (23.0, 27.6)	−6.89 (−10.36, −3.42)	<0.001	−0.57
	*t*_4_ (follow-up, week 21)	46	16.9 (11.2)	16.9 (14.5, 19.4)	60	26.5 (12.6)	26.1 (24.0, 28.2)	−9.17 (−12.42, −5.93)	<0.001	−0.77

*Note*. Observed descriptive values, presented as *N* and *M* (*SD*), are unadjusted. Model-based estimates, including EMMs, between-group differences, *p*-values, and Cohen’s *d*, are adjusted for the PHQ-ADS baseline outcome score.

Abbreviations: *M*, mean; *SD*, standard deviation; EMM, estimated marginal mean; CI, confidence interval; PHQ-ADS, Patient Health Questionnaire-Anxiety and Depression Scale; K10, Kessler Psychological Distress Scale; DWM, Doing What Matters in Times of Stress; PM+, Problem Management Plus.

### Secondary outcomes

Similar effects were observed across secondary outcomes. At *t*_4_, between-group differences were −3.31 for PHQ-9 (95% CI [−4.56, −2.06]; *p* < 0.001; *d* = −0.52), −3.25 for GAD-7 (95% CI [−4.40, −2.10]; *p* < 0.001; *d* = −0.58), and −1.91 for PCL-5 (95% CI [−3.52, −0.31]; *p* = 0.020; *d* = −0.23). Earlier timepoints showed similar patterns across outcomes, all favouring the intervention group. Effect sizes ranged from small (PCL-5) to moderate (PHQ-9 and GAD-7), depending on time point (see Table 3). Covariate-adjusted models showed results similar to the baseline-adjusted models, indicating that the main findings were robust to additional covariate adjustment, although PCL-5 at follow-up was no longer significant (see S2 Table). PP analyses (intervention *n =* 68; control *n =* 109) mirrored ITT results, with slightly larger effect sizes. All effects remained significant, except PCL-5 post-DWM (see S3 Table).

**Table 3:** Secondary outcome summary statistics, estimated marginal means, and effect sizes per timepoint (intention-to-treat, baseline-adjusted linear mixed model).

		Intervention			Control					
Outcome	Timepoint	*N*	*M (SD)*	EMM (95% CI)	*N*	*M (SD)*	EMM (95% CI)	Difference (95% CI)	*p*-value	Cohen’s *d*
PHQ-9	*t*_1_ (baseline, week 1)	109	13.2 (5.1)		109	14.0 (5.6)				
	*t*_2_ (post-DWM, week 7)	95	9.7 (5.5)	10.2 (9.3, 11.1)	98	13.8 (6.3)	13.6 (12.7, 14.5)	−3.42 (−4.67, −2.16)	< 0.001	−0.58
	*t*_3_ (post-PM + , week 13)	84	8.5 (5.6)	8.9 (7.9, 9.8)	94	11.7 (6.9)	11.6 (10.7, 12.5)	−2.73 (−4.03, −1.43)	< 0.001	−0.43
	*t*_4_ (follow-up, week 21)	94	8.1 (5.6)	8.4 (7.5, 9.3)	101	12.1 (7.0)	11.7 (10.8, 12.6)	−3.31 (−4.56, −2.06)	< 0.001	−0.52
GAD-7	*t*_1_ (baseline, week 1)	109	10.7 (4.9)		109	11.4 (5.4)				
	*t*_2_ (post-DWM, week 7)	95	7.8 (5.2)	8.1 (7.2, 8.9)	98	10.9 (5.4)	10.6 (9.8, 11.4)	−2.53 (−3.68, −1.37)	< 0.001	−0.48
	*t*_3_ (post-PM+, week 13)	83	6.8 (5.0)	7.2 (6.3, 8.0)	94	9.3 (6.1)	9.3 (8.4, 10.1)	−2.10 (−3.30, −0.90)	< 0.001	−0.38
	*t*_4_ (follow-up, week 21)	94	6.2 (4.9)	6.4 (5.5, 7.2)	101	10.0 (6.2)	9.6 (8.8, 10.4)	−3.25 (−4.40, −2.10)	< 0.001	−0.58
PCL-5	*t*_1_ (baseline, week 1)	109	17.5 (6.9)		107	19.2 (6.8)				
	*t*_2_ (post-DWM, week 7)	94	13.7 (7.7)	14.4 (13.2, 15.5)	98	17.5 (7.7)	16.7 (15.6, 17.8)	−2.35 (−3.96, −0.74)	0.004	−0.31
	*t*_3_ (post-PM+, week 13)	81	11.8 (7.1)	12.8 (11.6, 14.1)	89	16.3 (8.7)	16.0 (14.8, 17.1)	−3.13 (−4.83, −1.43)	< 0.001	−0.39
	*t*_4_ (follow-up, week 21)	94	10.9 (7.9)	11.4 (10.3, 12.6)	99	13.9 (8.9)	13.4 (12.2, 14.5)	−1.91 (−3.52, −0.31)	0.020	−0.23

*Note*. Observed descriptive values, presented as *N* and *M* (*SD*), are unadjusted. Model-based estimates, including EMMs, between-group differences, p-values, and Cohen’s *d*, are adjusted for the respective baseline outcome score.

Abbreviations: M, mean; *SD*, standard deviation; EMM, estimated marginal mean; CI, confidence interval; PHQ-9, Patient Health Questionnaire-9; GAD-7, Generalized Anxiety Disorder-7; PCL-5, PTSD Checklist for DSM-5; DWM, Doing What Matters in Times of Stress; PM+, Problem Management Plus.

At follow-up, PHQ-ADS moderation analysis suggested effect modification by negative psychological impact of COVID-19 (baseline-adjusted *p* = 0.007) and by total duration of stay in the Netherlands (baseline-adjusted *p* = 0.037). Intervention effects were stronger among participants reporting psychological impact of COVID-19 and among those with longer residence in the Netherlands. In subgroup analyses, the intervention effect at follow-up was larger among participants reporting psychological impact of COVID-19 (intervention *n* = 57, control *n* = 62; *d* = −0.70, *p* < 0.001) than among those reporting no negative impact or indicating that they did not know (intervention *n* = 48, control *n* = 37; *d* = −0.22, *p* = 0.193). Likewise, when duration of stay was dichotomised at the median of 60 months (5 years), the intervention effect at follow-up was larger among participants with longer residence (>60 months; intervention *n* = 26, control *n* = 22; *d* = −0.82, *p* < 0.001) than among those with shorter residence (≤60 months; intervention *n* = 38, control *n* = 26; *d* = −0.20, *p* = 0.295). No other moderation analyses showed meaningful effect modification.

### Exploratory analyses

Reliable change analysis (ITT) showed a clinical PHQ-ADS cut-off of 5.02. The proportion of participants with reliable improvement or recovery was higher in the intervention than control group across all timepoints (see Table 4).

**Table 4:** Reliable change outcomes (RCI) over timepoints for PHQ-ADS among participants with available data.

	*t*_2_ (post-DWM)		*t*_3_ (post-PM+)		*t*_4_ (follow-up)	
	Intervention (*n* = 95)	Control (*n* = 98)	Intervention (*n* = 83)	Control (*n* = 94)	Intervention (*n* = 94)	Control (*n* = 101)
Recovered, *n* (%)	3 (3.2%)	3 (3.1%)	10 (12.0%)	6 (6.4%)	12 (12.8%)	3 (3.0%)
Improved without recovery, *n* (%)	30 (31.6%)	11 (11.2%)	33 (39.8%)	24 (25.5%)	43 (45.7%)	27 (26.7%)
Deteriorated, *n* (%)	4 (4.2%)	11 (11.2%)	3 (3.6%)	9 (9.6%)	4 (4.3%)	12 (11.9%)
No change, *n* (%)	58 (61.1%)	73 (74.5%)	37 (44.6%)	55 (58.5%)	35 (37.2%)	59 (58.4%)

*Note*. Recovered = RCI <−1.96 and score ≤ clinical cut-off; improved without recovery = RCI <−1.96 and score > cut-off; deteriorated = RCI > 1.96; no change = −1.96 ≤ RCI ≤ 1.96.

Abbreviations: RCI = Reliable Change Index; PHQ-ADS = Patient Health Questionnaire-Anxiety and Depression Scale; DWM = Doing What Matters in Times of Stress; PM+ = Problem Management Plus.

A trend-level time-by-group-by-baseline psychological distress (K10) interaction (*p* = 0.062) suggested stronger effects among those with severe baseline distress. Subgroup analyses indicated larger between-group differences in PHQ-ADS scores in the severe compared to the low-to-moderate group, particularly at follow-up (*d* = −0.77 versus −0.43; see Fig 2 and Table 2).

Within the intervention group, DWM completers were more often female (*p* = 0.013) and higher educated (*p* < 0.001) than non-completers. They showed a trend toward lower post-DWM symptoms of anxiety and depression (PHQ-ADS: *p* = 0.053), but no baseline differences were observed in age, K10 psychological distress, PHQ-ADS scores, or change scores on either measure. Among participants eligible for stepping up to PM+ at *t*_2_, PHQ-ADS scores decreased from post-DWM to post-PM+ (*p* = 0.010) and to follow-up (*p* < 0.001), whereas no change was observed in the non-eligible group (S1 Fig). PM + non-completers reported lower PHQ-ADS scores at *t*_2_ than completers (*p* = 0.031), independent of baseline values or DWM use. Although PM+ completers more often had the same helper as in DWM compared with non-completers (25/66, 37.9% versus 2/10, 20.0%), helper continuity was not clearly associated with PM+ completion or follow-up PHQ-ADS scores.

## Discussion

A scalable, remotely delivered stepped-care intervention for Polish migrant workers in the Netherlands produced significantly greater reductions in symptoms of depression, anxiety, and PTSD compared to care-as-usual. Effects were robust across timepoints and covariate-adjusted models, with secondary outcomes showing similar patterns. These findings indicate that a brief, culturally adapted stepped-care model can improve mental health outcomes among IMWs facing high distress and barriers to care.

Reductions in symptoms of anxiety and depression mirrored RESPOND trials in Spain [22] and Italy [21]. The between-group effect at follow-up (*d* = −0.57), as well as at earlier post-intervention assessments, was larger than the small-to-medium effect size (*d* = −0.30) assumed in the sample size calculation, indicating that the intervention effect exceeded the magnitude anticipated at the design stage. Effect sizes were also consistent with a meta-analysis of PM+ and its digital version, Step-by-Step (SbS) [54], both after DWM and after the full stepped-care intervention. DWM yielded extensive symptom reduction, reflecting the effect of guided self-help in a population with elevated psychological distress (K10). However, most participants remained eligible for PM+, likely due to high baseline distress levels. PM+ led to additional gains, particularly among those with more severe symptoms. This aligns with a recent individual participant data meta-analysis (IPDMA) on PM+ and SbS, showing stronger effects for participants with higher baseline symptom severity [17]. Moderation analyses showed stronger effects among participants more negatively affected by COVID-19 restrictions. Possibly, this reflects the relevance of stress management, problem-solving, and psychological flexibility for pandemic-related distress. Stronger effects were also found among those with longer residence in the Netherlands, perhaps due to increased stability. However, unlike the IPDMA, education level did not moderate outcomes.

While DWM led to substantial symptom reductions, clinical recovery occurred mainly after PM+. Follow-up reliable recovery rates exceeded those reported by de Graaff and colleagues [55], despite comparable baseline common mental disorders. Although the average time between the final PM+ session and follow-up was considerably shorter than intended (5 weeks instead of 2 months), which warrants cautious interpretation, this pattern suggests added value of offering DWM before PM+, possibly through cumulative effects or improved readiness via the ACT-based strategies in DWM. Notably, PM+ uptake was high, countering concerns that stepped-care may discourage continuation to higher-intensity treatment following first-step non-response [19].

Helper characteristics may influence engagement [56], but as all helpers were women and gender did not moderate outcomes, no conclusions can be drawn regarding gender matching. Helper continuity across DWM and PM+ was also unrelated to adherence or outcomes. These findings should be interpreted cautiously, given limited variability and small numbers, and warrant further investigation in more diverse samples and settings.

This study demonstrates the effectiveness of a fully online, guided stepped-care intervention among Polish speakers in the Netherlands, a population experiencing high psychological distress and barriers to mental healthcare [6,8,9]. Baseline anxiety and depression rates were higher than previously reported for migrant workers [10], possibly reflecting that help-seeking in this group occurs mainly at more severe symptom levels, as seen in the cultural adaptation findings [6]. While this enhances ecological validity, it limits generalisability to less symptomatic populations and highlights the importance of offering accessible psychological support. Despite high symptom severity and the use of a web-based (instead of mobile) application, adherence was strong and attrition low. Completion rates exceeded those typically reported for digital interventions [57,58], underscoring the potential of structured guidance to enhance engagement, even with a limited digital platform.

Several limitations should be considered. First, external validity is limited by the sample characteristics and the absence of information on individuals who did not enrol. While recruitment targeted Polish migrant workers, inclusion was open to all Polish speakers; our sample likely included both IMWs and community members. Most participants were female, whereas most Polish labour migrants are male [59]. Many participants had been living in the Netherlands for several years, spoke Dutch and/or English, and were relatively well-educated, suggesting a more established group. These factors limit generalisability to newly arrived, less educated, or male migrant workers. In addition, we were unable to characterise individuals who received study information but did not provide consent or did not complete screening, as no sociodemographic or clinical data were collected prior to informed consent. Selection bias cannot therefore be ruled out, as individuals who consented, completed screening, and were randomised may have differed in important ways from those who received study information but did not enrol. This limits our ability to assess how representative the randomised sample was of the broader group reached during recruitment. Nevertheless, the high levels of psychological distress observed highlight the need for accessible mental health support.

Second, several limitations relate to the analysis and interpretation of secondary, exploratory, and sensitivity analyses. PP analyses were conducted as exploratory sensitivity analyses. Because these analyses exclude participants who did not finish an adequate number of DWM modules and, if eligible, receive an adequate number of sessions, they do not fully preserve the benefits of randomisation and may therefore be susceptible to bias [60]. Furthermore, the statistical analyses were conducted by an investigator who was not blinded to treatment allocation, rather than by an independent blinded statistician. Although the statistical analysis plan was prespecified and the primary analysis followed an intention-to-treat approach, lack of blinding during analysis may have introduced bias. Moreover, multiple statistical tests were conducted across outcomes, timepoints, adjusted models, and exploratory subgroup analyses. Although the primary endpoint was prespecified and powered for, the number of secondary and exploratory analyses increases the risk of Type I error. In addition, the study was not powered to detect subgroup or moderator effects, increasing the risk of Type II error and meaning that potentially relevant differences may have gone undetected. These findings should therefore be interpreted cautiously, particularly for secondary and subgroup analyses, and viewed as supportive of the primary ITT results.

Furthermore, the timing of the follow-up assessments was constrained by the practicalities of delivering a stepped-care intervention. In the intervention arm, the assessments *t*_2_ and *t*_3_ were anchored to treatment progression (post-DWM and post-PM+), whereas in the control arm they followed fixed calendar intervals. This resulted in differences in observed timing at these intermediate assessments. The primary endpoint (*t*_4_) was scheduled using the same 140-day (20-week) post-baseline rule in both arms, and the overall *t*_1_–*t*_4_ interval was highly similar between groups. Although the final assessment was intended to occur approximately two months after completion of PM+ in the intervention arm, the fixed 140-day schedule from baseline and real-world delays (e.g., time required to link participants to helpers, scheduling constraints, occasional session overruns) meant that the median interval between the last PM+ session and *t*_4_ was closer to five weeks. This limits conclusions about longer-term effectiveness. Future trials should account more explicitly for such real-world treatment-related delays when defining assessment windows and include longer follow-up periods to better assess durability of effects.

Beyond clinical outcomes, additional work is underway to examine appraisal style and the cost-effectiveness of this stepped-care approach, both essential for informing future implementation and funding decisions. Given substantial symptom improvement after DWM, future research should identify who benefits sufficiently from DWM alone, who requires the full stepped-care model, and who might benefit from PM+ directly. Finally, testing unguided or contact-on-demand DWM versions could determine whether guidance is essential to achieve comparable effects.

The substantial reduction in symptoms of anxiety and depression after DWM suggests that this brief, digital intervention is suitable as a first step, possibly also for those awaiting specialist mental healthcare. Offering DWM during waiting periods may reduce symptoms and enhance readiness for further treatment. After DWM, PM+ appears most beneficial for those with more severe baseline distress. Together, DWM and PM+ form a feasible, scalable stepped-care intervention for migrant workers facing significant psychological distress and barriers to accessing care. Policymakers and clinicians should consider integrating this approach into routine care, especially where language, cultural, or logistical barriers restrict mental healthcare access.

### Ethical approval

This study involves human participants and was approved by the Medical Ethics Committee of Amsterdam University Medical Centre, location Vrije Universiteit Medical Centre (protocol ID: 2021.0335, 31/08/2021). Participants gave written informed consent to participate in the study before taking part. The study was conducted in accordance with the ethical principles outlined in the Declaration of Helsinki.

## Supporting information

S1 Checklist CONSORT 2025 checklist. The CONSORT 2025 checklist was obtained from the SPIRIT–CONSORT website (https://www.consort-spirit.org/) and is licensed under the Creative Commons Attribution 4.0 International License (CC BY 4.0).
(DOCX)

S1 Fig PHQ-ADS trajectories by PM+ eligibility group. **(A)** Mean PHQ-ADS scores over time by PM+ eligibility group (intention-to-treat) with 95% confidence intervals: eligible (*n* = 85) and not eligible (*n* = 10). The not-eligible group remained stable over time. The number of eligible participants per time point was: *t*_1_ = 85, *t*_2_ = 85, *t*_3_ = 71, *t*_4_ = 82. **(B)** Individual PHQ-ADS trajectories for not-eligible participants (*n* = 10).
(DOCX)

S1 Table Baseline sociodemographic, clinical, and COVID-related characteristics of participants by randomisation group. (DOCX)

S2 Table Primary and secondary outcome summary statistics, estimated marginal means, and effect sizes per time point (intention-to-treat, covariate-adjusted linear mixed model). (DOCX)

S3 Table Primary and secondary outcome summary statistics, estimated marginal means, and effect sizes per time point (per-protocol analyses, linear mixed model). (DOCX)

S1 Protocol Full study protocol approved by the Medical Ethics Committee. (PDF)

S1 File Statistical analysis plan. (PDF)

## Abbreviations

ACT: acceptance and commitment therapy
ANOVA: analysis of variance
Castor EDC: Castor Electronic Data Capture
CBT: cognitive behavioural therapy
CONSORT: Consolidated Standards of Reporting Trials
DWM: Doing What Matters in Times of Stress
EU: European Union
EQ-5D-5L: EuroQol 5-dimensional descriptive system—5-level version
IMWs: international migrant workers
ITT: intention-to-treat
LMMs: linear mixed models
MIMIS: Mainz Inventory of Microstressors
PFA: Psychological First Aid
PHQ-ADS: Patient Health Questionnaire-Anxiety and Depression Scale
PM+: Problem Management Plus
PP: per-protocol
PTSD: post-traumatic stress disorder
RCT: randomised controlled trial
SD: standard deviation
SH+: Self-Help Plus
WHO: World Health Organization

## References

[1] International Labour Organization. ILO global estimates on international migrant workers: international migrants in the labour force. Geneva: ILO; 2024. doi: 10.54394/ESKI5420

[2] International Labour Organization. ILO global estimates on international migrant workers: results and methodology. 3rd ed. Geneva: ILO; 2021.

[3] CBS. Werknemers geb. in buitenland; wel/niet ingezeten, persoonskenm.; 2010-2020. 2022. Available from: https://opendata.cbs.nl/statline/#/CBS/nl/dataset/84750NED/table?ts=1610572083494

[4] CBS. Arbeidsdeelname; kerncijfers, 2003-2022. 2022. Available from: https://opendata.cbs.nl/statline/#/CBS/nl/dataset/82309ned/table

[5] Moyce SC, Schenker M. Migrant Workers and Their Occupational Health and Safety. Annu Rev Public Health. 2018;39:351–65. doi: 10.1146/annurev-publhealth-040617-013714 29400993

[6] Roos R, Witteveen AB, Barbui C, Bryant R, Dontsova Z, McDaid D, et al. Psychosocial problems, daily functioning and help-seeking behaviour of international migrant workers in the Netherlands: a qualitative study to inform the adaptation of a scalable stepped-care intervention. Glob Ment Health (Camb). 2025;13:e2. doi: 10.1017/gmh.2025.10110 41496881 PMC12766533

[7] Boufkhed S, Thorogood N, Ariti C, Durand MA. “They treat us like machines”: migrant workers’ conceptual framework of labour exploitation for health research and policy. BMJ Glob Health. 2024;9(2):e013521. doi: 10.1136/bmjgh-2023-013521 38316464 PMC10860016

[8] Urrego-Parra HN, Rodriguez-Guerrero LA, Pastells-Peiró R, Mateos-García JT, Gea-Sanchez M, Escrig-Piñol A, et al. The health of migrant agricultural workers in Europe: a scoping review. J Immigr Minor Health. 2022;24(6):1580–9. doi: 10.1007/s10903-022-01330-y 35133580

[9] Mucci N, Traversini V, Giorgi G, Tommasi E, De Sio S, Arcangeli G. Migrant workers and psychological health: a systematic review. Sustainability. 2019;12(1):120. doi: 10.3390/su12010120

[10] Hasan SI, Yee A, Rinaldi A, Azham AA, Mohd Hairi F, Amer Nordin AS. Prevalence of common mental health issues among migrant workers: a systematic review and meta-analysis. PLoS One. 2021;16(12):e0260221. doi: 10.1371/journal.pone.0260221 34855800 PMC8638981

[11] Pega F, Govindaraj S, Tran NT. Health service use and health outcomes among international migrant workers compared with non-migrant workers: a systematic review and meta-analysis. PLoS One. 2021;16(6):e0252651. doi: 10.1371/journal.pone.0252651 34106987 PMC8189512

[12] Fasani F, Mazza J. A vulnerable workforce: migrant workers in the COVID-19 pandemic. Luxembourg: Publications Office of the European Union; 2020.

[13] Berntsen LE, Skowronek NJ. State-of-the-art research overview of the impact of COVID-19 on migrant workers in the EU and the Netherlands. 2021. Available from: https://repository.ubn.ru.nl/handle/2066/233185

[14] World Health Organization. Doing what matters in times of stress: an illustrated guide. 2020 [cited 22 Jul 2025]. Available from: https://www.who.int/publications/i/item/9789240003927

[15] Dawson KS, Bryant RA, Harper M, Kuowei Tay A, Rahman A, Schafer A, et al. Problem Management Plus (PM+): a WHO transdiagnostic psychological intervention for common mental health problems. World Psychiatry. 2015;14(3):354–7. doi: 10.1002/wps.20255 26407793 PMC4592660

[16] Epping-Jordan JE, Harris R, Brown FL, Carswell K, Foley C, García-Moreno C, et al. Self-Help Plus (SH+): a new WHO stress management package. World Psychiatry. 2016;15(3):295–6. doi: 10.1002/wps.20355 27717271 PMC5032500

[17] Sijbrandij M, de Graaff AM, Cadorin C, Twisk JW, Elsawy M, Ramia JA. World Health Organization’s Scalable Psychological Interventions for Syrian Refugees: an individual participant data meta-analysis of problem management plus and step-by-step. 2025. doi: 10.2139/ssrn.5191106

[18] Karyotaki E, Sijbrandij M, Purgato M, Acarturk C, Lakin D, Bailey D, et al. Self-Help Plus for refugees and asylum seekers: an individual participant data meta-analysis. BMJ Ment Health. 2023;26(1):e300672. doi: 10.1136/bmjment-2023-300672 37524517 PMC10391800

[19] van Straten A, Hill J, Richards DA, Cuijpers P. Stepped care treatment delivery for depression: a systematic review and meta-analysis. Psychol Med. 2015;45(2):231–46. doi: 10.1017/S0033291714000701 25065653

[20] Cuijpers P, Donker T, van Straten A, Li J, Andersson G. Is guided self-help as effective as face-to-face psychotherapy for depression and anxiety disorders? A systematic review and meta-analysis of comparative outcome studies. Psychol Med. 2010;40(12):1943–57. doi: 10.1017/S0033291710000772 20406528

[21] Purgato M, Tedeschi F, Turrini G, Cadorin C, Compri B, Muriago G, et al. Effectiveness of a stepped-care programme of WHO psychological interventions in a population of migrants: results from the RESPOND randomized controlled trial. World Psychiatry. 2025;24(1):120–30. doi: 10.1002/wps.21281 39810692 PMC11733480

[22] Mediavilla R, Felez-Nobrega M, McGreevy KR, Monistrol-Mula A, Bravo-Ortiz M-F, Bayón C, et al. Effectiveness of a mental health stepped-care programme for healthcare workers with psychological distress in crisis settings: a multicentre randomised controlled trial. BMJ Ment Health. 2023;26(1):e300697. doi: 10.1136/bmjment-2023-300697 37263708 PMC10254812

[23] Roos R, Witteveen AB, Ayuso-Mateos JL, Barbui C, Bryant RA, Felez-Nobrega M, et al. Effectiveness of a scalable, remotely delivered stepped-care intervention to reduce symptoms of psychological distress among Polish migrant workers in the Netherlands: study protocol for the RESPOND randomised controlled trial. BMC Psychiatry. 2023;23(1):801. doi: 10.1186/s12888-023-05288-5 37919694 PMC10623706

[24] Hopewell S, Chan A-W, Collins GS, Hróbjartsson A, Moher D, Schulz KF, et al. CONSORT 2025 statement: updated guideline for reporting randomised trials. PLoS Med. 2025;22(4):e1004587. doi: 10.1371/journal.pmed.1004587 40228477 PMC11996237

[25] Kessler RC, Andrews G, Colpe LJ, Hiripi E, Mroczek DK, Normand SLT, et al. Short screening scales to monitor population prevalences and trends in non-specific psychological distress. Psychol Med. 2002;32(6):959–76. doi: 10.1017/s0033291702006074 12214795

[26] Sulaiman-Hill CM, Thompson SC. Selecting instruments for assessing psychological wellbeing in Afghan and Kurdish refugee groups. BMC Res Notes. 2010;3:237. doi: 10.1186/1756-0500-3-237 20825681 PMC2949661

[27] Castor Electronic Data Capture. [cited 23 Jun 2023]. Available from: https://castoredc.com

[28] World Health Organization, War Trauma Foundation, World Vision International. Psychological first aid: guide for field workers. 2011 [cited 22 Jun 2023]. Available from: https://www.who.int/publications-detail-redirect/9789241548205

[29] Bernal G, Chafey M, Domenech Rodríguez M. Cultural adaptation of treatments: a resource for considering culture in evidence-based practice. Prof Psychol Res Pract. 2009;40:361–8. doi: 10.1037/a0016401

[30] Kroenke K, Spitzer RL, Williams JBW. The PHQ-9. J Gen Intern Med. 2001;16:606–13. doi: 10.1046/j.1525-1497.2001.016009606.x 11556941 PMC1495268

[31] Spitzer RL, Kroenke K, Williams JBW, Löwe B. A brief measure for assessing generalized anxiety disorder: the GAD-7. Arch Intern Med. 2006;166(10):1092–7. doi: 10.1001/archinte.166.10.1092 16717171

[32] Ślusarska BJ, Nowicki G, Piasecka H, Zarzycka D, Mazur A, Saran T, et al. Validation of the Polish language version of the Patient Health Questionnaire-9 in a population of adults aged 35-64. Ann Agric Environ Med. 2019;26(3):420–4. doi: 10.26444/aaem/99246 31559797

[33] Okruszek Ł, Aniszewska-Stańczuk A, Piejka A, Wiśniewska M, Żurek K. Safe but lonely? Loneliness, mental health symptoms and COVID-19. 2020. doi: 10.31234/osf.io/9njps PMC7747668 33343454

[34] Dragan M, Grajewski P, Shevlin M. Adjustment disorder, traumatic stress, depression and anxiety in Poland during an early phase of the COVID-19 pandemic. Eur J Psychotraumatol. 2021;12(1):1860356. doi: 10.1080/20008198.2020.1860356 34992743 PMC8725738

[35] Gambin M, Sękowski M, Woźniak-Prus M, Wnuk A, Oleksy T, Cudo A, et al. Generalized anxiety and depressive symptoms in various age groups during the COVID-19 lockdown in Poland. Specific predictors and differences in symptoms severity. Compr Psychiatry. 2021;105:152222. doi: 10.1016/j.comppsych.2020.152222 33388494

[36] Price M, Szafranski DD, van Stolk-Cooke K, Gros DF. Investigation of abbreviated 4 and 8 item versions of the PTSD Checklist 5. Psychiatry Res. 2016;239:124–30. doi: 10.1016/j.psychres.2016.03.014 27137973

[37] Manea L, Gilbody S, McMillan D. Optimal cut-off score for diagnosing depression with the Patient Health Questionnaire (PHQ-9): a meta-analysis. CMAJ. 2012;184(3):E191-6. doi: 10.1503/cmaj.110829 22184363 PMC3281183

[38] Ogińska-Bulik N, Lis-Turlejska M, Juczyński Z. Polish adaptation of the PTSD checklist for DSM-5 - PCL-5. A preliminary communication. Przegl Psychol. 2018;61:287–91.

[39] Schnurr P, Vielhauer M, Weathers F, Findler M. The brief trauma questionnaire (BTQ). US Department of Veterans Affairs; 1999.

[40] World Health Organization. Process of translation and adaptation of instruments. 2018 [cited 23 Jun 2023]. Available from: http://www.who.int/substance_abuse/research_tools/translation/en/

[41] Petri-Romão P, Engen H, Rupanova A, Puhlmann L, Zerban M, Neumann RJ, et al. Self-report assessment of Positive Appraisal Style (PAS): development of a process-focused and a content-focused questionnaire for use in mental health and resilience research. PLoS One. 2024;19(2):e0295562. doi: 10.1371/journal.pone.0295562 38306328 PMC10836662

[42] Chmitorz A, Kurth K, Mey LK, Wenzel M, Lieb K, Tüscher O, et al. Assessment of microstressors in adults: questionnaire development and ecological validation of the mainz inventory of microstressors. JMIR Ment Health. 2020;7(2):e14566. doi: 10.2196/14566 32130154 PMC7063526

[43] Veer IM, Riepenhausen A, Zerban M, Wackerhagen C, Puhlmann LMC, Engen H, et al. Psycho-social factors associated with mental resilience in the Corona lockdown. Transl Psychiatry. 2021;11(1):67. doi: 10.1038/s41398-020-01150-4 33479211 PMC7817958

[44] Herdman M, Gudex C, Lloyd A, Janssen M, Kind P, Parkin D, et al. Development and preliminary testing of the new five-level version of EQ-5D (EQ-5D-5L). Qual Life Res. 2011;20(10):1727–36. doi: 10.1007/s11136-011-9903-x 21479777 PMC3220807

[45] Beecham J, Knapp M. Costing psychiatric interventions. Measuring mental health needs. London, England: Gaskell/Royal College of Psychiatrists. 1992. p. 163–83.

[46] Sheehan DV, Lecrubier Y, Sheehan KH, Amorim P, Janavs J, Weiller E. The Mini-International Neuropsychiatric Interview (M.I.N.I.): The development and validation of a structured diagnostic psychiatric interview for DSM-IV and ICD-10. J Clin Psychiatry. 1998;59:22–33. 9881538

[47] Bryant RA, Schafer A, Dawson KS, Anjuri D, Mulili C, Ndogoni L, et al. Effectiveness of a brief behavioural intervention on psychological distress among women with a history of gender-based violence in urban Kenya: a randomised clinical trial. PLoS Med. 2017;14(8):e1002371. doi: 10.1371/journal.pmed.1002371 28809935 PMC5557357

[48] Rahman A, Hamdani SU, Awan NR, Bryant RA, Dawson KS, Khan MF, et al. Effect of a multicomponent behavioral intervention in adults impaired by psychological distress in a conflict-affected area of Pakistan: a randomized clinical trial. JAMA. 2016;316(24):2609–17. doi: 10.1001/jama.2016.17165 27837602

[49] Rezvan PH, Comulada WS, Fernández MI, Belin TR. Assessing alternative imputation strategies for infrequently missing items on multi-item scales. Commun Stat Case Stud Data Anal Appl. 2022;8(4):682–713. doi: 10.1080/23737484.2022.2115430 36467970 PMC9718541

[50] Buuren Sv, Groothuis-Oudshoorn K. mice: multivariate imputation by chained equations in R. J Stat Softw. 2011;45: 1–67. doi: 10.18637/jss.v045.i03

[51] de Boer MR, Waterlander WE, Kuijper LDJ, Steenhuis IHM, Twisk JWR. Testing for baseline differences in randomized controlled trials: an unhealthy research behavior that is hard to eradicate. Int J Behav Nutr Phys Act. 2015;12:4. doi: 10.1186/s12966-015-0162-z 25616598 PMC4310023

[52] Jacobson NS, Truax P. Clinical significance: a statistical approach to defining meaningful change in psychotherapy research. Methodological issues & strategies in clinical research. Washington, DC, US: American Psychological Association; 1992. p. 631–48. doi: 10.1037/10109-042 2002127

[53] Kessler R, Barker P, Colpe L, Epstein J, Gfroerer J, Hiripi E. The Kessler Psychological Distress Scale (K10). Harvard Medical School; 1996.

[54] Schäfer SK, Thomas LM, Lindner S, Lieb K. World Health Organization’s low-intensity psychosocial interventions: a systematic review and meta-analysis of the effects of Problem Management Plus and Step-by-Step. World Psychiatry. 2023;22(3):449–62. doi: 10.1002/wps.21129 37713578 PMC10503931

[55] de Graaff AM, Cuijpers P, Twisk JWR, Kieft B, Hunaidy S, Elsawy M, et al. Peer-provided psychological intervention for Syrian refugees: results of a randomised controlled trial on the effectiveness of Problem Management Plus. BMJ Ment Health. 2023;26(1):e300637. doi: 10.1136/bmjment-2022-300637 36789918 PMC10035776

[56] Johns RG, Barkham M, Kellett S, Saxon D. A systematic review of therapist effects: a critical narrative update and refinement to review. Clin Psychol Rev. 2019;67:78–93. doi: 10.1016/j.cpr.2018.08.004 30442478

[57] Zhang M, Fan C, Ma L, Wang H, Zu Z, Yang L, et al. Assessing the effectiveness of internet-based interventions for mental health outcomes: an umbrella review. Gen Psychiatr. 2024;37(4):e101355. doi: 10.1136/gpsych-2023-101355 39040128 PMC11261690

[58] Forbes A, Keleher MR, Venditto M, DiBiasi F. Assessing patient adherence to and engagement with digital interventions for depression in clinical trials: systematic literature review. J Med Internet Res. 2023;25:e43727. doi: 10.2196/43727 37566447 PMC10457707

[59] Krieg S, van Rooijen J. Poolse werknemers in Nederland: de nieuwkomers van 2011 op de voet gevolgd. CBS; 2018.

[60] Kamper SJ. Per-protocol, intention-to-treat, and complier average causal effects analyses in randomized controlled trials: linking evidence to practice. J Orthop Sports Phys Ther. 2021;51:314–5. 34058836 10.2519/jospt.2021.0701

