# Supplementary material for: Effectiveness of a scalable, remotely delivered stepped-care intervention for psychological distress among Polish migrant workers in the Netherlands: The RESPOND randomised controlled trial
Source: PLoS Med. 2026 Aug 17;23(8):e1005197. doi: 10.1371/journal.pmed.1005197 (PMC13480649; doi:10.1371/journal.pmed.1005197)
Supplement: Supplementary file 1 [file pmed.1005197.s006.pdf]

RESEARCH PROTOCOL RESPOND

Improving mental healthcare for labour migrants in the Netherlands during the COVID-19 pandemic: implementation of a stepped care program (DWM/PM+)

PROTOCOL TITLE ***Improving mental healthcare for labour migrants in the Netherlands during the COVID-19 pandemic***

**Protocol ID**	***<include protocol ID given by sponsor or investigator>***
**Short title**	***Implementation of a stepped care program in labour migrants***
**Version**	**10**
**Date**	**02-07-2023**
**Coordinating investigator/project leader**	***Dr. M. Sijbrandij, Professor***<br>***VU University, Department of Clinical, Neuro- and Developmental Psychology***<br>***Van der Boechorststraat 1***<br>***1081 BT Amsterdam***
**Main investigator(s):**	***Dr A.B. Witteveen, Assistant Professor***<br>***VU University, Department of Clinical, Neuro- and Developmental Psychology***<br>***Van der Boechorststraat 1***<br>***1081 BT Amsterdam***<br><br>***R. Roos, PhD student***<br>***VU University, Department of Clinical, Neuro- and Developmental Psychology***<br>***Van der Boechorststraat 1***<br>***1081 BT Amsterdam***
**Sponsor (in Dutch: verrichter/opdrachtgever)**	***VU University, Department of Clinical, Neuro- and Developmental Psychology***<br>***European Commission, Horizon 2020 funding Scheme***
**Subsidising party**	***VU University, Department of Clinical, Neuro- and***

	Developmental Psychology
**Independent expert (s)**	Dominika Chromik<br><br>***GGZ Keizersgracht***<br><br>**Keizersgracht 178**<br><br>***1016 DW Amsterdam***
**Laboratory sites**	***Lab for analysis of hair samples:***<br>***Dresden LabService GmbH***<br><br>***Tatzberg 47-49***<br><br>***01307 Dresden***<br><br>***Germany***
**Pharmacy** ***<if applicable>***	***Not applicable***

PROTOCOL SIGNATURE SHEET

Name	Signature	Date
**Sponsor or legal representative:**<br>VU Amsterdam<br>Department Clinical Psychology<br>+31(0)20 59 88951<br><br>**Head of Department:**<br>Prof. Dr. Annemieke van Straten<br>Professor Clinical Psychology<br>VU Amsterdam University		
**Coordinating Investigator/Project leader/Principal Investigator:**<br>Prof. Dr. Marit Sijbrandij<br>Associate Professor<br>Clinical Psychology<br>VU Amsterdam University		

TABLE OF CONTENTS

LIST OF ABBREVIATIONS AND RELEVANT DEFINITIONS 7
SUMMARY 9
1. INTRODUCTION AND RATIONALE 12
2. OBJECTIVES 17
3. STUDY DESIGN 18
4. STUDY POPULATION 22
4.1 Population (base) 22
4.2 Inclusion criteria 22
4.3 Exclusion criteria 22
4.4 Sample size calculation 23
5. TREATMENT OF SUBJECTS 24
5.1 Investigational product/treatment 25
5.2 Use of co-intervention (if applicable) 33
5.3 Escape medication (if applicable) 33
6. METHODS 34
6.1 Study parameters/endpoints 34
6.1.1 Main study parameter/endpoint 34
6.1.2 Secondary study parameters/endpoints (if applicable) 34
6.1.3 Other study parameters (if applicable) 35
6.2 Randomisation, blinding and treatment allocation 35
6.3 Study procedures 36
Withdrawal of individual subjects 55
6.4.1 Specific criteria for withdrawal (if applicable) 55
6.5 Replacement of individual subjects after withdrawal 55
6.6 Follow-up of subjects withdrawn from treatment 55
6.7 Premature termination of the study 56
7. SAFETY REPORTING 57
7.1 Temporary halt for reasons of subject safety 57
7.2 AEs, SAEs and SUSARs 57
7.2.1 Adverse events (AEs) 57
7.2.2 Serious adverse events (SAEs) 57
7.3 Follow-up of adverse events 58
7.4 Ethics and Data Advisory Board (EDAB) 58
8. STATISTICAL ANALYSIS 60
8.1 Primary study parameter(s) 60
8.2 Secondary study parameter(s) 61
8.3 Other study parameter(s) 62
8.4 Interim analysis (if applicable) 62
9. ETHICAL CONSIDERATIONS 63
9.1 Regulation statement 63
9.2 Recruitment and consent 63

9.3 Objection by minors or incapacitated subjects (if applicable) 67
9.4 Benefits and risks assessment, group relatedness 67
9.5 Compensation for injury 68
9.6 Incentives (if applicable) 68
9.7 Handling and storage of data and documents 70
9.8 Monitoring and Quality Assurance 72
9.9 Amendments 72
9.10 Annual progress report 73
9.11 Temporary halt and (prematurely) end of study report 73
9.12 Public disclosure and publication policy 73
REFERENCES 74

LIST OF ABBREVIATIONS AND RELEVANT DEFINITIONS

**ABR**	**General Assessment and Registration form (ABR form), the application form that is required for submission to the accredited Ethics Committee; in Dutch: Algemeen Beoordelings- en Registratieformulier (ABR-formulier)**
**AE**	**Adverse Event**
**AR**	**Adverse Reaction**
**BTQ**	**Brief Trauma Questionnaire**
**CSRI**	**Client Service Receipt Inventory**
**DHEA(S)**	Dehydroepiandrosterone (sulfate) (DHEA(S))
**DSM**	**Diagnostic and Statistical Manual of Mental Disorders**
**DWM**	**Doing What Matters in Times of Stress**
**EDAB**	**Ethics and Data Advisory Board**
**EU**	**European Union**
**EQUIP REMOTE**	**Ensuring Quality in Psychological Support (WHO, remote version)**
**GAD-7**	**Generalized Anxiety Disorder – 7**
**GCP**	**Good Clinical Practice**
**GDPR**	**General Data Protection Regulation; in Dutch: Algemene Verordening Gegevensbescherming (AVG)**
**GP**	**General practitioner**
**HCC**	**Hair cortisol concentrations**
**IC**	**Informed Consent**
**K10**	**Kessler Psychological Distress Scale (ten item version)**
**MIMIS**	**Mainz Inventory of MIcrostressorS**
**PASSc**	**Positive Appraisal Style Scale – content focused**
**PCL-5**	**PTSD Checklist for DSM-5 (consisting of 20 items)**
**PHQ-9**	**Patient Health Questionnaire for depression scoring each of the 9 DSM-5 criteria**
**PHQ-ADS**	**Patient Health Questionnaire – Anxiety and Depression (sum score of PHQ-9 and GAD-7)**
**PTSD**	**Posttraumatic Stress Disorder**
**RESPOND**	**pREparednesS of health systems to reduce mental health and Psychosocial concerns resulting from the COVID-19 paNDemic**
**PM+**	**Problem Management Plus**

**RCT**	**Randomized Controlled Trial**
**SH+**	**Self Help Plus**
**Sponsor**	**The sponsor is the party that commissions the organisation or performance of the research, for example a pharmaceutical company, academic hospital, scientific organisation or investigator. A party that provides funding for a study but does not commission it is not regarded as the sponsor, but referred to as a subsidising party.**
**WMO**	**Medical Research Involving Human Subjects Act; in Dutch: Wet Medisch-wetenschappelijk Onderzoek met Mensen**

SUMMARY

Rationale: The ongoing COVID-19 pandemic has a major and potentially long-lasting effect on mental health and wellbeing across populations worldwide. Vulnerable groups, such as labour migrants, are disproportionally affected by the COVID-19 pandemic. There is a high need for psychosocial interventions that can target the most prevalent mental health problems arising from the COVID-19 pandemic, addressing the needs of many people in a way that maximises the use of resources. The World Health Organization (WHO) has developed two scalable, low-intensity psychological interventions: Doing What Matters in times of stress (DWM; a self-help intervention) and Problem Management Plus (PM+; a face-to-face intervention). DWM and PM+ can be delivered by non-specialist helpers, are applicable to a variety of mental health problems (depression, anxiety and PTSD), and can be adapted to different populations, cultures and languages. Both DWM and PM+ have been proven to be effective on their own. In this study, DWM and PM+ will be combined into a stepped-care intervention (DWM/PM+). This study is part of the larger EU H2020-RESPOND project, which aims to improve the preparedness of European mental health care systems in the face of future pandemics.

Objective: The main objective is to evaluate the implementation and (cost-)effectiveness of the culturally and contextually adapted DWM/PM+ stepped-care programs amongst labour migrants living in the Netherlands during the COVID-19 pandemic in terms of mental health outcomes, resilience, wellbeing, health inequalities, and costs to health systems. The main hypothesis is that the stepped-care DWM/PM+ intervention together with psychological first aid (PFA), in addition to care-as-usual (CAU), will be more effective in decreasing psychological distress and symptoms of mental health problems than PFA and CAU alone. This study consists of 3 phases. The objective of phase 1 is to adapt the DWM/PM+ manuals to the culture and context of labour migrants (protocol approved by VCWE). The objective of phase 2 is to assess the (cost-)effectiveness of the stepped-care DWM/PM+ intervention. The objective of phase 3 is to identify (a) barriers and facilitators to treatment engagement and adherence and (b) opportunities for scaling up the implementation of the DWM/PM+ intervention within the existing health care system in labour migrants. The current protocol focuses on phases 2 and 3.

Study design: Study phase 2: pragmatic implementation trial with a single-blinded, randomised, parallel-group design. Study phase 3: qualitative process evaluation consisting of individual interviews and focus group discussions (FGDs).

Study population: Study phase 2: Adult labour migrants with self-reported elevated psychological distress (K10 >15.9) (n=212). Study phase 3: Various participants: (a) participants in study phase 2 who completed DWM and improved (n=4), who completed PM+ and improved (n=4), who completed PM+ and not improved (n=4), who dropped-out during DWM (n=4), and who dropped-out

during PM+ (n=4); (b) family members/close persons of participants in study phase 2 who completed the intervention (n=6) and who dropped-out during the intervention (n=6); (c) professionals (n=20-25) (e.g. mental health practitioners and local stakeholders of participating centres, clinical staff in primary and secondary care, local & national policy makers); (d) facilitators of the DWM and PM+ intervention (both helpers and trainers/supervisors) (n=8-16).

Intervention (if applicable): All participants (in both the treatment and comparison groups) will receive Psychological First Aid (PFA) (by telephone) and CAU. In addition to PFA and CAU, the treatment group will receive the stepped-care intervention (DWM with or without PM+). The stepped-care intervention consists of DWM (step 1) and conditionally PM+ (step 2) if participants still meet criteria for psychological distress (K10 >15.9) 1 week after having received DWM (week 6 after baseline). DWM, i.e. a self-help book with brief audio exercises to support practice, will be adapted into a digital tool or application (phase 1) and delivered as a guided self-help intervention with support from a briefly trained non-specialist helper via teleconferencing. PM+ consists of five sessions and will be delivered by trained non-specialist helpers in person or via teleconferencing in individual (or group) format. In addition to PFA, the comparison group will only receive CAU which ranges from community care to specialised psychological treatments.

Main study parameters/endpoints: Study phase 2: Screening for inclusion and exclusion criteria will be interviewer-administered through (video) calls. After screening, online assessments will take place in all participants at four time-points: at baseline, at week 6 (about 1 week after having received DWM), and at week 12 and week 20 (respectively about 1 week and 2 months after the PM+ intervention has been finished). The primary outcome will be the decrease in symptoms of anxiety and depression from baseline to two-month follow-up, measured through the sum score of the Patient Health Questionnaire (PHQ-9) and General Anxiety Disorder-7 (GAD-7), i.e. the PHQ-Anxiety and Depression Score (PHQ-ADS). We expect to detect a Cohen’s d effect size of 0.3 in the PM+ group at 2 months post-treatment. Additional health outcomes include level of anxiety (GAD-7) and depression (PHQ-9), symptoms of posttraumatic stress disorder (PCL-5), resilience (Mainz Inventory of MIcrostressors, MIMIS) and positive appraisal style (Positive Appraisal Style Scale – content focused, PASSc), quality of life (EQ-5D-5L), and costs of care (CSRI). Additional study parameters will include demographic data, traumatic and COVID-19 related (exposure) variables (Brief Trauma Questionnaire, BTQ), hair biomarkers (cortisol concentrations (HCC), cortisone, dehydroepiandrosterone (sulfate) (DHEA(S)), progesterone, and testosterone concentrations), digital markers, treatment fidelity, and implementation indicators (such as reach, dose, resource use, intervention implementation related costs). Study phase 3: Through FGDs and interviews at the end of the study, the feasibility of scaling-up the implementation on the stepped-care DWM/PM+ intervention within the Netherlands will be examined.

Nature and extent of the burden and risks associated with participation, benefit and group relatedness: The burden and risks resulting from participation in this study are minimal in view of the naturalistic design, inclusion of only participants with mild to moderate functional impairment and psychological distress and minimal duration of the various questionnaire measurements (approx. 45-60 minutes) and interviews. Study phase 2: participants will be assessed (online and over the telephone) a total of four times over the course of six months. Data will be collected by means of self-administered (online) questionnaires. If participants give permission, the six helper support calls in DWM and five PM+ sessions, for participants in the treatment group who still meet inclusion criteria after DWM, will be respectively audiotaped and videotaped for the purpose of supervision and monitoring. Additionally, digital markers will be explored for participants attending PM+ sessions. Hair samples will be collected to measure hair cortisol, cortisone, DHEAS, progesterone, and testosterone concentrations. Study phase 3: The interviews with participants in the target group in phase 2 (both completers and drop-outs), their family members/close persons, and professionals will take approximately 1.5 hours. The FGDs with DWM/PM+ facilitators will take approximately 2 hours.

1. INTRODUCTION AND RATIONALE

Impact of pandemic and containment measures

The ongoing COVID-19 pandemic has a major and potentially long-lasting effect on mental health and wellbeing across populations in Europe and worldwide. The pandemic has affected every country in the world, with some of the most deeply affected countries in Europe, including the Netherlands (John Hopkins University and Medicine, 2020). Early reports regarding the levels of psychological distress associated with the COVID-19 crisis are highly concerning (McGinty, Presskreischer, Han & Barry, 2020) and led to the UN releasing a policy briefing on the mental health impacts of COVID-19 warning that a 'long-term upsurge in the number and severity of mental health problems is likely' (United Nations, 2020). European governments installed epidemic control measures to contain the spreading of the virus, which include lockdown restrictions and closures (amongst others) that have created a great impact on society (Blavantnik School of Government, University of Oxford, 2020). Physical distancing and staying at home orders have led to elevated loneliness in many people, which is in turn associated with depression and suicidal ideation (Stickley & Koyanagi, 2016). Despite social protection programs, many individuals have directly been impacted financially by the economic consequences of the containment measures, and have seen their incomes decline, especially people with short-term or part-time contracts, or those being self-employed. Economic estimates show that the lockdown will directly affect sectors amounting to up to a loss of 2 percentage points in annual GDP growth for each month of containment (OECD, 2020). Following economic recession including during periods of economic recovery, mental health problems have been shown to increase (Garcy & Vagero, 2013).

Focus on vulnerable groups

Although the COVID-19 pandemic has affected the whole population, the consequences are not distributed equally. COVID-19 has had a larger effect on people living under fragile circumstances, and on socio-economically disadvantaged and minority populations across Europe. The risk of dying from COVID-19 is higher among people in socio-economically deprived areas than among people in the least deprived areas (Public Health England, 2020). Furthermore, historically, following economic recession, higher increases in mental health symptoms of younger working-age people have been found (Thomson & Katikireddi, 2018). Thus, the COVID-19 pandemic and the associated containment measures are expected to lead to an exacerbation of health inequalities in Europe and beyond. In May 2020, the WHO already stated that particular attention should be given to vulnerable groups, such as informal or self-employed people, health care workers, people with pre-existing (mental) health conditions, women and children exposed to abuse or violence, and migrants (WHO, 2020b).

Labour migrants in the Netherlands

Labour migrants are believed to constitute a potentially fragile population in the Netherlands. In 2019, there were 958,000 labour migrants. Over half of these labour migrants came from the European Union (EU) (485,000 people; CBS, 2020). Most labour migrants work within agriculture or horticulture, the food industry, logistics and distribution sector, and the construction industry. Many labour migrants arrive in the Netherlands with the help of an employment agency. Often, these agencies not only arrange work, but also housing, transport, and health insurance. As a result, labour migrants are highly dependent on their employment agency; losing their job often means that they lose their housing and health insurance as well. Some employment agencies house far too many people in poorly maintained houses (e.g. sharing bedrooms with multiple people), or let people work in unsafe working conditions. Due to their dependency of their employer, labour migrants may prefer not to report malpractices regarding their work or living situation or call-in sick, out of fear to lose their work or housing. Moreover, many labour migrants are not self-reliant: there is a language barrier, they are ill-informed of Dutch laws and their rights, they do not know how to find their way between the various institutions, they do not have a social safety net, and have a vulnerable negotiating position (Aanjaagteam Bescherming Arbeidsmigranten, 2020).

Already at the beginning of the COVID-19 crisis, the vulnerable position of labour migrants in the Netherlands became even more apparent. Many labour migrants were unable to attain to the COVID-19 regulations as they were unable to keep appropriate distance from other people at home, when traveling, or at work. In combination with a regularly varying composition of house mates and working on multiple locations, this resulted in various outbreaks, such as in slaughterhouses. Labour migrants who contracted COVID-19 got fired and therefore became homeless, which resulted in them having to turn to emergency shelters or living on the street without health insurance (Aanjaagteam Bescherming Arbeidsmigranten, 2020). As a result, the Dutch cabinet requested for a so-called booster team to be formed for the protection of labour migrants. The goal of this team was twofold: (1) to lower the risk for labour migrants to become infected with COVID-19 in the short-term, and (2) to improve the position of labour migrants in the Netherlands in the long-term, by making proposals to improve the work and living situation of labour migrants. While doing research, the booster team found that there is a group of labour migrants who struggle with uncertainty and psychological complaints (Aanjaagteam Bescherming Arbeidsmigranten, 2020).

Labour migrants may experience various barriers to receiving (mental) health care. First, considering that many labour migrants work through an employment agency that may have arranged health insurance for them, they lose their insurance the moment they lose their job. Second, even if labour migrants do have health insurance arranged by their employer, the employer may not have given them their health insurance card or policy, which is proof of being insured. Not having received this in combination with a lack of knowledge of Dutch health insurance, labour

migrant may be uncertain as to whether they are being insured or not and thus not utilize health care. Without DigiD, they are also unable to check this themselves. Third, considering that many labour migrants are in the Netherlands only for a brief amount of time, many are not registered with a general practitioner (Aanjaagteam bescherming arbeidsmigranten, 2020). In fact, some general practitioners do not want to register labour migrants at their practice, because these migrants only live somewhere for a limited amount of time (Aanjaagteam bescherming arbeidsmigranten). Because of these barriers, labour migrants may not seek care and as a result their complaints may worsen causing them to receive the appropriate care too late (Aanjaagteam Bescherming Arbeidsmigranten, 2020).

Scalable psychological interventions to improve resilience, mental health and wellbeing
There is a high need for psychological interventions for risk groups, such as labour migrants, affected by the COVID-19 pandemic to address the most prevalent mental health problems as a result of the COVID-19 pandemic, most notably symptoms of distress, anxiety, depression, and posttraumatic stress disorder (PTSD). Telehealth has proven to be effective for delivery of individual or group-based mental health and psychosocial support interventions (Banbury, Nancarrow, Dart, Gray & Parkinson, 2018), and has great advantages over regular mental health care delivery in terms of its feasibility under lockdown restrictions, scalability, and reducing costs. This can be particularly useful for labour migrants in the Netherlands who experience various barriers to (mental) health care. However, it is yet unclear whether it has met the needs of more vulnerable people with specific needs, who are illiterate, older, or people without good quality internet access (EuroHealthNet, 2020).

The World Health Organization (WHO) has developed a number of scalable psychological interventions for populations affected by adversity (WHO, 2017). They include -amongst others- - Doing What Matters in Times of Stress (DWM) and Problem Management Plus (PM+). A core feature of all WHO scalable interventions is that they can be trained to and delivered by non-professional helpers, such as a trained peer, or helper at the workplace, or a psychosocial worker (task-shifting) (WHO, 2017; Epping-Jordan et al., 2016). They have also been designed to be widely applicable to a variety of mental health problems (depression, anxiety and PTSD) and easily adaptable to different populations, cultures and languages. Finally, the interventions and their implementation materials are freely available on the WHO website.

DWM was originally designed for flexible implementation either in a group or individual format including an online format. DWM is a self-help stress management illustrated guide that is one part of Self-Help Plus (SH+) and can be delivered individually with support from a briefly trained non-specialised helper. The illustrated DWM guide comprises the core information from the SH+ course and is accompanied by audio files to support regular practice of exercises which enhance stress

reduction and adaptive coping and resilience (WHO, 2020a). SH+ and DWM are based on acceptance and commitment therapy (ACT), a modern form of cognitive-behavioural therapy with a strong focus on mindfulness practices and includes exercises which aim to enhance stress reduction and build social support, adaptive coping and resilience (Epping-Jordan et al., 2016). SH+ has been implemented with different populations of refugees in Europe, Turkey (Purgato et al., 2019) and Northern Uganda (Tol et al., 2020). Results of the study in Northern Uganda showed stronger improvement on psychological distress in the SH+ intervention group than in the control group. Results from the study in Turkey have not yet been published. In RESPOND, the DWM pre-recorded audios and the illustrated stress management guide, will be culturally adapted to an online application (app) (Phase 1). This way, people can use the self-help online app in their own time. The five weekly sessions in the app will follow the 5 chapters of the book (grounding, unhooking, acting on your values, being kind and making room). Participants will receive a weekly call from a non-specialist helper, trained to support and motivate individuals using the app.

PM+ is a transdiagnostic intervention (Dawson et al., 2015) that reduces symptoms of depression, anxiety, PTSD, and related conditions, is delivered by trained non-specialised workers or lay people, and is available in individual and group delivery formats. It was originally designed for in-person delivery, but similarly to other psychological interventions, can be adapted for delivery over the telephone or video-conferencing . PM+ comprises 5 weekly sessions using evidence-based techniques: of (a) problem solving, (b) stress management, (c) behavioural activation, and (d) accessing social support. Individual PM+ has been successfully implemented in Kenya (Bryant et al., 2017) and Pakistan (Rahman et al., 2016b). Both SH+ and PM+ have been implemented through two large EU H2020 funded projects, STRENGTHS (733337) and RE-DEFINE (779255). For this study, we will adapt both the DWM and the PM+ intervention to the COVID-19 pandemic and to the context and culture of the target population, i.e. we will adapt examples and pictures used in the intervention (Phase 1).

RESPOND Project

This study is embedded in the larger, EU H2020 CORONAVIRUS-funded RESPOND (PREparednesS of health systems to reduce mental health and Psychosocial concerns resulting from the COVID-19 paNDemic) project. The RESPOND project aims to improve the preparedness of the European mental health care system in the face of future pandemics. In order to do that a consortium of eight countries (BE, DE, ES, FR, NL, IT, SWE, UK) will collaborate in:

1) Identifying risk groups for poor wellbeing and mental health related to the COVID-19 pandemic by analysing longitudinal epidemiological data and identifying resilience factors related to the COVID-19 pandemic;

2) Examining the impact of the COVID-19 pandemic on long-term health and mental health services use, and the associations between health and mental health care use and socio-economic factors across European countries with different control measures and pandemic course (Sweden, Italy and Spain);

3) Performing rapid and recurring holistic assessments of policies and measures across Europe that have and will address different phases of the COVID-19 pandemic to protect the mental health and wellbeing of groups at high-risk for COVID-19 related mental health issues

4) Examining the necessary health system and wider contextual and policy conditions for successful implementation and scaling-up of remote-delivered stepped-care using existing, scalable World Health Organization (WHO) programs to improve resilience, wellbeing, overall functioning and mental health, across frontline workers and other high-risk groups for COVID-19 related poor mental health.

5) Providing regional and national health care authorities, including relevant public health and long-term care authorities, with transferable evidence-based practices, methodologies and guidance for scaling up mental health and broader social and economic support measures for frontline workers and other risk groups for COVID-19 related mental health symptoms.

This study(-protocol) contributes to aim number 4; examining the feasibility and cost-effectiveness of remote-delivered scalable interventions for targeting COVID-19-related psychological distress and mental health issues. Both SH+ and PM+ have been found to be effective (Purgato et al., 2019; Tol et al., 2020; Bryant et al., 2017; Rahman et al., 2016b), but it is unknown if the illustrated guide of SH+ - Doing What Matters in Times of Stress (DWM) combined with PM+ are effective as a stepped-care program compared to care-as-usual. Within the Netherlands, the targeted vulnerable group to examine the effectiveness of the stepped-care intervention, will be labour migrants. The treatment group will be compared to an enhanced care-as-usual (CAU) only group in terms of a number of health and implementation outcome variables.

Two very similar study protocols that investigated the effectiveness of PM+ in the Netherlands with the Syrian population (STRENGTHS project) and with refugee adolescents (REMAIN project) have previously been approved by the METc (respectively NL 61361.029.17 and NL72668.029.29). The principal investigator of this study, prof. dr. Marit Sijbrandij, is also the principal investigator and the coordinator of the STRENGTHS and REMAIN projects.

2. OBJECTIVES

Primary Objective: To evaluate the (cost-)effectiveness, feasibility, and acceptability of the culturally and contextually adapted DWM/PM+ stepped-care program among labour migrants in the Netherlands during the COVID-19 pandemic in terms of mental health outcomes, resilience, wellbeing, health inequalities, and costs to health systems.

Secondary Objective: To identify barriers and facilitators to treatment engagement and adherence and opportunities for scaling up among the target population in the Netherlands (study phase 3 – qualitative part of the study).

3. STUDY DESIGN

The complete study consists of three phases: two qualitative studies (study phase 1 and 3) and a single-blind randomized controlled trial (RCT) (study phase 2), see Box 1. This proposal concerns study phase 2 and 3. Study phase 1 is described in a separate non-WMO protocol for which ethical approval has already been provided by the scientific and ethical review board (Vaste Commissie Wetenschap en Ethiek (VCWE), dd. 18-01-2021, protocol nr. VCWE-2021-006) of the faculty of Behavioral and Movement Sciences, Vrije Universiteit Amsterdam.

<u>Study phase 1:</u> Cultural and contextual adaptation of DWM and PM+ for labour migrants in the Netherlands during the COVID-19 pandemic<br><u>Study phase 2:</u> Randomized controlled trial (stepped-care DWM/PM+ intervention with Psychological First Aid and care-as-usual vs. Psychological First Aid and care-as-usual alone)<br><u>Study phase 3:</u> Process evaluation with qualitative interviews and focus groups to assess barriers and facilitators of engagement and adherence to the stepped-care intervention and opportunities for scaling up the implementation of the intervention.

Box 1. Study Phases for RESPOND WP5

Study phase 1

Study phase 1 consists of interviews and focus group discussions with key informants such as people from the target population and professionals, e.g. (mental) health care workers and policy makers, to culturally and contextually adapt the DWM and PM+ interventions. Both interventions will also be adapted in order to suit the stepped-care design of the intervention. Furthermore, this study informs the second phase as to how the DWM/PM+ intervention can best be implemented.

Study phase 2

In Study phase 2 the DWM/PM+ programs will be evaluated. Participants will be screened for elevated distress (K10; see Figure 1). We will conduct a RCT in labour migrants with increased psychological distress to determine whether the stepped care intervention (i.e. DWM/PM+) leads to stronger decreases in mental health outcomes, and increase in wellbeing among compared to care-as-usual (CAU). This phase is designed as a randomised, single-blind parallel-group trial with one treatment group (n=106) and one comparison group (n=106). All participants in both the treatment and the comparison group will receive Psychological First Aid (PFA) and CAU. In addition to PFA and CAU, participants randomised into the treatment group will receive the DWM/PM+ stepped-care intervention, while participants randomised in the comparison group will receive PFA and CAU only.

All participants in the treatment group (i.e. those who receive DWM and PM+ and those who only receive DWM because symptoms subside) will be followed for a period of 2 months after the end of the PM+ session (see Figure 1 for assessment points).

Study phase 3

Study phase 3 is a qualitative study, consisting of interviews and/or focus group discussions among key stakeholders to evaluate barriers and facilitators to treatment engagement and adherence to the DWM/PM+ stepped-care intervention, as well as opportunities for scaling up the implementation of the intervention within the existing healthcare system in the Netherlands . This will inform partners in RESPOND of the synthesis and dissemination of the DWM/PM+ stepped-care intervention for vulnerable groups during a pandemic.

Key stakeholders include (a) participants in the RCT in study phase 2 who completed DWM (n=4; improved), who completed PM+ (n=8; improved (n=4) and not improved (n=4)), who dropped-out during DWM (n=4), and who dropped-out during PM+ (n=4); (b) their family members/close persons of participants in study phase 2 who completed the intervention (n=6) and who dropped-out during the intervention (n=6); (c) professionals (n=20-25) (e.g. mental health practitioners and local stakeholders of participating centres, clinical staff in primary and secondary care, local & national policy makers); (d) facilitators of the DWM and PM+ intervention (both helpers and trainers/supervisors) (n=8-16).

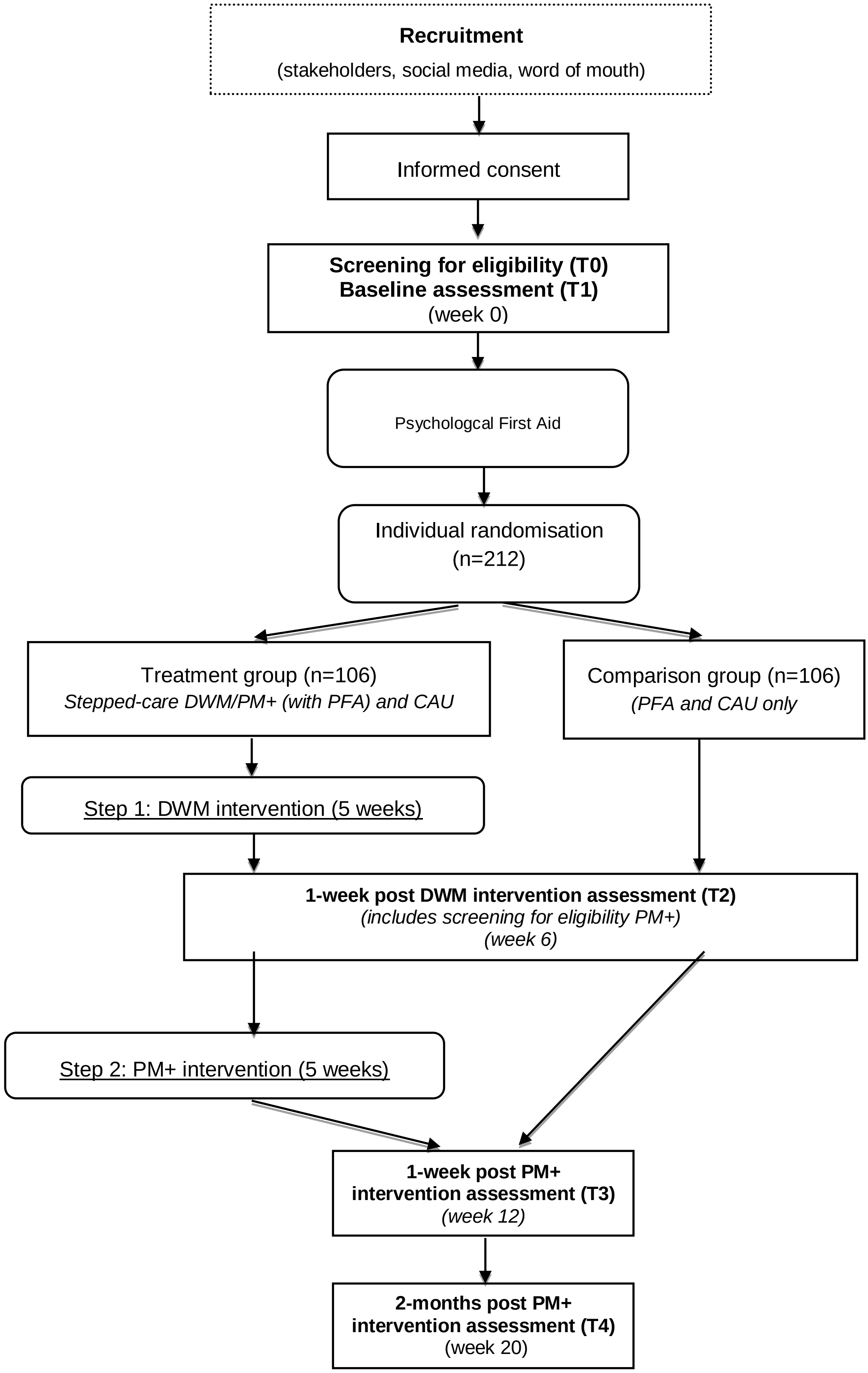

Figure 1. Flowchart of randomized controlled trial (study phase 2)

4. STUDY POPULATION

4.1 Population (base)

Study phase 2

Participants for the RCT will be labour migrants in the Netherlands. According to data of the Central Bureau of Statistics (CBS, 2020), there were 958,000 labour migrants living in the Netherlands in 2019. Participants will be recruited from the general population through (a) targeted social media recruitment, (b) key stakeholders who took part in the recruitment of study phase 1, and (c) other community-based organisations offering psychosocial support for this vulnerable group.

Study phase 3

Participants for the qualitative process evaluation will be key informants, such as participants who took part in the RCT; family members/close friends of participants who took part in the RCT; DWM/PM+ facilitators (helpers and supervisors); mental health professionals and decision makers (recruited through participating centres in study phase 2). For participants who took part in the RCT, we aim to include both those who took part only in DWM and those who took part in PM+ as well. Also drop-outs from both the DWM and PM+ intervention will be asked to participate in the qualitative process evaluation.

4.2 Inclusion criteria

Participants will be eligible to participate in the study (phase 2 (and 3)) if they meet all of the following criteria:

- 18 years or older;
- Living in the Netherlands as labour migrant
- Having elevated levels of psychological distress (Kessler Psychological Distress Scale (K10) >15.9).
- Sufficient mastery (written and spoken) of one of the languages the DWM/PM+ intervention is being delivered in (e.g. English, Polish).
- Having access to an electronic device with internet access and webcam to follow the intervention
- Oral and written informed consent before entering the study.

4.3 Exclusion criteria

Potential participants who meet the inclusion criteria will be excluded from participation in this study (phase 2 (and 3)) if they meet any of the following criteria:

- Planning to permanently move abroad, e.g. back to their home country or to another country, before the last quantitative assessment at 2 months after PM+
- Having acute medical conditions (requiring hospitalisation);
- Imminent suicide risk, or expressed acute needs or protection risks that require immediate follow-up
- Having a severe mental disorder (e.g. psychotic disorders, substance-dependence);
- Having severe cognitive impairment (e.g. severe intellectual disability or dementia);
- Currently receiving specialised psychological treatment (e.g. EMDR, CBT);
- In case of current psychotropic medication use: being on an unstable dose for at least 2 months or a change in dosage over the past 2 months.

4.4 Sample size calculation

A total number of 265 participants will be included; 212 participants will take part in the RCT of study phase 2, and 53 participants will take part in the qualitative study phase 3. Based on prior studies on a PM+ intervention (Bryant et al., 2017; Rahman et al., 2016b), we aim to detect a small to medium Cohen's *d* effect size of 0.3 in the PM+ group at 2 months post-treatment based on the primary composite outcome PHQ-ADS (Kroenke et al., 2016; 2019). The PHQ-ADS is the combined sum score of depression and anxiety symptoms of the PHQ-9 and GAD-7, respectively and has shown good internal consistency (α = .88 to .92) (Kroenke et al., 2016; 2019). A power calculation for a repeated measurement design (with STATA) suggests a minimum sample size of N=74 per group (power=0.80, alpha=0.05, two-sided, rho=0.9). Taking into account 30% attrition, we aim to include a total number of 212 participants (106 in the stepped-care DWM/PM+ treatment group (with PFA and CAU) and 106 in the PFA and CAU comparison group).

A sample size of n=265 is considered feasible, because (a) there are many labour migrants in the Netherlands and (b) we have built a network of organizations working with labour migrants. Over the past decade, the number of employees in the Netherlands that were born abroad has grown from 661.500 (in 2010) to 958.400 (in 2019) (CBS, 2020). More than half of these employees in 2019 (485.000 out of 958.400) came from the European Union, of which by far the biggest group came from Poland (199.600 people), followed by Germany (43.500 people), Romania (43.000 people), and Belgium (28.500 people) (CBS, 2020). In the first qualitative phase, we had contact with many organizations working with labour migrants such as municipalities, NGOs, and health care providers. They will be involved in recruitment of participants (see also 9.2, Recruitment and consent).

5. TREATMENT OF SUBJECTS

In this protocol we compare two arms. The treatment arm will receive our stepped-care program in addition to CAU. The comparison group will receive CAU alone. We will also provide PFA to all included study participants (i.e. in both arms). The CAU, PFA, and the stepped-care program are described below.

Psychological First Aid (PFA)

All participants, both in the treatment and the comparison group, will be offered individual Psychological First Aid (PFA) through telephone. This will be done before randomization. PFA is a WHO developed support strategy that involves humane, supportive and practical help for individuals living in a serious humanitarian crisis. PFA does not necessarily involve a discussion of the event(s) that cause the distress but aims particularly at five basic elements that are crucial to promote in the aftermath of crises, i.e. a sense of safety, calm, self- and community efficacy, connectedness, and hope (Hobfoll et al., 2007). PFA consists of a conversation (approximately 30-45 minutes) that a helper has with a participant which can be provided remotely. PFA has various themes; in PFA, the helper provides non-intruding practical care and support, listen to needs and concerns, helps people to address basic needs (e.g. information), listens to people without pressuring them to talk, comforts people and helps them to feel calm, helps people to connect to information, services, and social support, and protects people from further harm (WHO, 2011).

Care-as-usual (CAU)

In addition to PFA, both the treatment and the comparison group will receive care-as-usual (CAU); they will be allowed to receive any usual care. CAU ranges from community care to specialised psychological treatments, but only care provided by the general practitioner or the assistant of the general practitioner with a specialisation in mental health care (POH-GGZ) is fully covered by the basic health insurance package; it does not completely cover mental health service costs. Labour migrants who signed up for the basic health insurance package (which is obligated for everyone who plans to stay for more than 4 months or who needs to pay wage taxes due to work conducted in the Netherlands) have access to the health system like any other Dutch citizen with a standard insurance package.

Because labour migrants may have a lack of knowledge of the Dutch health care/health insurance system and therefore may not utilise health care (Aanjaagteam Bescherming Arbeidsmigranten, 2020), we will inform them about the Dutch health care/health insurance system as well. Additionally, participants will receive information about locally available referral options. We will

record for all participants whether they have a general practitioner (when they consent to having this recorded), or whether their employer offers access to a general practitioner. In case the participant gives approval, we will record name and contact details.

5.1 Investigational product/treatment

Treatment group: Stepped-care- Doing What Matters/Problem Management Plus (DWM/PM+)
Stepped-care models assume this approach provide health care in the most efficient and cost-effective way: the first step of care is readily available for all those in need and more costly or time intensive treatments are reserved only for those who do not respond. Evidence suggest that stepped-care models are modestly effective (van Straten, Hill, Richards & Cuijpers, 2014; Ho, Yeung, Ng & Chan, 2016) although there is a high heterogeneity in such models (number of steps, duration of steps, rules about stepping up) and their effects. Interestingly, research in clinical practice has shown that results improve when care providers switch from a matched care (i.e. matching treatment to patient characteristics like severity of problems), to a stepped-care approach (Boyd, Baker & Reilly, 2019).

The treatment group will receive the stepped-care program consisting of Doing What Matters (DWM) (step 1) and Problem Management Plus (PM+) (step 2), in addition to Psychological First Aid (PFA) and care-as-usual (CAU) (for details of CAU, see: '*Care-as-usual (CAU)'* below). Step 2 will only be provided if the participant still has elevated levels of psychological distress (K10 > 15.9) at week 6, i.e. during the second quantitative assessment at about 1 week after DWM. This is decided based on participants' score on the K10 and assessed by the researchers. If distress is still present (K10 > 15.9), participants in the treatment group are also offered 5 PM+ conversations with a helper. If the symptoms of distress have improved, they are not offered 5 PM+ conversations with a helper. Participants in the treatment group have no say in whether or not they are being offered Step 2 (PM+) as this is fully based on their symptoms of psychological distress as measured by K10.

Step 1: Doing What Matters (DWM)
Following the development of SH+, WHO conducted unpublished field tests providing the SH+ illustrated guide (DWM) as a guided self-help individually delivered intervention. SH+ and the DWM guide were designed to be relevant for large segments of adversity-affected populations: are intended to be transdiagnostic, easily adaptable to different cultures and languages, and both meaningful and safe for people with and without mental disorders. SH+ and DWM is based on acceptance and commitment therapy (ACT), a form of cognitive-behavioural therapy, with distinct

features (Hayes, Levin, Plumb-Vilardaga, Villatte & Pistorello, 2013). ACT is based on the concept that ongoing attempts to suppress unwanted thoughts and feelings can make these problems worse, so instead it emphasises on learning new ways to accommodate these thoughts and feelings without letting them dominate. ACT has been shown to be useful for a range of mental health issues (Tjak et al., 2015) and has been used successfully in a guided self-help format (Hayes et al., 2013).

The original DWM program consists of a self-help guide called *'Doing What Matters in Times of Stress'*, and pre-recorded audio exercises to support practice of skills such as grounding (a form of stress management). the guide is freely available as a PDF on the WHO website and can be read alone, or provided as a form of guided self-help by a briefly trained helper.

DWM includes five sections (or modules), each of which focuses on a specific skill. Participants receive information on thoughts and feelings they may experience and learn techniques on how to cope with them:

- **Section 1:** *Grounding:* Bringing attention back to the present moment when caught up in distressing emotions. In this section, it is explained what stress is, how it can affect the body, and how stressful situations, difficult thoughts and feelings can hook a person and may pull them away from their values. To deal with such emotions, participants learn to ground themselves, e.g. through breathing exercises and exercises to focus on the present.
- **Section 2:** *Unhooking:* Noticing difficult thoughts and feelings, naming difficult thoughts and feelings, and refocusing on what you are doing. In this section, it is further explained how being hooked by difficult thoughts and feelings makes a person move away from their values. Participants learn how to deal with this effectively, namely by accepting difficult thoughts and feelings instead of fighting them. They practice this by noticing and naming these thoughts and feelings and then refocus on what they are doing.
- **Section 3:** *Acting on your values:* Identifying personal values and then taking small or big actions to live in line with these values. In this section, participants learn and think about values that are important to them and how to put these values into action. By creating an action plan for the next week, participants will learn to act on their values. They also learn how to act when they are in a difficult situation that is not in line with their values.
- **Section 4:** *Being kind:* Enhancing and encouraging kindness towards oneself and towards others. In this section, participants learn that unkind thoughts are natural, but unhelpful. They learn how unkind thoughts pulls them away from their values and how they can speak kindly to themselves and others.
- **Section 5:** *Making room:* Learning how to tolerate stress while still acting consistently with values. In this section, participants learn how thoughts and feelings can change, how they

can make room for difficult thoughts and feelings without being hurt by them and continue to engage with the world around them. Additionally, they practice being kind to themselves.

In this study, the DWM program will be delivered as an online intervention. The DWM intervention, i.e. both the audios and the self-help guide, will be adapted for use on a smartphone or other device with internet access during Phase 1 of RESPOND. The format of DWM is innovative in that it seeks to ensure that key intervention components are delivered as intended through the use of a self-help guide, with more limited training and supervision as compared to other interventions. In the online version, a new module (i.e. section) will be released every week so participants will be asked to complete the entire DWM intervention within 5 weeks with weekly guidance from a helper (who is supervised by a registered (clinical) psychologist) through either a telephone call or an in-app messaging system. Due to its format, the DWM program requires reduced support from experts supporting implementation. The delivery mode for the support from the helper will be flexible and in line with COVID-19 regulations. Additionally, research has found that guided self-help programs produce much better results than "pure" (unguided) self-help, and the effects produced by guided self-help are surprisingly similar to face-to-face psychological treatment (Fledderus, Bohlmeijer, Pieterse & Schreurs, 2012).

Protocol adherence

We will assess DWM protocol adherence at post-intervention based on meta-data collected during the intervention (i.e. by tracking participant's usage of the DWM app such as who accesses what page, how often, how much time participants spend on the app etc.). We will use this meta-data during the intervention to remind each participant (e.g. through e-mail) after finishing each module to let them know that they should start a new module. Additionally, participants receive a weekly phone call from a helper to see how they are doing and to check on their progress. Helpers can see participants' progress in the app and answers given to questions in the app, as this information will help them support participants during the weekly phone calls. Helpers will receive regular supervision provided by the DWM trainers/supervisors. In addition to these, audio records of the sessions will be used for fidelity checks. The sessions will be audio recorded with professional equipment only if the participant gives consent to be recorded.

Step 2: Problem Management Plus (PM+)

PM+ is a brief, psychological intervention program based on cognitive behavioural therapy (CBT) techniques that are empirically supported and formally recommended by the WHO (Dua et al., 2011). The full protocol was developed by WHO and University of New South Wales, Australia. The manual involves the following empirically supported elements: problem solving plus stress

management, behavioural activation, facing fears, and accessing social support. Figure 2 shows a brief outline of the five sessions. In these 60-minute sessions participants may talk to trained non-professional helpers (who are supervised by registered (clinical) psychologists). We will follow this outline, except that we excluded all assessment instruments from the manual, since they are administered at the assessment phases instead of in the intervention sessions. PM+ has four core features: it is brief (five sessions); delivered by non-specialist helpers; transdiagnostic thereby addressing depression, anxiety, PTSD, stress and problems as defined by people themselves; and originally designed for people in low-income country communities but easily adaptable to different (vulnerable) populations, cultures and languages.

In this study, the delivery mode of the PM+ intervention will be flexible, with remote delivery in phases of the pandemic when physical distancing rules apply. In Phase 1 of RESPOND the PM+ intervention will be culturally and contextually adapted. This is a future-oriented attempt towards a more holistic mental health care system that can flexibly switch between modes of delivery (e.g. remotely through Zoom or face-to-face), depending on the needs and the specific containment measures that apply, and the specific preferences and needs of the participant.

Protocol adherence

Protocol adherence will be ensured by weekly supervisions provided by the PM+ trainers/supervisors as well as the fidelity checklist (Dawson et al., 2016). In addition to these, video records of the sessions will be used for fidelity checks. The sessions will be video recorded with professional equipment only if the participant gives consent to be recorded.

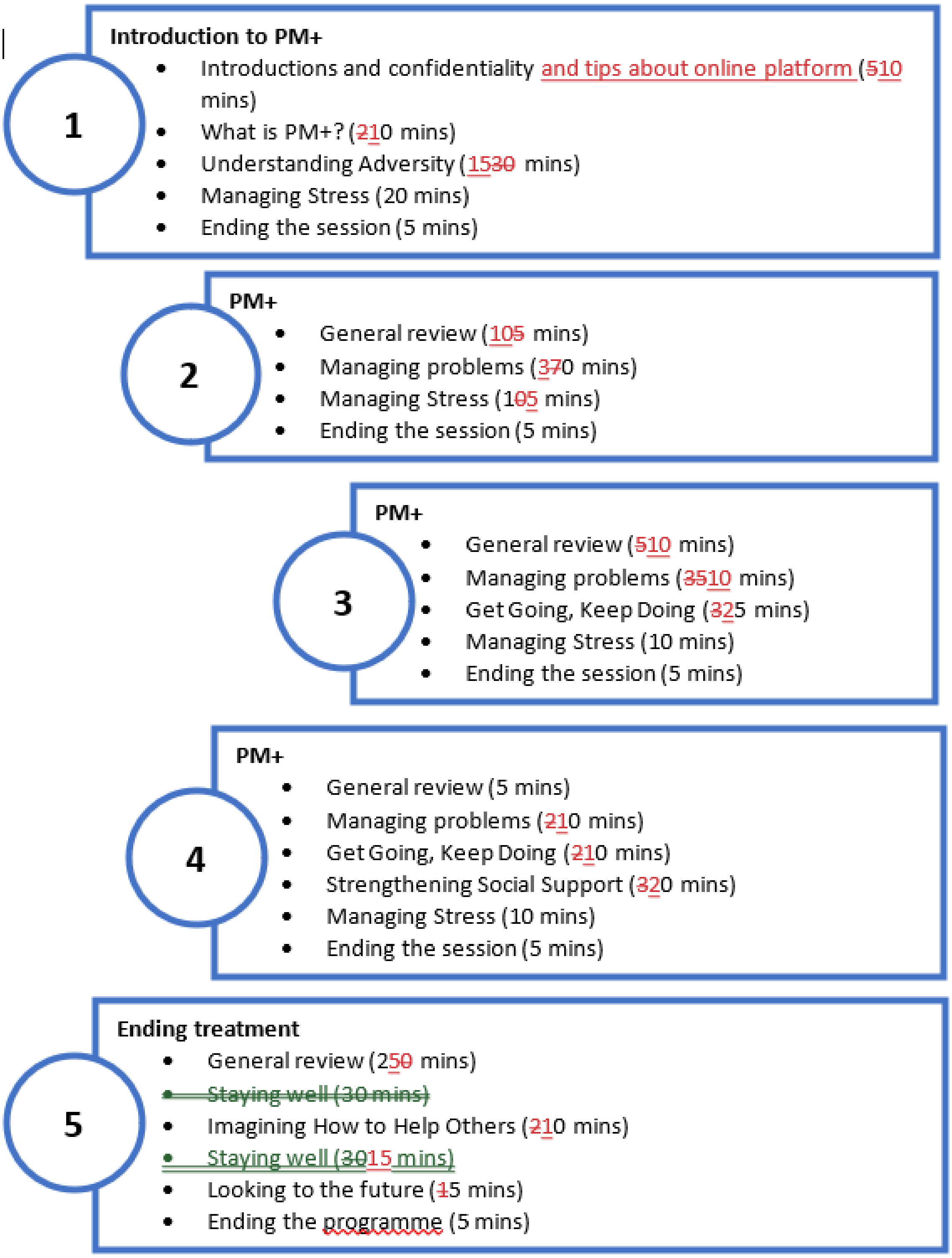

Figure 2. Outline of five PM+ sessions (figure from PM+ Manual, WHO, 2016, adapted to 60 minutes). We will follow this outline, apart from the PSYCHLOPS assessment which is excluded.

Helpers in PFA, DWM and PM+ interventions

A core feature of all WHO scalable interventions is that they can be trained to and delivered by non-professional helpers, such as a trained peer, or helper at the workplace, or a psychosocial worker (task-shifting) (WHO, 2017; Epping-Jordan et al., 2016). PFA, DWM, and PM+ are delivered by such non-specialist helpers. In general, non-specialist helpers selected to provide support to users should have at least finished secondary education (or received education until age 16). Additionally, for PM+ good knowledge and skills in providing psychosocial support is important. Delivering PFA, DWM or PM+ can be learned by both professionals and non-professionals who are in a position to help people impacted by distressing events or adversity. Helpers should speak the same language as the participant (as well as being able to communicate in either English or Dutch). It is not necessary for helpers to have a psychosocial or mental health background in order to be able to offer these interventions. Necessary skills include foundational helping skills, such as effective community and rapport-building skills, and experience supporting people in distress. In PFA, DWM and PM+ support may be provided remotely, it is therefore preferable that helpers have experience with remote support and receive training in this approach (e.g., EQUIP REMOTE). Practice sessions (e.g., providing support to other helpers, practice supporting persons with impairments) can help identify issues that may arise prior to working with users and build helper capacity. Helpers need to sign a confidentiality agreement and need to show a certificate of conduct. Helpers may deliver PFA, DWM and/or PM+, if they are trained (and supervised) in each intervention delivered.

Tasks of helpers

The helpers will deliver PFA, DWM, and/or PM+. PFA consists of a 30-45 minute phone call. In DWM, the helper gives a weekly, 15 minute phone call or may provide support via in-app messages. In PM+, the helper gives a weekly, 60-minute session. Helpers make appointments for these calls/sessions themselves. In addition to delivering the intervention, helpers will attend weekly supervision. Furthermore, we make a distinction between helpers delivering PFA, and helpers delivering DWM/PM+. Helpers delivering PFA may also work as a research assistant on this project and for example help with the recruitment or screening of participants. This difference between PFA-helpers and DWM/PM+-helpers is also reflected in our recruitment strategy.

Recruitment of helpers

One of the requirements for helpers is that they speak the same language as participants. In this study, we target labour migrants. As the majority of labour migrants in the Netherlands is Polish, we will mainly recruit Polish speaking helpers.

<u>PFA:</u> The helpers delivering PFA will be Polish speaking students or recent graduates, so they can also work as a research assistant. They will be recruited through university, recruitment websites,

LinkedIn, and/or (social) media (student) pages. We believe this is feasible, as we also hired Polish speaking students in the first qualitative phase of this study and experienced that there are enough Polish speaking students in the Netherlands.

DWM/PM+: The helpers delivering DWM/PM+ will be recruited through our network of Polish organizations based in the Netherlands, such as help organizations, mental health organizations. In the first qualitative phase of this research, we reached out to many Polish organizations. Several organizations stated that some of their volunteers/employees may be interested in being a helper. Additionally, we may post vacancies on recruitment websites, LinkedIn, and/or (social) media pages.

Training PFA helpers

Before providing PFA, helpers need training to enhance knowledge and gain better understanding of appropriate psychosocial responses and skills in providing support to individuals exposed to adversity (Sijbrandij et al., 2020). This half- or one-day (group-) training (WHO, 2013) includes explanations of the basic concepts and PFA principles, how to support (very) distressed people, and how not to cause further harm by using participatory learning (i.e. role-play). Due to COVID-19 restrictions, this training may be delivered remotely.

Training DWM helpers

Similar to PFA-helpers, the role of the coach in DWM is to provide brief motivational support to the participants; not provide specialized mental health services. Helpers should be empathetic and motivated to do this. Before working as a helper, helpers will receive a short training (2 or 3 days depending on whether they are already trained in PFA) by academics and/or mental health-care professionals. Helpers will be trained in providing support using the stress management guide (i.e., practice using the stress management in role-plays with other helpers) and practice to deliver support remotely (i.e. practice providing support in role-play settings). Helpers will receive a written manual as a guide for the brief support sessions. Prior to delivery of support to participants, helpers will undergo practice sessions and will receive ongoing support and supervision. Due to COVID-19 restrictions, this training may be delivered remotely.

Training PM+ helpers

PM+ helpers will receive eight days of training, followed by three practice cases, on-the-job training, and close supervision during the whole trial by the PM+ trainers/supervisors. The training program comprises of education about common mental disorders, basic counseling skills, delivery of intervention strategies and self-care (Rahman et al., 2016a). PM+ helpers will also need to complete

the EQUIP REMOTE ‘Delivering Remote Services’ training. Due to COVID-19 restrictions, this training may be delivered remotely.

Trainers/supervisors

All DWM/PM+ helpers will be actively trained and supervised by registered (clinical) psychologists and will be continued to be monitored throughout the process. Research assistants will independently assess and monitor treatment sessions at-random in order to ensure treatment adherence and fidelity in DWM and PM+. If needed, supervisors will be informed to give feedback to helpers. Furthermore, these expert clinicians will supervise the entire assessment and therapeutic process to reduce the burden on and risks for participants.

DWM/PM+ trainers/supervisors will consist of approximately five licensed mental health care professionals such as health-care psychologists or psychiatrists. They will be trained by a Master Trainer via a training-of-trainers (TOT) program, consisting of the same elements as the training for helpers, but also of training and supervision skills (Rahman et al., 2016a). The DWM/PM+ trainers/supervisors will be responsible for close supervision of the DWM/PM+ helpers. Therefore, as a next step, they will train DWM/PM+ helpers. Figure 3 shows how the TOT of DWM/PM+ is planned. Such training with continued supervision by professionals has been successfully used for non-specialist helpers (Murray et al., 2011).

The research team at Vrije Universiteit Amsterdam will be responsible for arranging the trainings and supervision for the lay helpers. Supervision of the helpers by the trainers/supervisors will take place on a weekly basis (Dawson et al., 2016). This will be done remotely or face-to-face, depending on the preference of the trainers/supervisors and accounting for COVID-19 regulations. The trainers/supervisors will also receive supervision by the Master trainer when necessary.

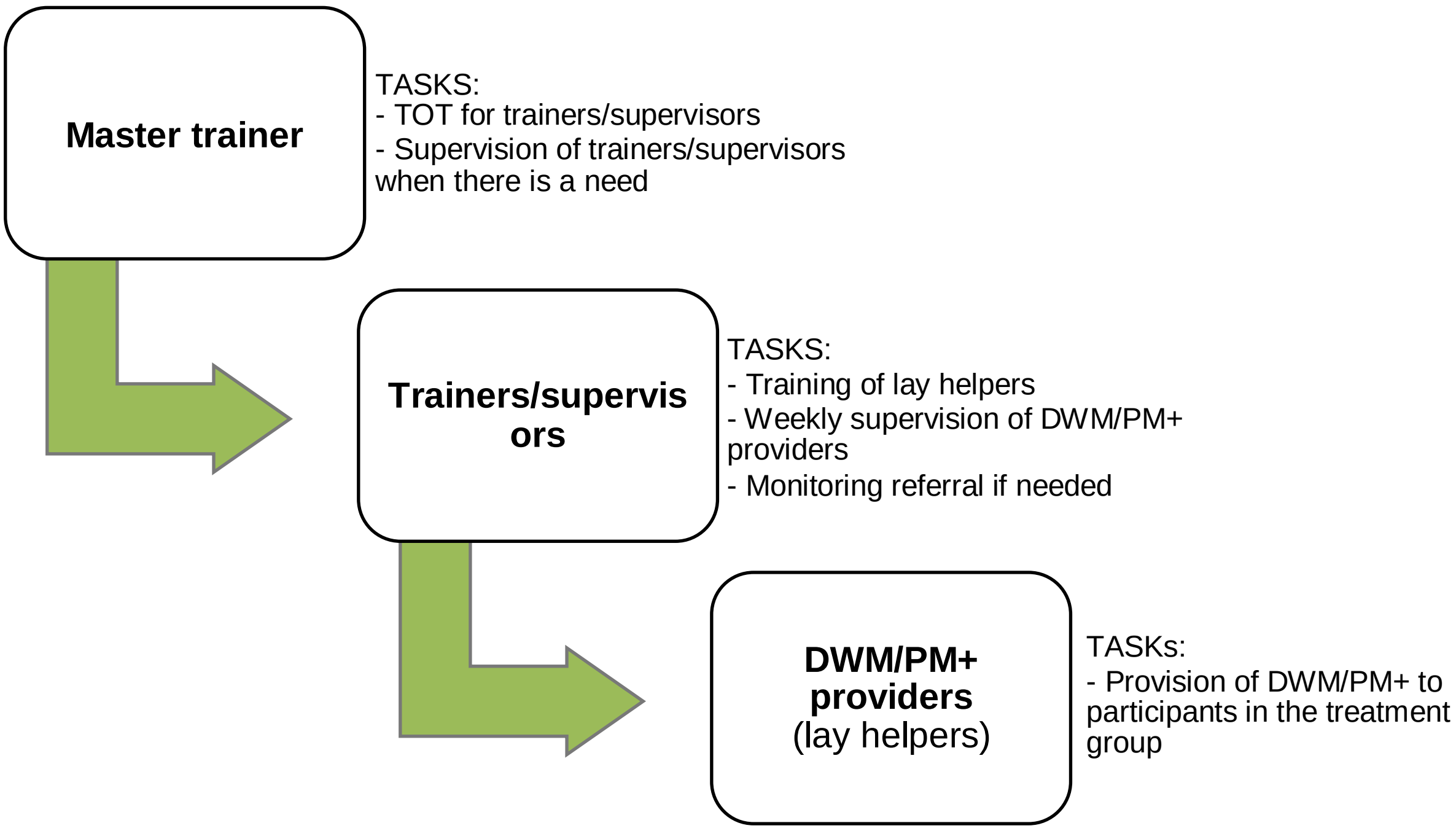

Figure 3. Training of Trainers/Supervisors and DWM/PM+ Helpers

5.2 Use of co-intervention (if applicable)

Not applicable.

5.3 Escape medication (if applicable)

Not applicable.

6. METHODS

6.1 Study parameters/endpoints

All participants will have five assessments (T0-T4), mostly by filling out online questionnaires. The five assessments take place (within a 14 day range) at the following time-points (see also Figure 1):

T0: Screening for eligibility including psychological distress

T1: Baseline assessment (week 0)

T2: 1 week post DWM intervention assessment (week 6)

T3: 1-week post PM+ intervention assessment (week 12)

T4: 2-months post PM+ intervention assessment (week 20)

The hypothesis to be tested is that the stepped-care program consisting of Doing What Matters (DWM) (step 1) and Problem Management Plus (PM+) (step 2) plus Psychological First Aid (PFA) and care-as-usual (CAU) will be more effective in decreasing psychological distress compared to PFA and CAU alone.

6.1.1 Main study parameter/endpoint

The main study parameter will be the decrease in symptoms of depression (and change in caseness) from baseline to two-month follow-up after the PM+ intervention ended, measured through the combined sum score of the Patient Health Questionnaire-9 (PHQ-9) and Generalized Anxiety Disorder (GAD-7) previously validated as the PHQ-ADS (Kroenke et al., 2016; 2019). A description of the measure(s) can be found under 'Study Procedures'. Based on prior studies on PM+ in Pakistan and Kenya (Rahman et al., 2016b; Bryant et al., 2017) where PM+ was administered as a standard treatment, we expect to detect a Cohen's d effect size of 0.3 in the PM+ group at 2 months post-treatment.

6.1.2 Secondary study parameters/endpoints (if applicable)

1. Level of depression (PHQ-9)
2. Level of anxiety (GAD-7)
3. Severity of posttraumatic stress disorder (PCL-5)

4. Resilience based on exposure to stressful (general- and COVID-19 related-) events (MIMIS)
5. Positive appraisal style (PASSc)
6. Quality of life (EQ-5D-5L)

7. Cost of care: impact on use of health system, other services, time out of employment and other usual activities and need for informal care (CSRI schedule)

The measurement instruments are described under ‘Study procedures’.

6.1.3 Other study parameters (if applicable)

The measurement instruments are described under ‘Study procedures’.

1. Demographic data
2. Impact of COVID-19 questionnaire
3. Exposure to adverse life-events (BTQ)
4. Hair samples (cortisol, cortisone, dehydroepiandrosterone (sulfate) (DHEA(S)) , progesterone and testosterone concentrations)
5. Digital markers (facial activity, movement, voice)6. . Treatment fidelity (DWM: tracking app usage based on meta-data, audio records, checklists, PM+: video records, checklists)
7. Satisfaction (qualitative assessment in study phase 3)
8. Acceptability of the program (qualitative assessment in study phase 3)
9. Implementation indicators: reach, dose, resource use, costs of recruiting, training and retaining staff delivering the stepped-care program, program costs, adaptation, the process, quality

6.2 Randomisation, blinding and treatment allocation

After the screening and the baseline assessment, participants will be randomised to either the treatment group (n=106) or the comparison group (n=106) , with an equal probability of assignment to each group (allocation ratio 1:1). The trial is a single-blind RCT (i.e. outcome assessors are blind to treatment allocation). Randomisation will be carried out through computerised software (e.g. Castor) using limited block size (e.g. 6-12 participants per block) and will be performed by an independent person who is not involved in the assessment. If participants are randomised into the treatment group, they will be allocated to a DWM helper and given access to the DWM online app. This is also done blind by an independent person who is not involved in the assessment. The allocated helper will contact the participant through a method agreed when informing the participant about the result of randomization (e.g. telephone, message or email) to set up a time and date for a first acquaintance telephone call before the intervention starts. During this call, the weekly support calls of the DWM helper will be discussed and planned. If participants still meet the inclusion criteria 1 week after the DWM program ended, they will be contacted by the PM+ helper (might be the

same helper as for DWM) to plan five consecutive (tele-conferencing) meetings of 60 minutes with the participant. The first session will be scheduled within a few days and no longer than one week after the pre-intervention assessment.

6.3 Study procedures

Study phase 1

Study phase 1 is described in a separate protocol for which ethical approval has been granted by the VCWE. Study phase 1 is a qualitative study with key stakeholders to culturally and contextually adapt the DWM and PM+ manuals. This study also informs the second study phase as to how the DWM/PM+ intervention can best be implemented.

Study phase 2

Study phase 2 will be carried out at the community level in the Netherlands. Eligible participants will be adult labour migrants (18 years or above) with elevated levels of psychological distress but without acute medical or severe mental health conditions (see 4.2 and 4.3 for inclusion and exclusion criteria). The recruitment and informed consent procedure will be further explained under *9.2 'Recruitment and consent'*.

Screening (T0)

Following informed consent (), participants will be invited to complete step 1 of the first assessment: screening. Screening consists of using a measurement instrument and other questions to see if people meet the inclusion criteria (6.2 Inclusion criteria). Also, specific questions are asked to check whether participants should be excluded because of fulfilling exclusion criteria.

- *Screening for inclusion criteria*

  In order to be included in the study, participants need to score above 15.9 on the Kessler Psychological Distress Scale (K10), a 10-item screening questionnaire for common mental disorders (Kessler et al., 2002). This instrument will be self-administered and in this way literacy (as inclusion criterion) is immediately accounted for. Because DWM consists of an app with text, it is important that people are able to read (or if visually impaired use a screen reader, i.e. software that converts text to speech). If indicated, they will be referred to external specialists for potential follow-up. More detailed explanations of all measures are described under 'Measurement Instruments'.

- *Screening for exclusion criteria*

If individuals meet inclusion criteria, they will be screened for exclusion criteria (6.3 Exclusion criteria). If they meet one or more exclusion criteria they will be excluded and referred for appropriate treatment and support. We will assess whether individuals: (a) plan to move abroad within the next 6 months; (b) have plans to end their life or are in acute need of protection (see Measurement Instruments); c) currently use psychotropic medication of which the dose has been changed in the past 2 months or currently receive specialised psychological treatment and d) have a severe mental disorder (e.g., psychotic disorders, substance-dependence) or severe cognitive impairment (e.g., severe intellectual disability or dementia) (see Measurement Instruments for questions/observations in either a face-to-face, telephone or tele-conferencing contact during screening) (WHO, 2016, pp. 87).

- When participants are not selected for the trial because they score below the cut-off scores for the K10 or when they meet the exclusion criteria as described in section 6.3 Exclusion Criteria, they will immediately be provided feedback e.g. on the screening outcomes (including K10 score) and an explanation why they are not eligible for the study (p. 86 of the PM+ intervention manual; WHO, 2016). When participants are excluded because of an imminent suicide risk, expressed acute needs/protection risks (for example, a young woman who expresses that she is at acute risk of being assaulted or killed) or observed (suspicion of) severe mental disorders or severe cognitive impairment they will be referred for appropriate treatment and support (such as their general practitioner, an external mental health specialised support (e.g. GGZ Keizersgracht for Polish labour migrants) or to local social service provision, depending upon their needs. If the patients agree to be referred, the assessment results will be provided to their general practitioner or the treating mental health professional with permission of the participant.

Baseline assessment (T1)

If participants meet the eligibility criteria and score above the cut-off of the K10, they will continue with the baseline assessment. This step includes questions about socio-demographic characteristics and; the Brief Trauma Questionnaire (BTQ); PTSD Checklist for DSM-5 (PCL-5); the Patient Health Questionnaire-9 (PHQ-9), General Anxiety Disorders-7 (GAD-7), COVID-19 related questions, the Mainz Inventory of MIcrostressorS (MIMIS), Checklist for Quality of life (EQ-5D-5L), Positive Appraisal Style Scale – content focused (PASSc), the Client Service Receipt Inventory (CSRI), and hair cortisol, cortisone, DHEA(S), progesterone and testosterone concentrations.

Participants who do not meet the eligibility criteria and score below the cut-off of the K10 (<15.9), but who did give written permission to give hair samples, will be asked if they are still willing to do this despite not being able to be included in the rest of the study. If participants are still willing to give a hair sample, they will do so only at baseline and also fill in the T1 baseline assessment.

Informing about the result of randomization

After T1, a researcher will call the participant for PFA. At the end of the phone call, the researcher will randomize the participant and inform the participant about the result. If the participant is randomized in the intervention group, preferences for remote contact (e.g. video-conferencing, e-mail/telephone) will be registered.

Post-intervention and follow-up assessment (T2, T3, T4)

Quantitative assessments will take place four times for all participants: at screening (T0) and baseline (T1: before the intervention, week 0), at 1 week after DWM (T2, week 6) and at 1 week (T3, week 12) and at 2 months (week 20) after the PM+ program has finished (T4). In practice, assessments may be up to 14 days after the intended assessment time, e.g. due to rescheduled sessions. All instruments used in the baseline assessment (T1) will be used for each of the post-intervention and follow-up assessments, except for the BTQ, which will only be re-administered at T4..Sociodemographic questions are also asked at T2, T3, and T4. The screening instrument K10 (T0) will be re-assessed at T2 only. Furthermore, at T2, T3, and T4, the Step-by-Step suicide screening question will be administered. See Table 1 for an overview. In case participants do not respond to a scheduled assessment, they receive reminders per e-mail and are called a maximum of five times (on different days) for scheduling a new appointment.

If a participant gave permission to give hair samples ánd screened in for the study, hair samples will be taken again at T4 for the analysis of hair cortisol, cortisone, DHEAS, progesterone, and testosterone concentrations.

Assessors

Screening for inclusion criteria and assessments T1 to T4 will be conducted online (collective program CASTOR EDC).

Additionally, assessment of exclusion criteria, such as suicide risk, mental, neurological or substance use disorders,

will be conducted by an assessor, in person or through video/telephone calls.

Assessment during intervention: treatment fidelity and digital markers

DWM: Participants' usage of the DWM app will be tracked, such as who accesses what page, how often, how much time participants spend on the app, etc. This way, we can track protocol adherence to the DWM app afterwards, once they finished the intervention.. Additionally, participants will receive a weekly phone call from a helper to see how they are doing and check on their progress. Limited metadata of participants' tracked app usage behavior will be shared with the DWM helpers; Helpers will be able to see participants' answers given to questions in the app and their progress, so they can give better support to participants.

DWM and PM+: Audio tapes of the DWM phone calls and video tapes of the PM+ treatment sessions will be recorded to monitor treatment fidelity by VU Amsterdam research assistants.

Digital markers: Due to the program used for the PM+ sessions, it is not possible to only make audio records of PM+ sessions; when video is used, only video records can be made. For this reason, we will ask participants if we can use these video records for further analysis of digital markers.

Measurement instruments

The measurement instruments that will be used for the four quantitative assessments, i.e. at screening (T0) and baseline (T1), post-intervention 1 (T2), and post-intervention 2 (T3), and follow-up (T4), as well as during the DWM/PM+ intervention are depicted in Table 1. In case there is no translation of a measurement instrument in Dutch or in the native language of the target population, the instrument will be translated and back-translated by the research team. All instruments (as well as the intervention) will be administered in the native language of the participant, or in English or Dutch.

[PLEASE FIND TABLE 1 below with Overview of the concepts, their measures, the type of study parameter in the study, and the moment of measuring during study phase 2 and 3]

Table 1. Overview of the concepts, their measures, the type of study parameter in the study, and the moment of measuring during study phase 2 and 3

Concept	Measures	Type of study parameter	Moment of measuring							
			Phase 2							
			Screening (T0)	Baseline (T1)	DWM	Post-assessment 1 (T2)	PM+	Post-assessment 2 (T3)	Follow-up assessment (T4)	P
Psychological distress	K10	Screener	x			x				
Suicide risk:										
- Face-to-face *or*	PM+ tool	Screener	x							
- Self-administered	Step-by-step	Screener	x			x		x	x	
Mental, neurological or substance use disorders	PM+ tool	Screener	x							
Depression and Anxiety:	PHQ-ADS	Primary								
Subscale depression	PHQ-9	Secondary		x		x		x	x	
Subscale anxiety	GAD-7	Secondary		x		x		x	x	
Posttraumatic stress reactions	PCL-5	Secondary		x		x		x	x	
Resilience	MIMIS	Secondary		x		x		x	x	
Resilience factors	PASSc	Secondary		x		x		x	x	
Quality of life	EQ-5D-5L	Secondary		x		x		x	x	
Impact on resource use/costs	CSRI	Secondary		x		x		x	x	
Socio-demographics		Other	x	x		x		x	x	
Impact of COVID-19	COVID-19 questionnaire	Other		x		x		x	x	
Exposure to life events	Brief BTQ	Other		x					x	
Hair sample parameters	Hair sample + questionnaire	Other		x					x	
Digital markers	Video records						x			

Treatment fidelity:<br>- DWM	Metadata, audio records	Other	x	
- PM+	video records	Other		x
Satisfaction DWM	Interview	Other		
Satisfaction PM+	Interview	Other		

Screeners

Screening instruments

K10: psychological distress

Psychological distress will be measured using the Kessler-10 Psychological Distress Scale (Kessler et al., 2002). The K10 is a ten-item self-report questionnaire to screen broadly for psychological distress (e.g. anxiety and depression related distress) experienced in the past 30 days. Items are rated on a five-point Likert scale ranging from *none of the time* to *all of the time*. The sum of the ten items gives a total score ranging from 10 to 50. Higher scores represent higher levels of distress. The K10 has strong psychometric properties and has strong discriminatory power to distinguish DSM-IV cases from non-cases (Kessler et al., 2002). The K10 has been validated in various population samples and is a useful instrument in both primary care (Kessler et al., 2002) and general population samples (Furukawa, Kessler, Slade & Andrews, 2003; Kessler et al., 2005). Moreover, the K10 has been found to not have any substantial bias in regards to education level and gender, thus making it useful for research (Baillie, 2005). The K10 exists in various languages, among which are English, Dutch, and Polish. To our knowledge, the Polish version of the K10 has not been validated, but the Dutch version has been validated among adults from the general population. The Dutch version was found to have good psychometric properties with an optimal cut-off score of 20 to detect any depressive and/or anxiety disorder (Donker et al., 2010).

There is no standard cut-off score for the K10 at present. In addition to a cut-off score of 20, lower cut-off scores have also been found, e.g. a cut-off score of 12 (Lace et al., 2019) or a cut-off score of 14 (Baggaley et al., 2007). When determining the appropriate cut-off point, it is important to take into account the context in which the measurement instrument is used. In order to not miss potential participants, in research a low cut-off score with a low rate of false negatives and a high sensitivity is favored (Smits, Smit, Cuijpers & De Graaf, 2007). In STRENGHTS, a similar study to the RESPOND project, among Syrian refugees in the Netherlands, a cut-off point of 15 was used to indicate moderate to high levels of psychological distress (de Graaff et al., 2020). This was based on a study among Afghan and Kurdish refugees asylum seekers in New Zealand and Australia where they used the following cut-off scores: 10–15.9 (low risk of psychological distress), 16–21.9 (moderate levels of distress consistent with a diagnosis of moderate depression and/or anxiety disorder), 22–29.9 (high level of distress) and 30 or more (possibility of very high or severe levels of distress) (Sulaiman-Hill & Thompson, 2010). As the RESPOND project is conducted in various European countries with various (vulnerable) populations (e.g. health care workers

in Spain and refugees in Italy), we will use a cut-off score of 15.9 which we believe is appropriate for these varying target populations.

Screening instruments for exclusion criteria

Suicidal ideation

Suicidality will be explored at several time-points (at T0, and at follow-up assessments T2, T3, and T4) with either the 'assessment of thoughts of suicide' risk tool (from PM+; WHO, 2016, pp. 86) when assessed in face-to-face contact (e.g. in person or remotely through teleconferencing or telephone) or with the self-administered step-by-step suicidality question (Van 't Hof et al., 2021) when assessed with an online questionnaire. People who have plans to end their life (as indicated by an answer of "yes" on the screening question - "In the past week/month, have you had serious thoughts or a plan to end your life?" or when answered positive on similar questions with the PM+ tool: "In the past month, have you had serious thoughts or a plan to end your life?", "What actions have you taken to end your life?", and "Do you plan to end your life in the next two weeks?") will be excluded from the study as they will be considered to be at imminent risk of suicide.

If someone answers 'yes' to the online screening question, an on-screen message will appear explaining that they may need additional mental health support with advice to go to an emergency room or call the national suicide hotline. They will also be presented with suggestions for steps to follow in order to receive mental health care (e.g. contact general practitioner), encouraged to seek help, and provided with additional self-care tips. Additionally, researchers (PI and assessors) will receive a notification when a participant answers positive on this question. In that case, as well as when a participant answers positive on similar questions with the PM+ tool, there will be follow-up with the participant by reaching out to the participant and applying the Suicidal Scale of the Mini-International Neuropsychiatric Interview (MINI; Van Vliet & De Beurs, 2007). If the MINI raises suspicion of suicidality, with permission of the participant, researchers will contact the participants' general practitioner (GP) to make an appointment, or if the participant does not have a GP they will contact a GP near the participants' home. After 3 days, researchers will follow-up with the participant to see if (s)he has indeed visited the GP. Consent of the participant (or his/her representative) for referral is not needed in certain (rare) situations where immediate referral is necessary due to a serious likelihood of immediate or imminent harm to the participant or to other persons.

Severe mental disorder

(Suspicion of) a severe mental disorder will be assessed by the PM+ tool 'Impairments possibly due to severe mental, neurological or substance use disorders'. This is a tool which is to be filled in by assessors based on their observations and judgment of clients'

behaviours. No questions are asked to the participant. The tool asks 4 questions related to the participant's behaviour: 1) does the client understand you (even though they speak the same language or dialect)?; 2) Is the client able to follow what is happening in the assessment to a reasonable extent?; 3) Are the client's responses bizarre and/or highly unusual?; 4) From the client's responses and behaviours, does it appear that they are not in touch with reality or what is happening in the assessment? If the answer is no to question 1 or 2, or yes to question 3 or 4, the participant will be excluded.

Primary outcome measure

The PHQ-ADS is the sum of the PHQ-9 and GAD-7 scores (details of both instruments summarised below) and thus can range from 0 to 48, with higher scores indicating higher levels of depression and anxiety symptomatology. Cut points of 10, 20, and 30 on the PHQ-ADS indicate mild, moderate, and severe levels of depression/anxiety symptoms, respectively. Two validation studies of the PHQ-ADS in trial data-sets of patients with chronic (musculoskeletal) pain and oncological diseases have been published (Kroenke et al., 2016; Kroenke et al., 2019). Evidence shows high internal reliability (Cronbach's alpha of 0.8 to 0.9), strong convergent and construct validity, sufficient uni-dimensionality and evidence for sensitivity to change, differentiating between individuals classified as worse, stable, or improved by a reference measure at three months post-intervention.

Secondary outcome measures

PHQ-9: depression (PHQ-9; subscale of PHQ-ADS)

Depressive symptoms during the past two weeks will be measured using the Patient Health Questionnaire depressive module. It asks how often someone was bothered by each of the nine DSM-5 criteria and scores answers on a four-point Likert scale ranging from 0 (not at all) to 3 (nearly every day) (Kroenke, Spitzer, & Williams, 2001). In addition to the nine items, the PHQ-9 asks: "If you checked off *any* problems, how *difficult* have these problems made it for you to do your work, take care of things at home, or get along with other people?", which is to be answered with "Not difficult at all", "Somewhat difficult", "Very difficult", or "Extremely difficult". For the current study, we will examine changes in caseness in depression. We will use a cut-off score of 10, which has been found to be a valid cut-off point for diagnosis (Manea, Gilbody & McMillan, 2021). The PHQ-9 has been translated to and is available in many languages (see https://www.phqscreeners.com/), amongst which are English, Dutch, and Polish. The PHQ-9 has been found to be a reliable and valid instrument to measure depressive severity. Furthermore, due to its brevity, PHQ-9 is a useful instrument for usage in a clinical or research setting (Kroenke et al., 2001). The Polish and the Dutch version of

the PHQ-9 have previously been validated. Among the general population (aged 35-64), the Polish version has been found to be a valid tool to diagnose depression (Ślusarska et al., 2019). The Dutch version has been found to be a valid tool among elderly primary care patients with diabetes mellitus (DM) and chronic obstructive pulmonary disease (COPD) without known depression (Lamers et al., 2008). Moreover, a study on six different ethnic minority groups in the Netherlands on measurement invariance found that the PHQ-9 was measurement invariant as regards to interview mode (interview, paper, or internet) and language. Therefore, the PHQ-9 can be used in different settings to compare the level of symptoms of depression across ethnic groups (Galenkamp, Stronks, Snijder & Derks, 2017).

GAD-7: anxiety symptoms (GAD-7; subscale of PHQ-ADS)

The Generalized Anxiety Disorder (GAD-7) questionnaire is a seven-item, self-report anxiety questionnaire which assesses the degree to which the patient has been bothered by feeling nervous, anxious or on edge over the last two weeks. Items also include other generalised anxiety symptoms such as being unable to stop worrying about multiple things, having trouble relaxing or sitting still, feeling irritable and being afraid of something bad happening at all times (Spitzer et al., 2006). Items are scored from 0 to 3, respectively for experiencing symptoms 'not at all', for 'several days', for 'more than half the days' and for 'nearly every day'. The total score ranges from 0 to 21. Cut-off points for mild, moderate and severe anxiety, are scores of 5, 10 and 15, respectively (Spitzer et al., 2006). A score of 10 has been identified as the optimal cut-off score to balance specificity and sensitivity (Spitzer et al., 2006).

The GAD-7 has been translated to and is available in many languages (see https://www.phqscreeners.com/), amongst which are English, Dutch, and Polish. The GAD-7 has been found to be a valid and reliable instrument in both the psychiatric (Kertz, Bigda-Peyton & Bjorgvinsson, 2013; Rutter & Brown, 2017) and the general population (Löwe et al., 2008; Hinz et al., 2017), including the Dutch general population (Donker, van Straten, Marks & Cuijpers, 2011). Although the Polish version of the GAD-7 has not been validated, recently it was used in various studies to assess anxiety during the COVID-19 pandemic in Poland (Okruszek, Aniszewska-Stańczuk, Piejka, Wiśniewska & Żurek, 2020; Dragan, Grajewski & Shevlin, 2021; Gambin et al., 2021). In these studies in Poland, internal consistency of GAD-7 was found to be strong (α = .92 - Dragan et al., 2021; α = .94 - Gambin et al., 2021).

PCL-5: PTSD Symptoms

Posttraumatic stress disorder (PTSD) symptoms during the past week according to the DSM-5 PTSD diagnosis will be measured using the PTSD Checklist for DSM-5 (PCL-5) (Weathers et al., 2013). A shortened 8-item version of the original PCL-5 (a 20-item checklist which correspond with the 20 DSM-5 PTSD symptoms) will be used. Items are rated on a 0-4 scale. Added up, the maximum severity score of the 8-item version is 32. Higher scores indicate higher symptomatology. The original 20-item PCL-5 exists, amongst others, in English (Weathers et al., 2013), Dutch (Boeschoten, Bakker, Jongedijk & Olff, 2014) and Polish (Ogińska-Bulik, Juczyński, Lis-Turlejska, Merecz-Kot, 2018). The original PCL-5 has been found to have good reliability and validity in English (Blevins, Weathers, David, Witte & Domino, 2015). The Dutch and Polish versions of the PCL-5 have also been found to have good psychometric properties and can be used for both scientific and clinical purposes (Ogińska-Bulik et al., 2018); Van Praag, Fardzadeh, Covid, Maas & Steinbüchel, 2020), although the Dutch version was examined in a civilian population after experiencing traumatic brain injury.

Since populations in the trials might not all be severely traumatized, the abbreviated 8-item version will be used (Price et al., 2016). In a comparison of two abbreviated version, i.e., the 4-item and 8-item versions of the PCL-5, the PCL-5 8 item version showed a strong correlation with the total scale, greater internal consistency, and allowed for sufficient variability in patient response. Internal consistency of the 8-item version was α 0.90 (comparable to total PCL-5 20-item version) and accounted for 94.1% (R = 0.97) of the variance in the total PCL-5. There were no significant differences in the sensitivity and specificity between the total 20-item PCL-5 scale and the 8-item scale (Price et al., 2016).

MIMIS: resilience

The Mainz Inventory of Microstressors (MIMIS) was recently developed to measure objective microstressors of modern life in the past 7 days (Chmitorz et al., 2020). In the Dynacore-C study (Veer et al., 2021) this was changed into a period of 2 weeks. The MIMIS uses a definition of resilience as a trade-off between the outcome of mental health and exposure to adversity. Outcome-based resilience will be assessed by relating self-reported changes in mental health problems (i.e. anxiety and depression) over the past 2 weeks (assessed with the PHQ-ADS) to the self-reported exposure to 11 categories of general stressors (life events and daily stressors such as physical health problems, family conflicts or separation form a loved one) and 29 COVID-19 crisis related stressors (such as COVID-19 symptoms, belonging to a risk group for serious COVID-19 symptoms, loss of social contact, or problems arranging childcare (Veer et al., 2021). For this study, the MIMIS has been shortened to 22 questions measuring life events and general and COVID-19 related

stressors to reduce burden on participants, and items were adapted to fit the target group. Items are rated on a five-point Likert scale in which participants can indicate to what extent the situation caused them mental strain, ranging from 0 (not at all straining) to 4 (very straining). e.g., family, friends, work, finances, environmental and living conditions). If participants did not experience the situation in the past 7 days (or two weeks similar to Dynacore-C study), they can choose ‘did not occur’. A high correlation has been found between the ecological momentary assessment (EMA), end-of-day, and end-of-week versions of the MIMIS regarding the occurrence and severity of microstressors, thereby indicating ecological validity of the MIMIS questionnaire (Chmitorz et al., 2020).

Resilience factor(s): positive appraisal style

Resilience factors (i.e. underlying factors that lead to resilience) may also be measured by assessing factors like optimism, positive appraisal style, perceived social support (in general and related to COVID-19), perceived self-efficacy and behavioral coping style. In RESPOND, we will assess positive appraisal style with the “Positive Appraisal Style Scale – content focused” (PASSc). The PASSc is based on positive appraisal style theory of resilience (PASTOR; Kalisch et al, 2015; Kalisch et al, 2021). The PASTOR theory conceptualises resilience as an outcome: the maintenance of mental health after stressor exposure. Positive appraisal style would therefore not be a measure of resilience, but a resilience factor. It intends to capture the underlying mechanism which leads to resilience. The PASSc is currently used in a number of longitudinal studies (Mainz Resilience Study (MARP); Longitudinal Resilience Assessment study (LORA)and several studies of the DynaMORE project). The PASSc was originally developed as a 29 items questionnaire featuring generalized positive appraisals of and attitudes towards difficulties, covering specifically the 3 main dimensions of stressor/threat appraisal - appraisal of threat magnitude/cost (relating to catastrophizing vs. trivialization), of threat probability (relating to pessimism vs. optimism), and of one’s coping potential (relating to helplessness vs. overconfidence). Internal validity testing and a factor analysis resulted in a reduced list of 12 items, which is the Positive Appraisal Style Scale (PASSc). A paper (R. Kalish and P. Petri-Ramao) currently being prepared shows internal consistency $\alpha = .87$ and reliability Cronbach’s $\alpha = .84$. The PASSc shows convergent validity with other underlying resilience factors as it correlates with optimism .52 (SOP-2), with stress recovery (BRS): .50, with well-being (WHO-5): .42, with trait anxiety (STAI-Y2): -.51 , with neuroticism (from BFI-10): -.49. Discriminant validity is shown in low correlation with I-8 impulsivity subscales urgency, intention <=.13; with openness (from BFI-10): .17, with conscientiousness (from BFI-10): .19.

EQ-5D-5L: quality of life

The EQ-5D-5L measures quality of life and consists of two parts, the EQ-5D and the EQ VAS. Part 1, the EQ-5D, rates the level of impairment across five dimensions: mobility, self-care, usual activities, pain/discomfort, and anxiety/depression. Each dimension has 5 levels: no problems, slight problems, moderate problems, severe problems and extreme problems. The EQ-5D-5L is an adapted version of the EQ-5D(-3L), which only had three response options for each dimensions and was therefore thought to not sufficiently capture milder health issues and small changes between different states of health (Herdman et al., 2011). The EQ-5D-5L has been used widely and is available in over 150 languages, also for laptop, tablet or Castor EDC (https://euroqol.org/eq-5d-instruments/eq-5d-5l-available-modes-of-administration/self-complete-for-use-in-castor-edc/). Country specific utility weights will be attached to data from the EQ-5D-5L and changes in participant quality of life years gained between intervention and control groups will be determined. Part 2, the EQ-VAS, is a visual, vertical, analogue scale. The endpoints of the scale are called ‘The best health you can imagine’ and ‘The worst health you can imagine’ and the current health status of that day needs to be indicated. EQ-5D-5L has been slightly adapted to be used in online screening.

CSRI schedule: cost of care

The Client Service Receipt Inventory (CSRI) was developed for the collection of data on service utilization (e.g. use of health system, other services, time out of employment and other usual activities, need for informal care) and related characteristics of people with mental disorders, as the basis for calculating the costs of care for mental health cost-effectiveness research. It has been used cross-culturally and is available for the Netherlands (Chisholm et al., 2000).CSRI always needs local adaption, as standard please include questions on use of general primary and secondary health care services as well as mental health services. The instrument will be further adapted to suit the context of labour migrants in the Netherlands.

Other measures

Socio-demographic information

Socio-demographic information will be collected with predefined items based on the REDEFINE and STRENGTHS studies (i.e. age, gender, nationality, years of education, relationship status, and main work-status and additional questions regarding country of birth, household population (incl. children < 18 and elderly people), household income on average, occupational area working, mental health condition and overall current health status and housing (square meters of the house, outdoor space available). Sociodemographic questions are asked during the screening at T0, to have an idea of who is excluded, and additionally during baseline assessment at T1. Furthermore, some questions are asked during follow-up

assessments during T2, T3, and T4, so we for example know if there are participants who did move abroad during their participation in the trial.

BTQ: exposure to potentially traumatic events

Previous stressor exposure will be assessed using a shortened version of the Brief Trauma Questionnaire (BTQ) (Schnurr, Vielhauer, & Weathers, 1995). (Information about the reliability and validity of the BTI is provided in Schnurr et al., 2002). The BTQ was originally designed to assess traumatic exposure according to DSM-IV but specifically asked only about Criterion A.1 (life threat/serious injury) because of the difficulty of accurately assessing A.2 (subjective response) in a brief self- report format. Criterion A.2 has been eliminated from the PTSD diagnostic criteria in DSM- 5, so the BTQ provides a complete assessment of Criterion A. The questionnaire may be used to determine whether an individual has had an event that meets the A Criterion, or to determine the different types of Criterion A events an individual has experienced. The BTQ consists of 10 questions measuring life events that may be particularly stressful or disturbing for most people. We will only measure which life events took place, not if someone thought their life was in danger or if they may be seriously injured, or if this was also the case. For assessment 4, the BTQ has been adapted for repeated usage in a study.

Impact of COVID-19 questionnaire

The COVID-19 questionnaire includes 11 questions related to the impact of COVID-19. The questionnaire is based on other COVID-19 questionnaires (Conway, Woodward & Zubrod, 2020). It may be backward translated from English to other languages and will be administered at each time-point.

Hair biomarkers:

Saliva or blood can be used to study short-term hormone fluctuations. However, hair can be used to study hormone release over longer time intervals, as it indicates the hormone secretation over the past months. Therefore, stress hormones that can ben found in the hair are good biomarkers to study physiological responses to chronic stressors. In hair A commonly used biomarker to study this physiological response are hair cortisol concentrations (HCC). (Buchmüller et al., 2020; Russell et al., 2012; Stalder et al., 2017; Steudte-Schmiedgen et al., 2016; Noppe et al., 2015).

In addition to HCC, cortisone levels, dehydroepiandrosterone (sulfate) (DHEA(S)), progesterone, and testosterone concentrations can also be measured. Cortisol can be changed into (inactive) cortisone. Salivary cortisone levels have been found to closely follow cortisol levels, but in hair, cortisone levels are about three to four times higher than cortisol levels and thus may be easier to measure (Staufenbiel et al., 2013). Both cortisol and

DHEAS are HPA-axis hormones. DHEAS, produced in the adrenal cortex, typically increases when cortisol increases, and has a protects against long-term cortisol exposure as it turns the stress system back to homeostasis (van Dammen et al., 2020).

Trauma-exposed individuals have shown HCC *hypo*secretion, i.e., individuals exposed to traumatic experiences have lower levels of hair cortisol than non-trauma exposed individuals (Steudte et al., 2013). However, stress-exposed groups show increased HCC, whereas groups with anxiety disorders show lower HCC (Stalder et al., 2017). To our knowledge, HCC has not yet been studied in labour migrant populations, but it has been studied in various working populations. In studies with shift workers, results have been mixed; compared to day-workers, shift workers have been found to have lower mean HCC levels (Janssens et al., 2016) as well as higher mean HCC levels (Manenschijn et al., 2020). Furthermore, workers with symptoms of depression have been found to have higher mean HCC levels than workers without symptoms of depression (Janssens et al., 2016). Higher perceived job insecurity has also been correlated higher HCC (Herr et al., 2017). Among fishermen, HCC has been found to be higher and cortisone levels were lower before going on a fishing trip, compared to those returning from a fishing trip (Wu et al., 2021). Therefore, we expect that the DWM/PM+ intervention will lead to improved regulation of cortisol levels in labour migrants in the Netherlands.

In addition to cortisol, cortisone, and DHEAS, testosterone and progesterone levels can be detected in hair and they have also been linked to stress. Progesterone has been suggested to mediate glucocorticoid reactivity in PTSD (Inslicht et al., 2006). For testosterone, results are mixed. Some studies have found lower testosterone levels to be related to trauma exposure with no difference for individuals with PTSD symptoms (Reijnen, Geuze, & Vermetten, 2015; Feklicheva et al., 2022) while other studies have found higher testosterone levels for PTSD without comorbidity (Karlovic et al., 2012) or no difference (Spivak et al, 2003).

After written consent of the participant, we will collect ~100 strands of hair, as close as possible to the scalp. The samples will be analyzed at the laboratory of Kirschbaum in Dresden, Germany for HCC, cortisone, DHEA(S), progesterone, and testosterone levels. We will also ask questions related to the hair of participants (e.g. how often the hair is washed) as well as substance use (e.g. whether corticosteroids have been used in the past 6 months).

As participants and researchers don’t meet face-to-face, we will send participants that gave permission to give hair samples a toolkit with instructions on how to take the hair sample

(both an instruction letter with pictures as well as a link to a video in their own language with instructions how to take the hair sample), and everything they need in order to store and send the hair sample to us (e.g. cord to bind the hairs together, foil in which the hair can be stored, a return envelope).

Digital markers

Accurate measurement of cognitive functioning is essential to identify the best treatment option, track treatment response, as well as identify risk factors for the development of adverse effects. Traditional measurement techniques of cognitive functioning have multiple disadvantages: they are costly, burdensome, only infrequent assessment is allowed due to potential practice effects, and if computerizes assessment is not possible there is also the barrier of access to professionals who can administer these assessments. Thus, there is a need for non-invasive, low-burden, frequent methods to measure cognitive functioning (Schultebraucks et al., 2020).

The answer to this need may be found in the rapid rise and development of digital products and software. By matching traditional assessments with digital information, such as speech, voice, facial, and movement data, machine learning algorithms can be developed to identify digital phenotypes of cognitive functioning that can be passively measured. Such digital markers are sensitive biological outputs that depend on cognitive functioning (Cummins et al., 2015) and they have been linked to neuropsychiatric functioning (Schultebrauks et al., 2020). For instance, slowed and spaced out speech, reduced facial expressivity and movement, altered vocal characteristics have been found in suicidal patients and people suffering from major depressive disorder (Galatzer-Levy et al., 2020). In a study by Abbas et al. (2021), Participants suffering from major depressive disorder received a 4-week antidepressant treatment and demonstrated signitifant increases in head movement, amount of speech, and facial expessivity, and well as a decrease in anger and fear facial expressions.

In addition to the development of methodologies that use digital data sources to measure cognitive functioning, mental health care interventions are more often delivered remotely, a shift that has been accelerated due to the restrictions of the COVID-19 pandemic (Daigle & Rudnick, 2020). Not only does this give an opportunity to further develop the usage of digital markers, it also increases the possibility to apply it in clinical practice in the future. However, although these measurements are promising, these metrics need to be validated before they can be applied in clinical practice. To our knowledge, so far, digital markers have only been

explored among patients suffering from major depressive disorder and have not yet been explored amongst distressed populations like the one in RESPOND.

After written consent to video-record PM+ sessions for fidelity checks and further analysis of digital markers of facial activity (e.g. *happiness, sadness, fear, anger, surprise, disgust, and overall expressivity*), voice (e.g. *speech prevalence*), and movement (e.g. *head pitch and head yaw variability*) will be conducted (Galatzer-Levy et al., 2020).

Treatment fidelity

Process monitoring of the full stepped-care intervention includes review of helpers' records of DWM phone calls and PM+ sessions with clients; helpers' supervision records including intervention fidelity monitoring, and supervision of supervisors by intervention trainers. Tracked app usage information of the DWM app and audio records of the DWM phone calls and video records of the PM+ sessions will be analysed by VU Amsterdam research assistants and used for treatment fidelity analysis (see 5.1 Investigational product/treatment). The data will be collected throughout the intervention delivery (see Table 1) and reviewed as it is collected, leading to an iterative process of intervention monitoring informing intervention delivery.

> To monitor treatment fidelity of DWM, participants' usage of the DWM app will be tracked (see 6.3, study procedures - *Assessment of treatment fidelity*) and the helper phone calls will be audio-recorded. To monitor treatment fidelity of PM+, treatment sessions will be video-recorded. Giving consent to the audio or video recording is no requirement to receive the DWM/PM+ program. Audio and video records will be coded by research assistants from Vrije Universiteit Amsterdam and used for treatment fidelity analysis. In order to determine whether the intervention-as-implemented does not differ from the intervention-as-designed, fidelity checklists filled out by Vrije Universiteit Amsterdam research assistants are completed for a random sample, stratified on helpers, of sessions / participants. The data will be collected throughout the intervention delivery and reviewed as it is collected, leading to an iterative process of intervention monitoring informing intervention delivery. Treatment fidelity will be analyzed as manipulation check. If a participant give us permission to make audio or video records of the DWM/PM+ conversations, then they are stored until the end of the research and we have performed all fidelity checks. After this, audio records will be destroyed. No transcripts are made of these audio or video-files. If audio or video-files are transferred online, this will be done securely, e.g. by using SURFfilesender, and encrypting the file with a password.

Satisfaction and acceptability

Satisfaction and acceptability of the stepped-care DWM/PM+ intervention is measured through qualitative process evaluation (see Study Phase 3).

Implementation indicators

After the intervention has finished, various implementation indicators will be assessed, such as reach, dose, resource use, costs of recruiting, training and retaining staff delivering the stepped-care program, program costs, adaptation, the process and quality of the stepped-care DWM/PM+ intervention.

Additionally part of the cost-effectiveness analysis, we will estimate the incremental cost per change in the primary outcome, as well as quality of life. To do this, estimates of the resource use and costs of implementation are needed, making use of data from implementation indicators. This will involve analysis of records on resources and costs for initial training, as well as use of process and fidelity data on resources used for receipt of interventions, such as the number of PM+ sessions attended and input and support from supervisors.

Study phase 3

Study phase 3 consists of a qualitative study. The aim of this study is to explore the feasibility, i.e. identifying barriers and facilitators specific to the target population, of scaling-up the implementation on the stepped-care DWM/PM+ intervention within the Netherlands. This will be done by conducting in-depth semi-structured interviews and focus group discussions (FGDs) with key informants. In these interviews, participants' satisfaction and acceptability of the program will also be explored.

Key informants will include participants in the treatment group who completed the DWM intervention (n=4; improved) or the PM+ intervention (n=8; improved (n=4) and not improved (n=4)), who dropped-out during DWM (n=4) or during PM+ (n=4), and family members (or close persons) of participants in the treatment group who completed the DWM or PM+ intervention (n=6) or dropped out during DWM or PM+ (n=6). Participants and their family members will be asked questions concerning the satisfaction and acceptability of the intervention, barriers and facilitators to adherence, and to what extent they think that the stepped-care program has actually contributed to improving participants' functioning. Recruitment for participants of the treatment group and their family members will start at 3 months post-PM+.

Additionally, we will interview (a) mental health practitioners of the participating centres, (b) local stakeholders of the participating centres, e.g. mental health specialists and supervisors,

with a role in policy development or implementation, (c) clinical staff in primary (e.g. GPs, social workers) and secondary (e.g. psychologists) care, and (d) local and national policy makers with knowledge on mental health care (20-25 participants in total). Policy decision makers will be interviewed to obtain their perceptions of the benefits and challenges of integrating the stepped-care DWM/PM+ intervention into routine service provision. Health care professionals will be interviewed to explore their views on the potential for scaling-up the stepped-care DWM/PM+ intervention and integrating the program into the health system in the Netherlands. Furthermore, we will conduct focus group discussions (FGD) with facilitators (n=4-8) of the DWM/PM+ intervention. Facilitators will include both helpers and trainers/supervisors and we will balance for power of various stakeholders. Facilitators will be interviewed on their experience in providing the DWM/PM+ intervention and to obtain their ideas in implementing this intervention in the Netherlands. Interviews and FGDs will be conducted online or in person, depending on the preferences of the participant and will in accordance with COVID-19 regulations. The interviews with participants in the target group in phase 2 (both completers and drop-outs), their family members/close persons, and professionals will take approximately 1.5 hours. The FGDs with DWM/PM+ facilitators will take approximately 2 hours. Key informant interviews and FGDs will be audio-recorded and transcribed. The transcribed data will be coded and analysed using a qualitative data analysis software program such as Atlas.ti.

6.4 Withdrawal of individual subjects

Subjects can leave the study at any time for any reason if they wish to do so without any consequences. The investigator can decide to withdraw a subject from the study for urgent medical reasons.

6.4.1 Specific criteria for withdrawal (if applicable)

Not applicable for this study.

6.5 Replacement of individual subjects after withdrawal

No new subjects will be included for each withdrawn subject.

6.6 Follow-up of subjects withdrawn from treatment

If a subject decides to withdraw from the study, either in study phase 2 or 3, the investigator will ask for the reason. We will communicate to subjects that they are not obligated to answer this question. It will be enquired whether the subject wishes to withdraw from the study or from a specific time point only and so whether the

subject can be re-contacted at a later time. Withdrawal from the study will have no effect on the regular treatment. Subjects who leave the study for medical reasons will be followed until the interfering condition has resolved or reached a stable state.

6.7 Premature termination of the study

Not applicable.

7. SAFETY REPORTING

7.1 Temporary halt for reasons of subject safety

In accordance to section 10, subsection 4, of the WMO, the sponsor will suspend the study if there is sufficient ground that continuation of the study will jeopardise subject health or safety. The sponsor will notify the accredited METC without undue delay of a temporary halt including the reason for such an action. The study will be suspended pending a further positive decision by the accredited METC. The investigator will take care that all subjects are kept informed.

7.2 AEs, SAEs and SUSARs

7.2.1 Adverse events (AEs)

Adverse events are defined as any undesirable experience occurring to a subject during the study, whether or not considered related to the trial procedure or the stepped care DWM and PM+ intervention. All adverse events reported spontaneously by the subject or observed by the investigator or his staff will be recorded.

7.2.2 Serious adverse events (SAEs)

A serious adverse event is any untoward medical occurrence or effect that

- results in death;
- is life threatening (at the time of the event);
- requires hospitalisation or prolongation of existing inpatients' hospitalisation;
- results in persistent or significant disability or incapacity;
- is a congenital anomaly or birth defect; or
- any other important medical event that did not result in any of the outcomes listed above due to medical or surgical intervention but could have been based upon appropriate judgement by the investigator.

An elective hospital admission will not be considered as a serious adverse event.

The investigator will report all SAEs to the sponsor without undue delay after obtaining knowledge of the events, except for the following SAEs: Not applicable.

The sponsor will report the SAEs through the web portal *ToetsingOnline* to the accredited METC that approved the protocol, within seven days of first knowledge for SAEs that result in death or are life threatening followed by a period of maximum of eight days to complete the initial preliminary report. All other SAEs will be reported within a period of maximum 15 days after the sponsor has first knowledge of the serious adverse events.

7.3 Follow-up of adverse events

All AEs will be followed until they have abated, or until a stable situation has been reached. Depending on the event, follow up may require additional tests or medical procedures as indicated, and/or referral to the general physician or a medical specialist. SAEs need to be reported till end of study within the Netherlands, as defined in the protocol

7.4 Ethics and Data Advisory Board (EDAB)

The RESPOND' Ethics and Data Advisory Board (EDAB) will monitor and provide expert advice on data management and all ethical, legal and societal issues that arise within the project, promoting integrity and a better alignment of RESPOND with social needs and expectations that may arise within or as a result of RESPOND. This includes monitoring the safety, rights, and wellbeing of study participants, and providing input for ethics reports. In addition, the EDAB will provide advice on FAIR data management, including data privacy and adherence to the GDPR. The EDAB will ensure that the trial and data collection in RESPOND are conducted in accordance with the International Conference on Harmonisation (ICH), the WHO Good Clinical Practice standards (GCP), Declaration of Helsinki (64th WMA General Assembly, Fortaleza, Brazil, October 2013), and (inter)national laws (e.g., Medical Research Involving Human Subjects Act (WMO)). In addition, the ethical, safety and legal issues of the participants and research staff members will be reviewed such as incidental findings. Incidental findings within RESPOND refer to an extreme score on study instruments (questionnaires or interviews) that need additional follow-up. Other issues that will be considered include privacy and intellectual property rights. Relevant issues will be discussed in an annual meeting, but if issues arise between these meetings, the EDAB will be requested to plan an additional meeting. Additional meetings will be held before submission of ethics documents for formal approval as well as before submission of ethics reports. The EDAB compromises of independent members having no conflict of interest with the sponsor of the study, i.e. dr. Christopher Dowrick (emeritus professor of primary medical care including mental health), dr. Victor Perez (professor of psychiatry), Tom Paffen (senior legal counsel Privacy and Data Protection VU) and dr. Sonja Rutten (psychiatrist and member of the

Ethics Review Committee Board (VUmc). For RESPOND, principal investigator Prof. dr. Marit Sijbrandij will join the EDAB meetings together with assistant professor Dr. Anke Witteveen. . Prof. dr. Sijbrandij and dr. Witteveen only have an informative role in the EDAB; they are not involved in decision making or voting, nor in approving the meeting minutes.

The management team and EDAB will ensure that all necessary actions will be undertaken to minimize risks and suggest necessary measures to counter these risks. Through efficient communication between the EDAB, overall management (Work Package 1), and leader of individual Work Packages, the consortium will ensure that mitigation measures will be undertaken in a timely and effective manner.

The advice(s) of the EDAB will only be sent to the sponsor of the study. Should the sponsor decide not to fully implement the advice of the EDAB, the sponsor will send the advice to the reviewing METC, including a note to substantiate why (part of) the advice of the EDAB will not be followed. The EDAB should conclude each review with their recommendations to RESPOND as to whether the study should continue without change, be modified, or be terminated. Recommendations regarding modification of the design and conduct of the study could include: modifications of the study protocol based upon the review of the safety data; suspension or early termination of the study or of one or more study arms because of serious concerns about subjects' safety, inadequate performance, or rate of enrolment.

8. STATISTICAL ANALYSIS

The statistical analyses for the Randomized Controlled Trial are described under section 8.1-8.2 below.

8.1 Primary study parameter(s)

The statistical analysis of the RCT will estimate effectiveness of the stepped-care DWM/PM+ intervention with PFA and CAU compared to PFA and CAU alone, with PHQ-ADS score as the primary study parameter.

The primary outcome will be summarized using number of subjects (*n*), minimum and maximum; and means, standard deviations (*SD*) for normally distributed data, or medians and inter-quartile ranges for non-normally distributed data. To measure comparisons at baseline between the two treatment groups *t*-tests (continuous variables) or chi-squared test (categorical variables) will be conducted for normally distributed data; Mann-Whitney tests will be conducted for continuous non-normally distributed data.

Both intention-to-treat (ITT) analysis, including all randomized participants (*n*=212), and completers' (per protocol - PP) analysis will be carried out. The main conclusion of the trial will be based on the ITT analysis of the primary outcome. A secondary analysis of the primary outcome will also be presented using the PP population.

The statistical analysis will be masked, i.e. the trial statistician will be blinded to the treatment groups until the analysis has been completed. Moreover, the trial statistician will not be involved in determining participants' eligibility, in administering the intervention, in measuring the outcomes or in entering data.

To estimate the treatment effect, a linear mixed model will be employed for the primary endpoint analysis, which will have treatment as fixed effects, baseline measurement of primary endpoint as covariate, and subject as random effects. The mean difference between two treatment arms at each visit/time together with its 95% confidence interval will be derived from the mixed model. Covariate-adjusted mixed model of primary endpoint will also be performed by adding pre-specified covariates at baseline (gender, age, education, adverse (traumatic) events, COVID-19 related events, and severity of symptoms) into the above model.

Missing data

Missing data will be treated as missing at random (MAR). No imputations of missing values will be made, as multilevel models can deal with missing data (Singer, Willett & Willett, 2003).

8.2 Secondary study parameter(s)

Economic outcomes

Health economic analysis will be conducted to determine the difference in costs and outcomes in the intervention arm as compared to the care as usual group. Primary analysis will be the total costs over the 2-month follow-up treatment period. Between-group comparison of mean costs will be completed using standard *t*-test with ordinary least squares regression used for adjusted analysis, with the validity of results confirmed using bootstrapping. Pseudonimised data will be sent to the London School of Economics and Political Science, partner in RESPOND under Work Package 3, for the health economics analysis of the CSRI.

Analysis of secondary outcomes with repeated measurements

Additionally, a linear mixed model as mentioned for the primary outcome analysis (PHQ-ADS) will be carried out for analysing the following clinical outcomes measured at baseline, at week 6 (about 1 weeks after DWM), at week 12 and at week 20 (about 1 week and 2 months after finishing PM+): posttraumatic stress reactions (PCL-5), depressive symptoms (PHQ-9), generalized anxiety (GAD-7), resilience (MIMIS) and quality of life (EQ-5D-5L).

Analysis of other secondary outcomes

Changes in caseness of the composite measure anxiety and depression will be calculated for the PP sample using the recommended cut-off of >20 for moderate severity on the PHQ-ADS questionnaire (Kroenke et al., 2016; Kroenke et al., 2019) and will be analysed using a hierarchical logistic model with the same fixed and random effects as the hierarchical linear models above, from which odds ratio of having a depression together with 95% CI at each time point will be derived.

Corrections for multiple testing

Models will be tested on $\alpha = .05$; we will not apply a post-hoc correction to deal with problems associated with multiple testing, but instead report the number of tests that are carried out.

8.3 Other study parameter(s)

Phase 3 of this RCT will consist of qualitative interviews and/or focus group discussions among participants and key stakeholders to evaluate possible barriers and facilitators to treatment engagement and adherence to the PM+/DWMS program. The outcomes of these assessments will be used to make informed-decisions for potential mediators or moderators of PM+/DWMS treatment effectiveness.

Hair biomarkers

In all hair samples, HCC, cortisone, DHEAS, progesterone and testosterone levels will be analyzed through liquid chromatography-tandem mass spectrometry (LC-MS/MS). In addition, HCC/cortison and cortisol/DHEAS ratios will be calculated. Hormone levels at baseline (T1) of participants that were included (K10>15.9) and excluded (K10<15.9) will be compared and linked to answers given to T1 assessment. Of participants that were included, Differences between baseline (T1) and 20-week follow-up (T4) levels between the intervention and control group will be compared as well as linked to answers given at assessments.

Digital markers

Digital markers will be assessed using open source tools in a Python environment, which is available on Github (Abbas, 2020), similar to the analysis in Galatzer-Levy et al. (2020). Results will be linked with responses to primary and secondary outcomes.

Treatment fidelity:

In order to determine whether the intervention-as-implemented does not differ from the intervention-as-designed, fidelity checklists filled out by VU Amsterdam research assistants are completed for a random sample, stratified on peer-migrant PM+ providers of sessions/participants. Treatment fidelity will be analysed as manipulation check.

8.4 Interim analysis (if applicable)

Not applicable.

9. ETHICAL CONSIDERATIONS

9.1 Regulation statement

This study will fully comply with relevant European and national regulations concerning data protection, privacy regulations, and the procedures for obtaining informed consent. All these procedures will be conducted according to the Declaration of Helsinki (64th WMA General Assembly, Fortaleza, Brazil, October 2013), in accordance with, the EU Good Clinical Practice Directive (2001/20/EC), the International Conference on Harmonization (ICH), the WHO Good Clinical Practice Standards (GCP), and the Medical Research Involving Human Subjects Act (WMO).

9.2 Recruitment and consent

Recruitment

Study Phase 1

Study phase 1 is described in a separate protocol for which ethical approval has been granted by the VCWE.

Study Phase 2

Eligible trial participants will be adult (18 years or above) labour migrants without acute medical conditions (see inclusion and exclusion criteria in paragraph 4.2 and 4.3). The recruitment strategy for the RCT will be pragmatic and will involve relationships with community organizations and local institutions that implement services/projects for labour workers in the Netherlands (e.g. municipalities, NGOs, health care providers, and/or language courses). Participants will also be recruited through targeted (social) media strategies and snowballing.

We will inform these organizations about our trial, distribute flyers/posters, and post (the text of) these flyers/posters also on (social) media. Whenever someone is interested in participating in this trial, the potential participant will have to reach out to us themselves (e.g. through e-mail), or stakeholders can inform the research team that a potential participant is interested in this study (but only with permission of the potential participant which we will check).

Study Phase 3

Participants: If participants in study phase 2 indicate in the consent form that they give permission to be approached for follow-up research, we may ask the participant to participate in an interview.

Family members/close friends of participants: Recruitment of family members/close friends of participants will happen via participants in the RCT who gave permission in the consent form to be approached for follow-up research; we may also ask them to ask their family members/close friends, on our behalf, if they would like to participate in an interview. If they want to participate, these family members/close friends will either need to contact us or we approach them by e-mail/telephone after they have given consent to the participant and this participant has provided us with their e-mail/telephone number.
Professionals: In study phase 1, we interviewed professionals, such as (mental) health practitioners and policy makers. Professionals who stated in the informed consent form of study phase 1 that they can be approached for further research, may be contacted for study phase 3 by e-mail, as we saved their contact information. These professionals may also provide us with additional contacts from their network. We will ask these professionals if they can ask on our behalf if their additional contacts can reach out to us via e-mail or phone if they are interested in participating.
Facilitators: supervisors/helpers of the DWM/PM+ interventions will be approached through e-mail for participation in a FGD. We have their e-mail as we work together throughout study phase 2. During the recruitment of the facilitators (supervisors/helpers of phase 2) they are informed that they will be asked for participation in a FGD after completion of the RCT.

For study phase 3, a purposive sampling method called, maximum variation sampling will be used. In this sampling method, the sample is selected based on variations of some key characteristics (Suri, 2011). For the treatment group participants relevant variables will include gender, age, country of origin, and status of completing the program (drop out or completed). These participants will be asked for consent to reach out to one of their family members or a close person as well. Selection of DWM and PM+ helpers will also be based on maximum variation and the relevant variables will include gender, age, country of origin, and the type of intervention (DWM and/or PM+) provided. Mental health specialists/supervisors involved in the trial will be directly approached if they want to participate to the interviews. Decision makers in the key stakeholders will be approached through available professional contacts.

Informed consent procedure

Study Phase 2

Before being enrolled in the study, participants will be informed about the nature and scope of the study in a form understandable to them. Participation will be completely voluntary to prevent biased responding – either exaggerating or minimizing problems in the belief that this may help participants obtaining secondary advantages.

Once a potential participant has reached out to us and expressed interest in participating in this trial, a research assistant will meet with the potential participant at a later time (in person or, due to COVID-19 regulations, through telephone or teleconferencing). The research assistant will explain the research to the potential study participant. If a potential participant is still interested in participating in this trial after receiving verbal information, we provide the participant with one written information letter and one informed consent form. This will be in person by the research assistant or digitally through e-mail as a .pdf document (see Information letter participants, Phase 2 - RCT). When requested by the participant, we will also send a printed version of the information letter and consent form to the participant.

The research assistant will have a cultural background similar to the participant, because most labour migrants are not familiar with the local language. These research assistants will be briefed on the aims of the study, study procedures, and ethical obligations (e.g. confidentiality, right to refuse to answer questions) and will be required to formally declare that they have truthfully and faithfully translated the explanation of the research project to the best of their skills and ability.

After a minimum consideration time of one week, the research assistant will reach out to the potential participant and ask what they have decided regarding their participation. If a potential participant has additional questions, (s)he can ask them immediately or the main investigator. Of course, the potential participant can always also reach out to the independent expert whose contact details are stated in the information letter (appendix A).Persons who decide to participate in the trial will be asked to complete a digital consent form through the VU instance (licence) of Qualtrics. This informed consent form is for participation in the full study, i.e. both the screening and the study.

After receiving the digital signed informed consent form, it will be signed by the research team and e-mailed to the participant, so that they also have a digital version of the signed consent form. Additionally, a research assistant will reach out to the participant to set up a teleconferencing meeting with the participant for screening (T0). If participants meet the eligibility criteria (K10 >15.9), they can participate in the trials. If a participant does not meet the eligibility criteria (K10 ≤ 15.9 or having a severe cognitive impairment/mental disorder/imminent suicide risk), participation will end after the screening.

Participants are allowed to withdraw from the study at any time after they have given their written consent. Participants can report this in various way, e.g. digitally by e-mail, or over the telephone. Participants who are enrolled in the stepped-care DWM/PM+ treatment group and who are still eligible for PM+ after they participated in DWM, their PM+ sessions will be video recorded if they gave permission for this in the informed consent form they signed prior

to the start of the study (see Information letter and informed consent form). Giving consent for the audio or video recordings is not a condition for participating in the study. DWM audio recordings will only be used for fidelity assessments and supervisions. PM+ video recordings will be used for both fidelity assessments and to explore digital markers.
This consent procedure is in line with the handbook on grantic electronic consent by the CCMO/NVMETC, dd. 22-08-2022 (see: https://www.ccmo.nl/metcs/publicaties/publicaties/2022/08/31/handreiking-elektronische-toestemmingsverlening).

Study Phase 3

If a trial participant (study phase 2), a family member/close friend, a professional, or a facilitator contacts us to express their interest in participating in an interview/FGD, someone from the research team will reach out to the potential participant and explain the interview to him/her by telephone or through teleconferencing. If a potential participant is still interested in participating in this trial after receiving verbal information, we provide the participant with one written information letter and one informed consent form through e-mail (as .pdf) (see Information letters, Phase 3). When requested, we will also send a printed version of the information letter and consent form to the participant.
After a minimum consideration time of one week, someone from the research team will reach out to the potential participant to ask what they have decided regarding their participation. If a potential participant has additional questions, (s)he can ask them immediately or reach out to the main investigator or independent expert whose contact details are stated in the information letter (appendix A).

If persons want to participate and can meet in person for the interview, a moment and location, e.g. Vrije Universiteit Amsterdam, for an interview will be set-up. In that case, the informed consent form will be given to the participant by the interviewer (someone from the research team) and signed at the beginning of the interview. In case it is not possible to meet in person, e.g. due to COVID-19 regulations, participants will be asked to complete a digital consent form through the VU instance (licence) of Qualtrics. After the participant has signed the digital consent form, it will be signed by the research team and e-mailed to the participant, so that they also have a digital version of the signed consent form.
After filling in the consent form, someone from the research team will reach out to the participant to set up a teleconferencing meeting to conduct the interview. Participants are allowed to withdraw from the study at any time after they have given their written consent. They can do so in various way, e.g. digitally by e-mail, or over the telephone.

This consent procedure is in line with the handbook on grantic electronic consent by the CCMO/NVMETC, dd. 22-08-2022 (see: https://www.ccmo.nl/metcs/publicaties/publicaties/2022/08/31/handreiking-elektronische-toestemmingsverlening).

9.3 Objection by minors or incapacitated subjects (if applicable)

Not applicable.

9.4 Benefits and risks assessment, group relatedness

Study phase 2:

Participants randomized into the DWM/PM+ treatment group may benefit from their participation in terms of expected reductions in psychological distress. The risks associated with participation are estimated to be minimal, since DWM and PM+ reduced psychological distress in previous studies (Purgato et al., 2019; Tol et al., 2020; Bryant et al., 2017; Rahman et al., 2016b). Participants in both the treatment and comparison group will not be withheld care as usual (note: the treatment group receives CAU along with stepped-care DWM/PM+ and psychological first aid (PFA), the comparison group will receive CAU and PFA only).

It is possible that participants experience (increased) stress during the PM+ sessions. The intervention will be supervised and strictly monitored by experienced psychologists. If a participant deteriorates during the intervention period, (s)he can be referred to an external specialist (licensed psychologist or psychiatrist) through contact with the general practitioner. In case a participant does not have a general practitioner, the research team will contact a general practitioner nearby the participants' home and make sure that (s)he will be registered and/or can get an appointment with a general practitioner. Whether referral has taken place, will be actively followed up by the researchers. Participants may also experience distress during the assessment interviews. In case a participant experiences passive or active suicidal ideation, measurement instruments in the online questionnaire will identify this and an automated message will be sent to the researchers, who will then take action (see 6.3 Study procedures > Screening instruments for exclusion criteria > suicidal ideation). When a participant has elevated symptoms at follow-up assessments, an automated message will be sent to the researchers and (s)he will be advised to contact his/her general practitioner (part of the CAU), who may refer the participant for continued or high-intensity treatment (i.e., stepped-care process leading to more specialized care high-intensity treatment).

Study phase 3:

The benefit of participating in this study is that the input of the participants may contribute to improved mental healthcare for the target population in the Netherlands. At the end of the study, after data has been analyzed, results will be shared with participants if they have stated in the informed consent form that they want this.
No harm is expected, since participants will either be healthy volunteers (family members/professionals) or participants from the treatment group in study phase 2 (who are potentially recovered from their symptoms of distress), and the study is not focused on personal problems or distress of the people who are interviewed. Instead, participants are asked to provide information about their experiences with the intervention or their view on the acceptability and implementation of the DWM/PM+ intervention. Participants will not be asked about traumatic events.
Loss of privacy is a potential risk in the FGDs as they are in a group format. However, the FGD will only be conducted with facilitators of the intervention (i.e. supervisors and helpers) who know each other as they have weekly sessions throughout study phase 2 and thus will be familiar with all of their experiences with delivering the intervention. Nevertheless, to avoid any kind of privacy issues, the interviewer will always begin the FGD by giving information about the privacy of the discussed issues and then will ask respondents to keep everything discussed in the FGDs confidential. At the end of the FGDs, the interviewer will remind participants of their commitment to confidentiality.

9.5 Compensation for injury

The Vrije Universiteit has a liability insurance which is in accordance with article 7 of the WMO. Participation in the study only carries negligible risks for the research subjects; therefore, the present study is relieved from an insurance that provides cover for damage to research subjects.

9.6 Incentives (if applicable)

<u>Study phase 2:</u>
If applicable, study participants will receive travel reimbursements during the study. Participants will also receive 10 euros (monetary or in vouchers) per assessment for the first (baseline – week 0), second (1 week after the end of the DWM intervention – week 6), third (1-week after the end of the PM+ intervention – week 12), and fourth assessments (2-months after the end of the PM+ intervention – week 20). Participants who are excluded because they score too low on the screener K10 (<15.9) but who are still willing to give a hair sample and fill in T1 also receive 10 euros.

Study phase 3:

If applicable, all study participants in study phase 3 will receive travel reimbursements if they have to travel for a face-to-face interview. Additionally, participants from study phase 2 and their family members/close friends receive 20 euros (monetary or in vouchers) per interview.

ADMINISTRATIVE ASPECTS, MONITORING AND PUBLICATION

9.7 Handling and storage of data and documents

All data will be handled confidentially and will be coded by a code known only by the research team. Processing of personal data will comply to the General Data Regulation ("GDPR") (in Dutch: Algemene verordening gegevensbescherming, AVG) on the protection of individuals regarding the processing of personal data and the free movement of such data. Additionally, this study adheres to the Research Data Management Policy of Vrije Universiteit Amsterdam (see https://libguides.vu.nl/ld.php?content_id=32045526), as well as the Research Data Management Policy of Faculty of Behaviour and Movement Sciences (see https://libguides.vu.nl/ld.php?content_id=32204773).

Data including personal information will be stored in a locked record in Vrije Universiteit to ensure the confidentiality of the study participants. Only authorized research personnel would have access to this data. According to the data management rules of RESPOND, all partners acknowledge and agree that no personal data, as defined in Regulation (EU) 2016/679 of the European Parliament and of the Council of 27 April 2016 on the protection of natural persons with regard to the processing of personal data and on the free movement of such data, and repealing Directive 95/46/EC (General Data Protection Regulation) ("GDPR"), will be exchanged between the Parties. Moreover, all partners in RESPOND acknowledge and agree that each partner is considered independent controller, as defined in GDPR, for its processing of personal data and will act in accordance with applicable data protection laws (including but not limited to GDPR).

Qualitative data collection

Qualitative data is collected both in phase 2 and phase 3. During phase 2, audio files (from the DWM support calls) and video files (from the PM+ sessions) are all created with MS Teams. During phase 3, qualitative interviews will be recorded on a professional audio recording device.

Before commencing a qualitative interview, the date of the interview/date of the PM+ session and the participant number will be recorded.

The audio and video files for study phase 2 will not be transcribed, as they are only used for the purpose of fidelity checks (audio and video files) and the analysis of digital markers (video files). No identifying information will be collected during the qualitative interviews of study phase 3 (interviews), with all data de-identified. The audio files for study phase 3 will be transcribed by the research team in the Microsoft word program (secured with a password known to the research team only).

All audio and video files (mp3 and mp4) of both study phase 2 and 3 will be encrypted and stored in a folder on Surfdrive (a secure cloud service), that can only be accessed by the members of Vrije Universiteit Amsterdam research team. If audio or video-files are transferred online, this will be done securely, e.g. by using SURFfilesender, and encrypting the file with a password. The audio and video files will be saved separately from their identifiers. The identifying key (a list connecting names to numbers) will be kept in a separate, secure locked location in the coordinating researcher's office (prof. dr. Marit Sijbrandij). After transcription of the audio files of study phase 2, the mp3 files will be destroyed. After analysis of the data, transcripts of study phase 2, and audio and video files of study phase 3 will be archived in DarkStor.

The qualitative data of Study Phase 1 will be available for 5 years after the completion of the study. Both qualitative and quantitative data of Phase 2 and Phase 3 will be available for 15 years after the completion of the study, which is in line with the Research Data Management Policy of Vrije Universiteit Amsterdam (see: https://libguides.vu.nl/ld and Movement Sciences (see: https://libguides.vu.nl/ld.php?content_id=32204773).

Quantitative data collection

Quantitative data will be coded and the identifying key (a list connecting names to numbers) will be kept in a separate, secure locked location in the coordinating researcher's office (prof. dr. Marit Sijbrandij). A web-based electronic tool Castor EDC (https://www.castoredc.com/) will be used to collect, automatically validate, and safely store data. For this programme there is a processor agreement with the IT department of the VU ICT. The subjects have access to the questionnaires via an automated link via the research website/e-mail. At any time during the study and afterwards, data will be exportable in electronic formats (in particular MS Excel), being broadly compatible with all relevant statistical software. The data will be entered into a data-analytic computer program (e.g., SPSS), without the identifying key. All data will be stored pseudonymous, and they will be available only to the Vrije Universiteit Amsterdam research team members. All pseudonymous data to be stored at Vrije Universiteit Amsterdam will be stored in a data repository named DataverseNL. DataverseNL is an online platform that can be used to share and publish research data in a (semi-)open environment. It is possible to link to a DataverseNL dataset in publications. The Vrije Universiteit Amsterdam research group will analyze the data, and both positive and negative trial results will be disclosed. No attributable data will be used in publications. Results will be submitted for publication to peer-reviewed scientific journals. After the completion of RESPOND, the list of participants will be stored in DarkStor, an offline research data

archive for sensitive data. Data in DarkStor are only accessible by authorized persons (principal investigator prof. dr. Marit Sijbrandij).

Hair samples

Similar to quantitative data, hair samples will be coded and the identifying key (a list connecting names to numbers) will be kept in a separate, secure locked location in the coordinating researcher's office (prof. dr. Marit Sijbrandij). Hair samples will be stored in a locked cupboard in the office of another researcher (dr. Anke Witteveen).
Hair samples are stored until the collection of hair samples is finished. Once data collection of the study is finished, all collected hair samples will be sent to the laboratory. This will be when we received the second hair sample (T4) of the last participant that gave permission to give hair samples. After analysis, hair samples are destroyed.

The METc will be informed about the effective start date of the RCT (Study Phase 2).

9.8 Monitoring and Quality Assurance

Process monitoring is a part of Study Phase 2 and more detailed explained under 'Study Procedures'. Monitoring includes review of Helpers' records of PM+, supervision records including intervention fidelity monitoring and supervision of supervisors by the master trainers. The supervision of helpers will be scheduled weekly. The supervision will be given by expert psychologists. The supervising psychologists will also receive supervision from the master trainers and these supervisions will be scheduled monthly. This is similar to the procedure used by Rahman and colleagues (2016) for the PM+ trial in Pakistan. One of the outcome measures of this study is the treatment fidelity. Audio records will be coded by Vrije Universiteit Amsterdam research assistants. The data will be collected throughout the intervention delivery (see Table 1). Monitoring of the assessments will be the responsibility of the main investigator (Rinske Roos). In case of any concerns about the capacity of the assessors to carry out their roles, psychologists will conduct full assessments to ensure quality. This oversight will help ensure that any potential concerns about the capacity of assessors to carry out their roles is picked up and responded to.

9.9 Amendments

Amendments are changes made to the research after a favorable opinion by the accredited METc has been given. All amendments will be notified to the METc that gave a favorable opinion.

9.10 Annual progress report

Vrije Universiteit Amsterdam will submit a summary of the progress of the trial to the accredited METC once a year. Information will be provided on the date of inclusion of the first subject, numbers of subjects included and numbers of subjects that have completed the trial, serious adverse events, other problems, and amendments.

9.11 Temporary halt and (prematurely) end of study report

The investigator/sponsor (Vrije Universiteit Amsterdam) will notify the accredited METc of the end of the study within a period of 8 weeks. The end of the study is defined as the last patient's last visit. The sponsor will notify the METC immediately of a temporary halt of the study, including the reason of such an action. In case the study is ended prematurely, the sponsor will notify the accredited METc within 15 days, including the reasons for the premature termination. Within one year after the end of the study, the investigator/sponsor will submit a final study report with the results of the study, including any publications/abstracts of the study, to the accredited METc.

9.12 Public disclosure and publication policy

The trial will be registered in a public trial registry (the Netherlands Trial Register; see http://www.trialregister.nl/trialreg/index.asp) before the first patient is recruited. The results of the study will be submitted for publication in international, peer-reviewed journals. Moreover, findings may will be presented in a scientific conferences and be disseminated to stakeholders working in the field. In addition to all, the results of the study will be disseminated through the WHO website and other dissemination channels of WHO. A preliminary version of the RESPOND Communication and Dissemination Plan has been delivered to the EU in February 2021.

REFERENCES

Aanjaagteam Bescherming Arbeidsmigranten (2020). Geen tweederangsburgers; Aanbevelingen om misstanden bij arbeidsmigranten in Nederland tegen te gaan.

Abbas A. anzarabbas/ms_dbms_mdd_suicidality [Internet]. 2020 [cited 2020 Oct 21]. Available from: https://github.com/anzarabbas/ms_dbms_mdd_suicidality

Abbas, A., Sauder, C., Yadav, V., Koesmahargyo, V., Aghjayan, A., Marecki, S., ... & Galatzer-Levy, I. R. (2021). Remote Digital Measurement of Facial and Vocal Markers of Major Depressive Disorder Severity and Treatment Response: A Pilot Study. *Frontiers in Digital Health*, *3*, 28.

Ashbaugh, A. R., Houle-Johnson, S., Herbert, C., El-Hage, W., & Brunet, A. (2016). Psychometric Validation of the English and French Versions of the Posttraumatic Stress Disorder Checklist or DSM-5 (PCL-5). PloS one, 11(10), e0161645. https://doi.org/10.1371/journal.pone.0161645

Attkisson, C. C., & Zwick, R. (1982). The Client Satisfaction Questionnaire: Psychometric properties and correlations with service utilization and psychotherapy outcome. Evaluation and program planning, 5(3), 233-237.

Baggaley, R. F., Ganaba, R., Filippi, V., Kere, M., Marshall, T., Sombie, I., ... & Patel, V. (2007). Detecting depression after pregnancy: the validity of the K10 and K6 in Burkina Faso. *Tropical Medicine & International Health*, *12*(10), 1225-1229.

Baillie, A. J. (2005). Predictive gender and education bias in Kessler's psychological distress scale (K10). *Social Psychiatry and Psychiatric Epidemiology*, *40*(9), 743-748.)

Banbury, A., Nancarrow, S., Dart, J., Gray, L., & Parkinson, L. (2018). Telehealth interventions delivering home-based support group videoconferencing: systematic review. *Journal of medical Internet research*, *20*(2), e25.

Blevins, C. A., Weathers, F. W., Davis, M. T., Witte, T. K., & Domino, J. L. (2015). The posttraumatic stress disorder checklist for DSM-5 (PCL-5): Development and initial psychometric evaluation. *Journal of traumatic stress*, *28*(6), 489-498.

Boeschoten, M.A., Bakker, A., Jongedijk, R.A. & Olff, M. (2014). PTSD Checklist for DSM-5– Nederlandstalige versie. Uitgave: Stichting Centrum ’45, Arq Psychotrauma Expert Groep, Diemen.

Boyd, L., Baker, E., & Reilly, J. (2019). Impact of a progressive stepped care approach in an improving access to psychological therapies service: An observational study. *Plos one*, *14*(4), e0214715.

Bryant, R. A., Schafer, A., Dawson, K. S., Anjuri, D., Mulili, C., Ndogoni, L., ... & Van Ommeren, M. (2017). Effectiveness of a brief behavioural intervention on psychological distress among women with a history of gender-based violence in urban Kenya: A randomised clinical trial. *PloS medicine*, *14*(8), e1002371.

Buchmüller, T., Lembcke, H., Busch, J., Kumsta, R., Wolf, O. T., & Leyendecker, B. (2020). Exploring hair steroid concentrations in asylum seekers, internally displaced refugees, and immigrants. Stress, 23(5), 538-545.

CBS (2020). Werknemers geboren in buitenland; wel/niet ingezeten, persoonskenmerken, https://opendata.cbs.nl/statline/#/CBS/nl/dataset/84750NED/table?ts=1610572083494

Chisholm, D., Knapp, M., Knudsen, H., Amaddeo, F., Gaite, L., & van Wijngaarden, B. (2000). Client Socio-Demographic and Service Receipt Inventory-European Version: development of an instrument for international research. EPSILON Study 5. European Psychiatric Services: Inputs Linked to Outcome Domains and Needs. British Journal of Psychiatry, 39, s28-33.

Chmitorz, A., Kurth, K., Mey, L. K., Wenzel, M., Lieb, K., Tüscher, O., ... & Kalisch, R. (2020). Assessment of Microstressors in Adults: Questionnaire Development and Ecological Validation of the Mainz Inventory of Microstressors. *JMIR mental health*, *7*(2), e14566.

Cuijpers, P., Muñoz, R. F., Clarke, G. N., & Lewinsohn, P. M. (2009). Psychoeducational treatment and prevention of depression: the “Coping with Depression” course thirty years later. *Clinical psychology review*, *29*(5), 449-458.

Conway, L.G., Woodward, S.R., & Zubrod, A. (2020). Social Psychological Measurements of COVID-19: Coronavirus Perceived Threat, Government Response, Impacts, and Experiences Questionnaires. PsyArXiv Preprints.

Daigle, P., & Rudnick, A. (2020). Shifting to remotely delivered mental health care: Quality improvement in the COVID-19 pandemic. *Psychiatry International*, *1*(1), 31-35.

Dawson, K. S., Bryant, R. A., Harper, M., Tay, A. K., Rahman, A., Schafer, A., & Van Ommeren, M. (2015). Problem Management Plus (PM+): a WHO transdiagnostic psychological intervention for common mental health problems. World Psychiatry, 14(3), 354.

Dawson, K. S., Schafer, A., Anjuri, D., Ndogoni, L., Musyoki, C., Sijbrandij, M., ... & Bryant, R. A. (2016). Feasibility trial of a scalable psychological intervention for women affected by urban adversity and gender-based violence in Nairobi. *BMC psychiatry*, *16*(1), 1-9.

de Graaff, A. M., Cuijpers, P., Acarturk, C., Bryant, R., Burchert, S., Fuhr, D. C., . . . Sijbrandij, M. (2020). Effectiveness of a peer-refugee delivered psychological intervention to reduce psychological distress among adult Syrian refugees in the Netherlands: study protocol. *European journal of psychotraumatology, 11*(1), 1694347. doi:10.1080/20008198.2019.1694347

Den Boer, P. C. A. M., Wiersma, D., & van den Bosch, R. J. (2004). Why is self-help neglected in the treatment of emotional disorders. *Psychol Med*, *34*(6), 959-971.

Donker, T., Comijs, H., Cuijpers, P., Terluin, B., Nolen, W., Zitman, F., & Penninx, B. (2010). The validity of the Dutch K10 and extended K10 screening scales for depressive and anxiety disorders. *Psychiatry research*, *176*(1), 45-50.

Donker, T., van Straten, A., Marks, I., & Cuijpers, P. (2011). Quick and easy self-rating of Generalized Anxiety Disorder: validity of the Dutch web-based GAD-7, GAD-2 and GAD-SI. *Psychiatry research*, *188*(1), 58-64.

Dragan, M., Grajewski, P., & Shevlin, M. (2021). Adjustment disorder, traumatic stress, depression and anxiety in Poland during an early phase of the COVID-19 pandemic. *European Journal of Psychotraumatology*, *12*(1), 1860356.

Dua, T., Barbui, C., Clark, N., Fleischmann, A., Poznyak, V., van Ommeren, M., ... & Saxena, S. (2011). Evidence-based guidelines for mental, neurological, and substance use disorders in low-and middle-income countries: summary of WHO recommendations. *PLoS Med*, *8*(11), e1001122.

Epping-Jordan, J. E., Harris, R., Brown, F. L., Carswell, K., Foley, C., García-Moreno, C., ... & van Ommeren, M. (2016). Self-Help Plus (SH+): a new WHO stress management package. *World Psychiatry*, *15*(3), 295.

EuroHealthNet (2020), Public Sector Responses to Addressing Mental Health Needs of the Covid-19 Crisis. Webinar #2.

EuroQol (2020). EQ-5D-5L, Self-complete version on paper. https://euroqol.org/eq-5d-instruments/eq-5d-5l-available-modes-of-administration/self-complete-on-paper/

Feklicheva, I., Boks, M. P., de Kloet, E. R., Chipeeva, N., Maslennikova, E., Pashkov, A., ... & Tseilikman, V. (2022). Biomarkers in PTSD-susceptible and resistant veterans with war experience of more than ten years ago: FOCUS ON cortisol, thyroid hormones, testosterone and GABA. Journal of Psychiatric Research, 148, 258-263.

Fledderus, M., Bohlmeijer, E. T., Pieterse, M. E., & Schreurs, K. M. G. (2012). Acceptance and commitment therapy as guided self-help for psychological distress and positive mental health: a randomized controlled trial. *Psychological medicine*, *42*(3), 485.

Furukawa, T. A., Kessler, R. C., Slade, T., & Andrews, G. (2003). The performance of the K6 and K10 screening scales for psychological distress in the Australian National Survey of Mental Health and Well-Being. *Psychological medicine*, *33*(2), 357.

Galatzer-Levy, I., Abbas, A., Yadav, V., Koesmahargyo, V., Aghjayan, A., Marecki, S., ... & Sauder, C. (2020). Remote digital measurement of visual and auditory markers of Major Depressive Disorder severity and treatment response. medRxiv.

Galatzer-Levy, I., Abbas, A., Ries, A., Homan, S., Sels, L., Koesmahargyo, V., ... & Kleim, B. (2020). Validation of Visual and Auditory Digital Markers of Suicidality in Acutely Suicidal Psychiatric Inpatients: Proof-of-Concept Study. *Journal of Medical Internet Research*, *23*(6), e25199.

Galenkamp, H., Stronks, K., Snijder, M. B., & Derks, E. M. (2017). Measurement invariance testing of the PHQ-9 in a multi-ethnic population in Europe: the HELIUS study. *BMC psychiatry*, *17*(1), 1-14.

Gambin, M., Sękowski, M., Woźniak-Prus, M., Wnuk, A., Oleksy, T., Cudo, A., ... & Maison, D. (2021). Generalized anxiety and depressive symptoms in various age groups during the COVID-19 lockdown in Poland. Specific predictors and differences in symptoms severity. *Comprehensive Psychiatry*, *105*, 152222.

Garcy, A. M., & Vågerö, D. (2013). Unemployment and suicide during and after a deep recession: a longitudinal study of 3.4 million Swedish men and women. *American journal of public health*, *103*(6), 1031-1038.

Hayes, S. C., Levin, M. E., Plumb-Vilardaga, J., Villatte, J. L., & Pistorello, J. (2013). Acceptance and commitment therapy and contextual behavioral science: Examining the

progress of a distinctive model of behavioral and cognitive therapy. *Behavior therapy*, *44*(2), 180-198.

Herdman, M., Gudex, C., Lloyd, A., Janssen, M. F., Kind, P., Parkin, D., ... & Badia, X. (2011). Development and preliminary testing of the new five-level version of EQ-5D (EQ-5D-5L). *Quality of life research*, *20*(10), 1727-1736.

Herr, R. M., Barrech, A., Gündel, H., Lang, J., Quinete, N. S., Angerer, P., & Li, J. (2017). Effects of psychosocial work characteristics on hair cortisol–findings from a post-trial study. *Stress*, *20*(4), 363-370.

Hinz, A., Klein, A. M., Brähler, E., Glaesmer, H., Luck, T., Riedel-Heller, S. G., ... & Hilbert, A. (2017). Psychometric evaluation of the Generalized Anxiety Disorder Screener GAD-7, based on a large German general population sample. *Journal of affective disorders*, *210*, 338-344.

Ho, F. Y. Y., Yeung, W. F., Ng, T. H. Y., & Chan, C. S. (2016). The efficacy and cost-effectiveness of stepped care prevention and treatment for depressive and/or anxiety disorders: a systematic review and meta-analysis. *Scientific reports*, *6*(1), 1-10.
John Hopkins University and Medicine (2020), "Coronavirus Resource Center," https://coronavirus.jhu.edu

Hobfoll, S.E.; Watson, P.; Bell, C.C.; Bryant, R.A.; Brymer, M.J.; Friedman, M.J.; Friedman, M.; Gersons, B.P.; de Jong, J.T.; Layne, C.M.; et al. Five essential elements of immediate and mid-term mass trauma intervention: Empirical evidence. Psychiatry 2007, 70, 283–315.

Inslicht, S. S., Marmar, C. R., Neylan, T. C., Metzler, T. J., Hart, S. L., Otte, C., . . . Baum, A. (2006). Increased cortisol in women with intimate partner violence related posttraumatic stress disorder. Annals of the New York Academy of Sciences, 1071, 428– 429. doi:10.1196/annals.1364.035

Karlović, D., Serretti, A., Marčinko, D., Martinac, M., Silić, A., & Katinić, K. (2012). Serum testosterone concentration in combat-related chronic posttraumatic stress disorder. Neuropsychobiology, 65(2), 90-95.

Kertz, S., Bigda-Peyton, J., & Bjorgvinsson, T. (2013). Validity of the Generalized Anxiety Disorder-7 Scale in an acute psychiatric sample. *Clinical psychology & psychotherapy*, *20*(5), 456-464.

Kessler, R. C., Andrews, G., Colpe, L. J., Hiripi, E., Mroczek, D. K., Normand, S. L., . . . Zaslavsky, A. M. (2002). Short screening scales to monitor population prevalences and trends in non-specific psychological distress. *Psychological medicine, 32*(6), 959-976.

Kessler, R. C., Barker, P. R., Colpe, L. J., Epstein, J. F., Gfroerer, J. C., Hiripi, E., ... & Zaslavsky, A. M. (2003). Screening for serious mental illness in the general population. *Archives of general psychiatry*, *60*(2), 184-189.

Kroenke, K., Spitzer, R. L., & Williams, J. B. (2001). The PHQ-9: validity of a brief depression severity measure. *Journal of general internal medicine*, *16*(9), 606-613.

Kroenke K, Wu J, Yu Z, Bair MJ, Kean J, Stump T, Monahan PO. Patient Health Questionnaire Anxiety and Depression Scale: Initial Validation in Three Clinical Trials. *Psychosom Med.* 2016 Jul-Aug;78(6):716-27.

Kroenke K, Baye F, Lourens SG. Comparative validity and responsiveness of PHQ-ADS and other composite anxiety-depression measures. J Affect Disord. 2019 Mar 1;246:437-443.

Lace, J. W., Greif, T. R., McGrath, A., Grant, A. F., Merz, Z. C., Teague, C. L., & Handal, P. J. (2019). Investigating the factor structure of the K10 and identifying cutoff scores denoting nonspecific psychological distress and need for treatment. *Mental Health & Prevention*, *13*, 100-106.

Lamers, L. M., Jonkers, C. C. M., Bosma, H., Penninx, B. W. J. H., Knottnerus, J. A., & van Eijk, J. T. (2008). Summed score of the Patient Health Questionnaire-9 was a reliable and valid method for depression screening in chronically ill elderly patients. *Journal of Clinical Epidemiology*, *61*(7), 679-687. https://doi.org/10.1016/j.jclinepi.2007.07.018

Löwe, B., Decker, O., Müller, S., Brähler, E., Schellberg, D., Herzog, W., & Herzberg, P. Y. (2008). Validation and standardization of the Generalized Anxiety Disorder Screener (GAD-7) in the general population. *Medical care*, 266-274.

Manea, L., Gilbody, S., & McMillan, D. (2012). Optimal cut-off score for diagnosing depression with the Patient Health Questionnaire (PHQ-9): a meta-analysis. *Cmaj*, *184*(3), E191-E196.

McGinty, E. E., Presskreischer, R., Han, H., & Barry, C. L. (2020). Psychological distress and loneliness reported by US adults in 2018 and April 2020. *Jama*, *324*(1), 93-94.

Murray, L. K., Dorsey, S., Bolton, P., Jordans, M. J., Rahman, A., Bass, J., & Verdeli, H. (2011). Building capacity in mental health interventions in low resource countries: an apprenticeship model for training local providers. *International Journal of Mental Health Systems*, *5*(1), 1-12.

Noppe, G., de Rijke, Y. B., Dorst, K., van den Akker, E. L., & van Rossum, E. F. (2015). LC-MS/MS-based method for long-term steroid profiling in human scalp hair. *Clinical endocrinology*, *83*(2), 162-166.

OECD, "Secretary General Angel Gurría's Statement for the G20 Videoconference Summit on Covid-19," news release, 2020, https://read.oecdilibrary.org/view/?ref=126_1264455ofyod1xpv&title=SecretaryGeneralAngelGurriaStatementforthe20_VideoconferenceSummitonCOVID19.

Ogińska-Bulik, N., Juczyński, Z., Lis-Turlejska, M., Merecz-Kot, D. (2018). Polish adaptation of the PTSD checklist for DSM-5 – PCL-5. A preliminary communication. *Przegląd Psychologiczny*, *61*(2), 287-291.

Okruszek L, Aniszewska-Stańczuk A, Piejka A, Wiśniewska M, Żurek K. Safe but lonely? Loneliness, mental health symptoms and COVID-19 [Internet]. PsyArXiv; 2020 Apr [cited 2020 May 28].

Price M, Szafranski DD, van Stolk-Cooke K, Gros DF. Investigation of abbreviated 4 and 8 item versions of the PTSD Checklist 5. Psychiatry Res. 2016 May 30;239:124-30. doi: 10.1016/j.psychres.2016.03.014. Epub 2016 Mar 8.

Public Health England. (2020). Disparities in the risk and outcomes of COVID-19.

Purgato, M., Carswell, K., Acarturk, C., Au, T., Akbai, S., Anttila, M., ... & Van Ommeren, M. (2019). Effectiveness and cost-effectiveness of Self-Help Plus (SH+) for preventing mental disorders in refugees and asylum seekers in Europe and Turkey: study protocols for two randomised controlled trials. *BMJ open*, *9*(5), e030259.

Rahman, A., Hamdani, S. U., Awan, N. R., Bryant, R. A., Dawson, K. S., Khan, M. F., ... & Van Ommeren, M. (2016a). Effect of a multicomponent behavioral intervention in adults impaired by psychological distress in a conflict-affected area of Pakistan: a randomized clinical trial. *Jama*, *316*(24), 2609-2617.

Rahman, A., Riaz, N., Dawson, K. S., Hamdani, S. U., Chiumento, A., Sijbrandij, M., ... & Farooq, S. (2016b). Problem Management Plus (PM+): Pilot trial of a WHO transdiagnostic psychological intervention in conflict-affected Pakistan. *World Psychiatry*, *15*(2), 182.

Reijnen, A., Geuze, E., & Vermetten, E. (2015). The effect of deployment to a combat zone on testosterone levels and the association with the development of posttraumatic stress symptoms: a longitudinal prospective Dutch military cohort study. Psychoneuroendocrinology, 51, 525-533.

Russell, E., Koren, G., Rieder, M., & Van Uum, S. (2012). Hair cortisol as a biological marker of chronic stress: Current status, future directions and unanswered questions. Psychoneuroendocrinology, 37(5), 589–601. https://doi.org/10.1016/j.psyneuen.2011.09.009

Rutter, L. A., & Brown, T. A. (2017). Psychometric properties of the generalized anxiety disorder scale-7 (GAD-7) in outpatients with anxiety and mood disorders. *Journal of psychopathology and behavioral assessment*, *39*(1), 140-146.

Schnurr P. P., Vielhauer M. J., Weathers F. W. (1995). Brief Trauma Interview. Unpublished.

Sijbrandij M, Horn R, Esliker R, O'May F, Reiffers R, Ruttenberg L, Stam K, de Jong J, Ager A. The Effect of Psychological First Aid Training on Knowledge and Understanding about Psychosocial Support Principles: A Cluster-Randomized Controlled Trial. Int J Environ Res Public Health. 2020 Jan 11;17(2):484. doi: 10.3390/ijerph17020484.

Singer, J. D., Willett, J. B., & Willett, J. B. (2003). *Applied longitudinal data analysis: Modeling change and event occurrence*. Oxford university press.

Ślusarska, B. J., Nowicki, G., Piasecka, H., Zarzycka, D., Mazur, A., Saran, T., & Bednarek, A. (2019). Validation of the Polish language version of the Patient Health Questionnaire-9 in a population of adults aged 35–64. *Annals of Agricultural and Environmental Medicine*, *26*(3), 420-424.

Spivak, B., Maayan, R., Mester, R., & Weizman, A. (2003). Plasma testosterone levels in patients with combat-related posttraumatic stress disorder. Neuropsychobiology, 47(2), 57-60.

Smits, N., Smit, F., Cuijpers, P., & De Graaf, R. (2007). Using decision theory to derive optimal cut-off scores of screening instruments: an illustration explicating costs and benefits

of mental health screening. *International journal of methods in psychiatric research*, *16*(4), 219-229.

Spitzer, R. L., Kroenke, K., Williams, J. B., & Löwe, B. (2006). A brief measure for assessing generalized anxiety disorder: the GAD-7. *Archives of internal medicine*, *166*(10), 1092-1097.

Staufenbiel, S. M., Penninx, B. W., de Rijke, Y. B., van den Akker, E. L., & van Rossum, E. F. (2015). Determinants of hair cortisol and hair cortisone concentrations in adults. *Psychoneuroendocrinology*, *60*, 182-194.

Stalder, T., Steudte-Schmiedgen, S., Alexander, N., Klucken, T., Vater, A., Wichmann, S., Kirschbaum, C., & Miller, R. (2017). Stress-related and basic determinants of hair cortisol in humans: A meta-analysis. Psychoneuroendocrinology, 77, 261–274. https://doi.org/10.1016/j.psyneuen.2016.12.017

Steudte, S., Kirschbaum, C., Gao, W., Alexander, N., Schönfeld, S., Hoyer, J., & Stalder, T. (2013). Hair cortisol as a biomarker of traumatization in healthy individuals and posttraumatic stress disorder patients. Biological Psychiatry, 74(9), 639–646. https://doi.org/10.1016/j.biopsych.2013.03.011

Steudte-Schmiedgen, S., Kirschbaum, C., Alexander, N., & Stalder, T. (2016). An integrative model linking traumatization, cortisol dysregulation and posttraumatic stress disorder: Insight from recent hair cortisol findings. Neuroscience and Biobehavioral Reviews, 69, 124–135. https://doi.org/10.1016/j.neubiorev.2016.07.015

Stickley, A., & Koyanagi, A. (2016). Loneliness, common mental disorders and suicidal behavior: Findings from a general population survey. *Journal of Affective Disorders*, *197*, 81-87.

Sulaiman-Hill, C. M., & Thompson, S. C. (2010). Selecting instruments for assessing psychological wellbeing in Afghan and Kurdish refugee groups. BMC Research Notes, 3, 237. https://doi.org/10.1186/1756-0500-3-237

Suri, H. (2011). Purposeful sampling in qualitative research synthesis. *Qualitative research journal*.

Thomson, R. M., & Katikireddi, S. V. (2018). Mental health and the jilted generation: Using age-period-cohort analysis to assess differential trends in young people's mental health

following the Great Recession and austerity in England. *Social Science & Medicine*, *214*, 133-143.

Tjak, J. G., Davis, M. L., Morina, N., Powers, M. B., Smits, J. A., & Emmelkamp, P. M. (2015). A meta-analysis of the efficacy of acceptance and commitment therapy for clinically relevant mental and physical health problems. *Psychotherapy and Psychosomatics*, *84*(1), 30-36.

Tol, W. A., Leku, M. R., Lakin, D. P., Carswell, K., Augustinavicius, J., Adaku, A., ... & van Ommeren, M. (2020). Guided self-help to reduce psychological distress in South Sudanese female refugees in Uganda: a cluster randomised trial. *The Lancet Global Health*, *8*(2), e254-e263.

United Nations (2020), "Policy Brief: Covid-19 and the Need for Action on Mental Health."

Lotte van Dammen, I. D., de Rooij, S. R., Behnsen, P. M., & Huizink, A. C. (2020). Sex-specific associations between person and environment-related childhood adverse events and levels of cortisol and DHEA in adolescence.

Van Praag DLG, Fardzadeh HE, Covic A, Maas AIR, von Steinbüchel N (2020) Preliminary validation of the Dutch version of the Posttraumatic stress disorder checklist for DSM-5 (PCL-5) after traumatic brain injury in a civilian population. *PLoS ONE 15*(4): e0231857. https://doi.org/10.1371/ journal.pone.0231857

Van Spijker, B. A., Batterham, P. J., Calear, A. L., Farrer, L., Christensen, H., Reynolds, J., & Kerkhof, A. J. (2014). The Suicidal Ideation Attributes Scale (SIDAS): Community-based validation study of a new scale for the measurement of suicidal ideation. *Suicide and Life-Threatening Behavior*, *44*(4), 408-419.

van Straten, A., Hill, J. J., Richards, D., & Cuijpers, P. (2014). Stepped care treatment delivery for depression: a systematic review and meta-analysis.

van 't Hof E, Heim E, Abi Ramia J, Burchert S, Cornelisz I, Cuijpers P, El Chammay R, Harper Shehadeh M, Noun P, Smit F, van Klaveren C, van Ommeren M, Zoghbi E, Carswell K. (2021). Evaluating the Effectiveness of an E-Mental Health Intervention for People Living in Lebanon: Protocol for Two Randomized Controlled Trials. *JMIR Res Protoc;10*(1):e21585. doi: 10.2196/21585.

Van Vliet, I. M., & De Beurs, E. (2007). The MINI-International Neuropsychiatric Interview. A brief structured diagnostic psychiatric interview for DSM-IV en ICD-10 psychiatric disorders. Tijdschrift voor psychiatrie, 49(6), 393-397.

Weathers, F. W., Litz, B. T., Keane, T. M., Palmieri, P. A., Marx, B. P., & Schnurr, P. P. (2013). *The PTSD Checklist for DSM-5 (PCL-5). Scale available from the National Center for PTSD at www.ptsd.va.gov.*

WHO (2010). Measuring Health and Disability: Manual for WHO Disability Assessment Schedule WHODAS 2.0. (T. B. Üstun, N. Kostansjek, S. Chatterji, & J. Rehm, Eds.). Geneva: WHO.

WHO (2011). Psychological first aid: Guide for field workers. Geneva: WHO.

WHO (2016). Problem Management Plus (PM+): individual psychological help for adults impaired by distress in communities exposed to adversity (Generic field-trial version 1.0). Geneva: WHO.

WHO (2017). Scalable psychological interventions for people in communities affected by adversity: a new area of mental health and psychosocial work at WHO. Geneva: WHO.

WHO (2020a). Doing what matters in times of stress: an illustrated guide. Geneva: WHO.

WHO (2020b). Fact sheet: Vulnerable populations during COVID-19 response - Addressing the mental health needs of vulnerable populations.

Wu, Y., Li, S., Hu, K., & Yang, J. (2021). Evidence of the Moderating Role of Hair Cortisol and Hair Cortisone in the Relationship Between Work Stress and Depression Symptoms Among Chinese fishermen. *Journal of Affective Disorders*.

Zhang, Y., Shen, J., Zhou, Z., Sang, L., Zhuang, X., Chu, M., ... & Lian, Y. (2020). Relationships among shift work, hair cortisol concentration and sleep disorders: a cross-sectional study in China. *BMJ open*, *10*(11), e038786.
