# Supplementary material for: Effectiveness of a scalable, remotely delivered stepped-care intervention for psychological distress among Polish migrant workers in the Netherlands: The RESPOND randomised controlled trial
Source: PLoS Med. 2026 Aug 17;23(8):e1005197. doi: 10.1371/journal.pmed.1005197 (PMC13480649; doi:10.1371/journal.pmed.1005197)
Supplement: Supplementary file 2 [file pmed.1005197.s007.pdf]

RESPOND

Statistical Analysis Protocol, version 4.1

This project has received funding from the European Union's Horizon 2020 research and innovation programme Societal Challenges under Grant Agreement No 101016127.

RESPOND: Improving the Preparedness of Health Systems to Reduce Mental Health and Psychosocial Concerns Resulting from the COVID-19 Pandemic

Project Acronym: RESPOND

Grant Agreement No 101016127

Deliverable Information	
**Project start date**	01/12/2020
**Duration of the project**	3 years (until 30/11/2023)
**Date**	20-12-2022
**Work package**	5
**Keywords**	Statistical protocol / study protocol
**Version**	4.1
**Reviewer**	Rinske Roos, Anke Witteveen, Marit Sijbrandij
**Authors**	Federico Tedeschi

The opinions expressed in this document reflect only the author's view and in no way reflect the European Commission's opinions. The European Commission is not responsible for any use that may be made of the information it contains.

PU=Public, CO=Confidential, only for members of the consortium (including the Commission Services), CI=Classified, as referred to in Commission Decision 2001/844/EC.

TABLE OF CONTENTS

1 Introduction 5
  1.1 Background and rationale 5
  1.2 Objectives 5
2 Study method 5
  2.1 Trial design 5
  2.2 Randomization 6
  2.3 Sample size 6
  2.4 Framework 6
  2.5 Statistical interim analyses and stopping guidance 6
  2.6 Timing of final analysis 6
  2.7 Timing of outcome assessments 7
3 Statistical Principles 7
  3.1 Confidence intervals and p-values 7
  3.2 Adherence and protocol deviations 7
  3.3 Analysis populations 7
4 Trial Population 8
  4.1 Screening data 8
  4.2 Eligibility 8
    4.2.1 Inclusion criteria: 8
    4.2.2 Exclusion criteria: 9
  4.3 Recruitment 9
  4.4 Withdrawal of individual participants 10
  4.5 Baseline participant characteristics 10
5 Analysis 10
  5.1 General approach 10
  5.2 Outcome definitions 11
    5.2.1 Primary outcome 11
    5.2.2 Secondary outcomes 11
    5.3 Other measures 13
  5.4 Analysis methods 14
    5.4.1 Primary analysis 14
    5.4.2 Secondary analyses of the primary outcome measure 14
    5.4.3. Mediation analysis 15
    5.4.4 Additional exploratory analyses 15
  5.5 Missing data 15

5.6 Additional analyses......15
5.7 Harms......15
5.8 Statistical software......16
References......16

1 INTRODUCTION

1.1 BACKGROUND AND RATIONALE

The ongoing COVID-19 pandemic has a major and potentially long-lasting effect on mental health and wellbeing across populations worldwide. Vulnerable groups, such as asylum seekers, refugees and migrants, are disproportionally affected by the COVID-19 pandemic. There is a high need for psychosocial interventions that can target the most prevalent mental health problems as a result of the COVID-19 pandemic, addressing the needs of many people in a way that maximizes the use of resources. The World Health Organization (WHO) has developed two scalable, low-intensity psychological interventions: Doing What Matters in times of stress (DWM; a self-help intervention) and Problem Management Plus (PM+; a face-to-face intervention). DWM and PM+ can be delivered by paraprofessionals, are applicable to a variety of mental health problems (depression, anxiety and PTSD), and can be adapted to different populations, cultures and languages. Both DWM and PM+ have been proven to be effective on their own. In this study, DWM and PM+ will be combined into a stepped-care intervention. This study is part of the larger EU H2020-RESPOND project, which aims to improve the preparedness of the European mental health care system in the face of future pandemics. RESPOND includes four randomized controlled trials (RCTs) that will assess the effectiveness of DWM and PM+ in different target population groups.

1.2 OBJECTIVES

The main objective is to evaluate the implementation and (cost-)effectiveness of the culturally and contextually adapted DWM/PM+ stepped-care programs amongst Polish migrant workers living in the Netherlands during the COVID-19 pandemic in terms of mental health outcomes, resilience, wellbeing, health inequalities, and costs to health systems and society. The main hypothesis is that the stepped-care DWM/PM+ intervention together with psychological first aid (PFA) in addition to care-as-usual (CAU) will be more effective in decreasing psychological distress and symptoms of mental health problems than PFA and CAU alone. The primary outcome will be the combined sum score of depression and anxiety symptoms of the PHQ-9 and GAD-7, respectively, at the two-month follow-up. Further objectives will be the identification of (a) barriers and facilitators to treatment engagement and adherence and (b) opportunities for scaling up the implementation of the DWM/PM+ intervention within the existing health care system in the Netherlands.

2 STUDY METHOD

2.1 TRIAL DESIGN

RESPOND is designed as a pragmatic implementation trial with a single-blinded, randomized, parallel-group design. The final phase of the trial will consist of a qualitative process evaluation with individual interviews and focus group discussions (FGDs). This evaluation will be conducted through semi-structured interviews with key informants, including DWM/PM+ participants (n = 20), family members/close persons of participants (n = 12), and stakeholders (e.g. (mental) health practitioners, policy makers). DWM/PM+ participants will consist of participants who completed DWM and improved, who completed PM+ and improved, who completed PM+ and did not improve, who dropped out during DWM, and who dropped out during PM+. Additionally, a focus group discussion will be conducted with helpers and trainers/supervisors (n = 6–8).

2.2 RANDOMIZATION

After screening (T0) and the baseline (T1) assessment, participants will be randomized to either the treatment group or the control group, with an equal probability of assignment to each group (allocation ratio 1:1). The trial is a single-blind RCT, in which research assistants involved in participant screening and follow-up contact are kept blind to treatment allocation where possible. Randomization will be carried out through computerized software (Castor, 2019) using permuted blocks of unequal size (4, 6). After inclusion, randomization will be initiated in Castor by a member of the research team who is not involved in participant assessment or intervention delivery. If participants are randomized into the treatment group, they will be allocated to a DWM helper. The allocated helper will contact the participant by email or phone to set up a time and date for a first acquaintance telephone call before the intervention starts. During this call, the weekly support calls of the DWM helper will be discussed and planned. If participants still reports elevated psychological distress (K10 > 15.9) at T2, they will be contacted by a PM+ helper (who may be the same helper as for DWM) to plan five consecutive tele-conferencing meetings of 60 minutes with the participant.

2.3 SAMPLE SIZE

Based on prior studies on a PM+ intervention (Bryant et al., 2017; Rahman et al., 2016), we aim to detect a small to medium Cohen's *d* effect size of 0.3 on the primary composite outcome PHQ-ADS (Kroenke et al., 2016; 2019) at 2 months post-treatment. The PHQ-ADS is the combined sum score of depression and anxiety symptoms of the PHQ-9 and GAD-7, respectively, and has shown good internal consistency (α = .88 to .92) (Kroenke et al., 2016; 2019). A power calculation for a repeated measurement design suggests a minimum sample size of N=74 per group (power=0.95, alpha=0.05, two-sided, rho=0.9) to identify an effect at the time of interest. Taking into account 30% attrition, we aim to include a total number of 212 participants (106 in the stepped-care DWM/PM+ treatment group (with PFA and CAU) and 106 in the PFA and CAU control group).

2.4 FRAMEWORK

RESPOND is a superiority trial: the hypothesis is that stepped-care DWM/PM+ with PFA and CAU is superior to PFA and enhanced CAU in lowering the severity of anxiety and depression symptoms. We thus expect migrant workers in the DWM/PM+ intervention arm to report an improvement in anxiety, depression and PTSD symptoms, as well as in resilience, and quality of life, and to incur in lower health care costs than the control arm.

2.5 STATISTICAL INTERIM ANALYSES AND STOPPING GUIDANCE

Interim analyses will be considered if safety concerns arise during the trial.

2.6 TIMING OF FINAL ANALYSIS

Outcomes will be analysed according to the scheduled assessment timepoints.

2.7 TIMING OF OUTCOME ASSESSMENTS

Baseline assessment (T1) will be performed at the start of week 1. Outcomes will be collected at T2 (week 7), T3 (week 13) and T4 (week 21). In the control group, T2 will be scheduled 42 days after completion of baseline and T3 42 days after completion of T2. In the intervention group, T2 will be scheduled 6 weeks after participants receive access to DWM, and T3 will be scheduled 1 week after completion of the last PM+ session. T4 will be scheduled 140 days after completion of baseline in both groups. If, in the intervention group, fewer than 14 days remain before the scheduled T4 assessment, T3 will not be sent or will be cancelled. Thus, while T2, T3, and T4 correspond approximately to weeks 7, 13, and 21, respectively, some variability in realized timing may occur in practice.

3 STATISTICAL PRINCIPLES

3.1 CONFIDENCE INTERVALS AND P-VALUES

The conventional 0.05 level will be adopted for statistical significance, and 95% confidence interval will be reported.

3.2 ADHERENCE AND PROTOCOL DEVIATIONS

Adherence will be defined based on completion of at least 3 modules for DWM participants and attendance of at least 4 sessions for PM+ participants, with the number of sessions considered as the extent of exposure. Due to the pragmatic nature of the trial, protocol deviations will be described descriptively where relevant and will not form the basis of a separate formal analysis.

3.3 ANALYSIS POPULATIONS

All primary and secondary analyses will be performed on an intention-to-treat (ITT) basis. The ITT population will consist of all participants randomly assigned to either study group with at least data on the baseline assessment available. In order to check the robustness of results, all outcomes will be additionally analysed using a per protocol (PP) approach, that will include only DWM participants clicking through all of the content of at least 3 modules (regardless of whether or not they engage in calls/messages with the helper) and PM+ participants attending at least 4 sessions. Control group participants will be analyzed as randomized. The analysis of the PP population will be used for sensitivity purposes only. If less than 5% of participants does not receive the allocated intervention according to the study protocol, the PP analysis will not be performed.

4 TRIAL POPULATION

4.1 SCREENING DATA

Screening (T0)
Following informed consent, participants will be invited to complete step 1 of the first assessment: screening. Screening consists of using several (self-administered) measurement instruments to see if people meet the inclusion criteria (6.2 Inclusion criteria). Also, specific questions are asked to check whether participants should be excluded because of fulfilling exclusion criteria.

- **Screening for inclusion criteria**
  In order to be included in the study, participants need to score above 15.9 on the Kessler Psychological Distress Scale (K10), a 10-item screening questionnaire for common mental disorders (Kessler et al., 2002). This instrument will be self-administered and this way literacy is immediately accounted for. Because DWM consists of online materials with text, it is important that people are able to read. If indicated, they will be referred to external specialists for potential follow-up. More detailed explanations of all measures are described under *'Measurement Instruments'.*

- **Screening for exclusion criteria**
  If individuals meet inclusion criteria, they will be screened for exclusion criteria (6.3 Exclusion criteria). If they meet one or more exclusion criteria they will be excluded and referred for appropriate treatment and support. We will assess whether individuals: (a) plan to move abroad within the next 6 months; (b) have plans to end their life or are in acute need of protection (see *Measurement Instruments)*; c) currently use psychotropic medication of which the dose has been changed in the past 2 months or currently receive specialized psychological treatment and d) suffer from a severe mental disorder (e.g., psychotic disorders, substance-dependence) or severe cognitive impairment (e.g., severe intellectual disability or dementia) based on questions and observations during screening contact by telephone or videoconference (WHO, 2016, pp. 87).

- When participants are not selected for the trial because they score below the cut-off scores for the K10 or when they meet the exclusion criteria as described in Exclusion Criteria, they will immediately be provided feedback e.g. on the screening outcomes (including K10 score) and an explanation why they are not eligible for the study (p. 86 of the PM+ intervention manual; WHO, 2016). When participants are excluded because of an imminent suicide risk, expressed acute needs/protection risks (for example, a young woman who expresses that she is at acute risk of being assaulted or killed) or observed (suspicion of) severe mental disorders or severe cognitive impairment they will be referred for appropriate treatment and support such as their general practitioner, and/or a mental health specialized support or to local social service provision, depending upon their clinical characteristics and needs. If the participant agrees to be referred, the assessment results will be provided to their general practitioner or the treating mental health professional with permission of the participant.

4.2 ELIGIBILITY

4.2.1 Inclusion criteria:

- *18 years or older;*
- *Living in the Netherlands as labour migrant;*
- *Having elevated levels of psychological distress (Kessler Psychological Distress Scale (K10) >15.9).*

- *Sufficient mastery (written and spoken) of one of the languages the DWM/PM+ intervention is being delivered in (e.g. English, Polish);*
- Having access to an electronic device with internet access and webcam to follow the intervention;
- *Oral and written informed consent before entering the study.*

4.2.2 Exclusion criteria:

- *Planning to permanently move abroad, e.g. back to their home country or to another country, before the last quantitative assessment at 2 months after PM+ (T4);*
- *Having acute medical conditions (requiring hospitalization);*
- *Imminent suicide risk, or expressed acute needs or protection risks that require immediate follow-up;*
- *Having a severe mental disorder (e.g. psychotic disorders, substance-dependence);*
- *Having severe cognitive impairment (e.g. severe intellectual disability or dementia);*
- *Currently receiving specialized psychological treatment (e.g. EMDR, CBT);*
- *In case of current psychotropic medication use: not being on a stable dose for at least 2 months, or having had a change in dosage over the past 2 months.*

4.3 RECRUITMENT

Eligible trial participants will be adults (18 years or above) Polish migrant workers meeting the inclusion and exclusion criteria described in paragraph 4.2. The recruitment strategy for the RCT will be pragmatic and will mainly involve targeted (social) media strategies, snowballing, as well as collaboration with community organizations and local institutions that provide services for migrant workers (e.g. municipalities, NGOs, health care providers, and/or language courses). The qualitative evaluation will include a purposive sampling method called maximum variation sampling. In this sampling method, the sample is selected based on variations of key characteristics (Suri, 2011). For participants in the treatment group, relevant variables will include gender, age, and intervention completion status (drop-out or completed). Selection of DWM and PM+ helpers will also be based on maximum variation; relevant variables will include gender, age, and the type of intervention (DWM and/or PM+) provided. Family members or close persons of participants, DWM and PM+ helpers, mental health specialists/supervisors involved in the trial, and decision makers from key stakeholder groups will be approached for participation in the qualitative evaluation through participant permission or available professional contacts.

Informed consent procedure
Before being enrolled in the study, participants will be informed about the nature and scope of the study in a form understandable to them. Participation will be completely voluntary to prevent biased responding – either exaggerating or minimizing problems in the belief that this may help participants obtaining secondary advantages. If a potential participant wants to participate, a research assistant will contact the potential participant by phone and will explain the research to the potential study participant and will provide the information document to the participant.

Persons who decide to participate in the trial will be asked to complete a written consent form to check for eligibility (after a minimum consideration time of one week). If participants meet the eligibility criteria (K10 >15.9 and not meet any exclusion criteria), they can participate in the trial. We will inform participants about the reason why they can or cannot participate in the RCT. In order to participate in the trial, participants will complete an informed consent form including optional checkbox items covering the recording of DWM support calls and PM+ sessions, participation in the qualitative interviews, and provision of a hair sample. Giving consent for the recordings, qualitative interviews, and/or hair sample is not a condition for participating in the study. DWM support calls will be audio recorded and PM+ sessions will be video recorded, and these recordings will only be used for fidelity assessments and supervision.

Participants are allowed to withdraw from the study at any time after they have given their written consent.

4.4 WITHDRAWAL OF INDIVIDUAL PARTICIPANTS

Participants can leave the study at any time for any reason if they wish to do so without any consequences. The research team can decide to withdraw a participant from the intervention for urgent medical reasons, e.g. imminent suicide risk. When, during calls with DWM/PM+ helpers, participants show deterioration with imminent suicidal plans, then the helper will discuss this immediately with one of the DWM/PM+ supervising mental health specialists who will inform the research team. Also, when there is clear suspicion of worsening of (severe) mental health problems, participants will be asked to contact their general practitioner for a referral to specialized mental health treatment.

No new participants will be included for each withdrawn participant. In our power calculation for the sample size, we have taken into account 30% attrition.

If a participant decides to withdraw from the study, the research team will ask for the reason. It will be enquired whether the participant wishes to withdraw from the study or from a specific time point only and so whether the participant can be re-contacted at a later time. Withdrawal from the study will have no effect on any care received. Participants who leave the study for medical or safety reasons will be followed up as appropriate until the situation has stabilized or appropriate care has been arranged.

4.5 BASELINE PARTICIPANT CHARACTERISTICS

If participants meet the eligibility criteria and score above the cut-off of the K10, they will continue with the baseline assessment. This step includes administration of questionnaires about socio-demographic characteristics and: the Patient Health Questionnaire-9 (PHQ-9), Generalized Anxiety Disorder-7 (GAD-7), the PTSD Checklist for DSM-5 (PCL-5), the Brief Trauma Questionnaire (BTQ), COVID-19-related questions, the Mainz Inventory of Microstressors (MIMIS), the Positive Appraisal Style Scale – content focused (PASSc), Checklist for Quality of Life (EQ-5D-5L), and the Client Service Receipt Inventory (CSRI). In addition, participants may optionally provide a hair sample at baseline. Information on treatment fidelity, satisfaction and acceptability of the intervention programme, and implementation indicators (such as reach, dose, resource use, and intervention-related development and implementation costs) will also be collected during the study.

5 ANALYSIS

5.1 GENERAL APPROACH

No formal masking of the statistical analysis is planned. Moreover, the person conducting the statistical analysis will not be involved in determining participants' eligibility, administering the intervention, measuring the outcomes, or entering data.

Three endpoints will occur during the study. The first one will happen 1 week post-DWM (T2), the second one 1 week post-PM+ (T3), and the third one at follow-up (T4, week 21). We will not conduct post-intervention assessments immediately after completion of the intervention, in order to give participants some time to practice the strategies learned. In practice, some variability in realized timing may occur. Regarding the primary endpoint, T4 will be scheduled 140 days after completion of baseline in both groups.

5.2 OUTCOME DEFINITIONS

5.2.1 Primary outcome

The primary outcome will be the average PHQ-ADS score, i.e. the combined sum score of depression and anxiety symptoms of the PHQ-9 and GAD-7, respectively, measured at weeks 7, 13, and 21. Week 21 (T4) will be the primary endpoint. It has shown good internal consistency (α = .88 to .92) (Kroenke et al., 2016; 2019).

5.2.2 Secondary outcomes

The secondary clinical outcomes of our study will be: depression (PHQ-9) and anxiety (GAD-7) separately; symptoms of PTSD (PCL-5), measured at weeks 7, 13 and 21. In addition, prespecified measures related to resilience and health economic evaluation, including health-related quality of life (EQ-5D-5L) and service use collected using adapted versions of the Client Service Receipt Inventory (CSRI), will be collected for separate planned analyses. Rates of participants withdrawing from the study at weeks 7, 13 and 21 will also be assessed.

PHQ-9: depression (PHQ-9; subscale of PHQ-ADS)
Depressive symptoms during the past two weeks will be measured using the Patient Health Questionnaire depressive module. It asks how often someone was bothered by each of the nine DSM-5 criteria and scores answers on a four-point Likert scale ranging from 0 (not at all) to 3 (nearly every day) (Kroenke et al. , 2001). In addition to the nine items, the PHQ-9 asks: “If you checked off *any* problems, how *difficult* have these problems made it for you to do your work, take care of things at home, or get along with other people?”, which is to be answered with “Not difficult at all”, “Somewhat difficult”, “Very difficult”, or “Extremely difficult”. For the current study, we will examine changes in caseness in depression. We will use a cut-off score of 10, which has been found to be a valid cut-off point for diagnosis (Manea et al., 2012).
The PHQ-9 has been translated to and is available in many languages (see https://www.phqscreeners.com/), amongst which are English and Italian. The PHQ-9 has been found to be a reliable and valid instrument to measure depressive severity. Furthermore, due to its brevity, PHQ-9 is a useful instrument for usage in a clinical or research setting (Kroenke et al., 2001).

GAD-7: anxiety symptoms (GAD-7; subscale of PHQ-ADS)
The Generalized Anxiety Disorder (GAD-7) questionnaire is a seven-item, self-report anxiety questionnaire which assesses the degree to which the participant has been bothered by feeling nervous, anxious or on edge over the last two weeks. Items also include other generalized anxiety symptoms such as being unable to stop worrying about multiple things, having trouble relaxing or sitting still, feeling irritable and being afraid of something bad happening at all times (Spitzer et al., 2006). Items are scored from 0 to 3, respectively for experiencing symptoms ‘not at all’, for ‘several days’, for ‘more than half the days’ and for ‘nearly every day’. The total score ranges from 0 to 21. Cut-off points for mild, moderate and severe anxiety, are scores of 5, 10 and 15, respectively (Spitzer et al., 2006). A score of 10 has been identified as the optimal cut-off score to balance specificity and sensitivity (Spitzer et al., 2006).
The GAD-7 has been translated to and is available in many languages (see https://www.phqscreeners.com/), amongst which are English and Italian. The GAD-7 has been found to be a valid and reliable instrument in both the psychiatric (Kertz et al., 2013; Rutter & Brown, 2017) and the general population (Löwe et al., 2008; Hinz et al., 2017).

PCL-5: PTSD Symptoms
Posttraumatic stress disorder (PTSD) symptoms during the past week according to the DSM-5 PTSD diagnosis will be measured using the PTSD Checklist for DSM-5 (PCL-5) (Weathers et al., 2013). A shortened 8-item version of the original PCL-5 (a 20-item checklist which correspond with the 20 DSM-5 PTSD symptoms) will be used. Items are rated on a 0-4 scale. Added up, the maximum severity score is 32. Higher scores indicate higher symptomatology. The original 20-item PCL-5 exists amongst others, in English (Weathers et al., 2013).
Since populations in the trials might not all be severely traumatized, we will use the abbreviated 8-item version. In a comparison of two abbreviated version, i.e., the 4-item and 8-item versions of the PCL-5, the PCL-5 8 item version showed a strong correlation with the total scale, greater internal consistency, and allowed for sufficient variability in

participant response. Internal consistency of the 8-item version was α 0.90 (comparable to total PCL-5 20-item version) and accounted for 94.1% (R = 0.97) of the variance in the total PCL-5. There were no significant differences in the sensitivity and specificity between the total 20-item PCL-5 scale and the 8-item scale (Price et al., 2016).

EQ-5D-5L: quality of life

The EQ-5D-5L measures quality of life and consists of two parts, the EQ-5D and the EQ VAS. Part 1, the EQ-5D, rates the level of impairment across five dimensions: mobility, self-care, usual activities, pain/discomfort, and anxiety/depression. Each dimension has 5 levels: no problems, slight problems, moderate problems, severe problems and extreme problems. The EQ-5D-5L is an adapted version of the EQ-5D(-3L), which only had three response options for each dimensions and was therefore thought to not sufficiently capture milder health issues and small changes between different states of health (Herdman et al., 2011). The EQ-5D-5L has been used widely and is available in over 150 languages, also for laptop, tablet or Castor EDC (https://euroqol.org/eq-5d-instruments/eq-5d-5l-available-modes-of-administration/self-complete-for-use-in-castor-edc/). Country specific utility weights will be attached to data from the EQ-5D-5L and changes in participant quality of life years gained between intervention and control groups will be determined. Part 2, the EQ VAS, is a visual, vertical, analogue scale. The endpoints of the scale are called 'The best health you can imagine' and 'The worst health you can imagine' and the current health status of that day needs to be indicated, after which the number checked on the scale also needs to be written down.

CSRI schedule: resource use and cost of care

The Client Service Receipt Inventory (CSRI) was developed for the collection of data on service utilization (e.g. use of health system, other services, time out of employment and other usual activities, need for informal care) and related characteristics of people with mental disorders, as the basis for calculating the costs of care for mental health cost-effectiveness research. It has been used cross-culturally (Chisholm et al., 2000). It will be tailored and adapted reflecting the specific context, health system structures and target population in the Netherlands.

Resilience: Measuring mental health in relation to stressor exposure

Resilience is defined as an outcome of good mental health despite adversity (the maintenance or quick recovery of mental health during and after periods of stressor exposure, including traumatizing life events, difficult life circumstances, challenging life transitions, or physical illness; Kalisch et al., 2017). This requires relating changes in mental health to stressor exposure.

The Mainz Inventory of Microstressors (MIMIS) was recently developed to measure objective microstressors of modern life in the past 7 days (Chmitorz et al., 2020). In the Dynacore-C study (Veer et al., 2021), conducted to detect resilience factors active during the first Corona lockdown, the MIMIS was adapted in three ways. Firstly, it was shortened to reduce the burden on participants, secondly, new items were generated to capture exposure to pandemic-related stressors, and thirdly, the question was changed to cover the past 14 days (rather than 7 previously). Further adaptation was done for RESPOND. This included quantitative analysis of stressor occurrence and the variance they explain in mental health in existing data sets. The resulting lists were then qualitatively checked and new items were added based on qualitative interviews of members of the trial population of migrants, refugees, and/or asylum seekers or healthcare workers living in the trial's country, to thereby assure that population-specific stressors will also be covered. With the adapted instrument, stressor exposure is thus measured in RESPOND by a combined list of general stressors (6 items), COVID-19-specific stressors (5 items), general life events (n=3), and population-specific stressors (n=8 for WP5; n=4 for WP4). Daily stressor items (general, COVID-19, population-specific) are rated on a four-point Likert scale in which participants can indicate the frequency at which a stressor occurred, ranging from 0 (did not happen/almost never) to 3 ((nearly) every day). Life events are rated on a five-point Likert scale to indicate the impact of this event, ranging from 0 (this situation did not happen) to 4 (severe impact).

Using this data, outcome-based resilience at any time point T will be approximated by relating self-reported mental health problems (i.e. anxiety and depression) over the past 2 weeks (assessed with the PHQ-ADS) to the corresponding stressor exposure. To this end, a stressor reactivity (SR) score will be computed as an individual score against normal stressor reactivity (see Kalisch et al., 2021), where normal stressor reactivity is the regression line of average mental health problems against average stressor exposure across all time points in the trial population and one's individual SR score at any time point is the distance to the regression line. SR scores will be calculated based on the stressor exposure count that explains the most variance in mental health problems. Comparisons will be made between three different stressor exposure counts: daily stressors (general, COVID-19, population-specific), life event count, and a combined score of daily stressors and life events (combined mean z-scores of stressor counts). If the combined score

explains more variance in the PHQ-ADS, it will be used for the subsequent computation of the SR score, else the separate exposure scores will be used (resulting in two separate SR scores, expressing reactivity to daily hassles and to life events, respectively). SR scores will be computed for each time point. The inverse of the SR score is considered an approximative index of outcome-based resilience.

5.3 Other measures

In addition to the primary and secondary outcomes described above, other prespecified measures will be collected to assess resilience-related factors, traumatic experiences, and the impact of COVID-19.

Resilience factor(s): positive appraisal style scale, content-focused
Resilience factors (i.e. underlying factors that lead to resilience) may also be measured by assessing factors like optimism, positive appraisal style, perceived social support (in general and related to COVID-19), perceived self-efficacy and behavioral coping style. In RESPOND, we will assess positive appraisal style with the "Positive Appraisal Style Scale – content focused" (PASSc). The PASSc is based on positive appraisal style theory of resilience (PASTOR; Kalisch et al, 2015; Kalisch et al, 2021). The PASTOR theory conceptualises resilience as an outcome: the maintenance of mental health after stressor exposure. Positive appraisal style would therefore not be a measure of resilience, but a resilience factor. It intends to capture the underlying mechanism which leads to resilience. The PASSc is currently used in a number of longitudinal studies (Mainz Resilience Study (MARP); Longitudinal Resilience Assessment study (LORA)and several studies of the DynaMORE project). The PASSc was originally developed as a 29 items questionnaire featuring generalized positive appraisals of and attitudes towards difficulties, covering specifically the 3 main dimensions of stressor/threat appraisal - appraisal of threat magnitude/cost (relating to catastrophizing vs. trivialization), of threat probability (relating to pessimism vs. optimism), and of one's coping potential (relating to helplessness vs. overconfidence). Internal validity testing and a factor analysis resulted in a reduced list of 12 items, which is the Positive Appraisal Style Scale (PASSc). A paper (R. Kalisch and P. Petri-Romão) currently being prepared shows internal consistency $\alpha = .87$ and reliability Cronbach's $\alpha = .84$. The PASSc shows convergent validity with other underlying resilience factors as it correlates with optimism .52 (SOP-2), with stress recovery (BRS): .50, with well-being (WHO-5): .42, with trait anxiety (STAI-Y2): -.51, with neuroticism (from BFI-10): -.49. Discriminant validity is shown in low correlation with I-8 impulsivity subscales urgency, intention <=.13; with openness (from BFI-10): .17, with conscientiousness (from BFI-10): .19.

Brief Trauma Questionnaire
The BTQ is a brief self- report questionnaire that is derived from the Brief Trauma Interview (Schnurr et al., 1995). (Information about the reliability and validity of the BTI is provided in Schnurr et al., 2002). The BTQ was originally designed to assess traumatic exposure according to DSM-IV but specifically asked only about Criterion A.1 (life threat/serious injury) because of the difficulty of accurately assessing A.2 (subjective response) in a brief self- report format. Criterion A.2 has been eliminated from the PTSD diagnostic criteria in DSM- 5, so the BTQ provides a complete assessment of Criterion A. The questionnaire may be used to determine whether an individual has had an event that meets the A Criterion, or to determine the different types of Criterion A events an individual has experienced.

Impact of COVID-19 questionnaire
The COVID-19 questionnaire includes 11 questions related to the impact of COVID-19. The questionnaire is based on other COVID-19 questionnaires (Conway, Woodward & Zubrod, 2020). It may be backward translated from English to other languages and will be administered at each time-point.

Hair samples
Optional hair samples will be collected to assess biological markers of physiological stress. Approximately 100 hair strands will be cut as close as possible to the scalp at the vertex posterior, and the proximal 3 cm segment will be analysed for cumulative hair cortisol concentrations (HCC), cortisone, dehydroepiandrosterone (sulphate) (DHEA(S)), testosterone, and progesterone levels. These biomarkers provide an indication of longer-term neuroendocrine stress-related activity (Stalder et al., 2017; Staufenbiel et al., 2015). Based on an average hair growth rate of 1 cm per month, this provides an indication of physiological stress over the preceding 3 months (Wennig, 2000). Participants will be instructed to obtain the hair sample with the assistance of another person and will receive support through an

instruction video. In addition, participants will complete a brief hair questionnaire on relevant hair-specific characteristics, such as hair washing, hair treatments, and substance or medication use (e.g. corticosteroids).

Video analysis of PM+ sessions
With participant consent, video recordings of PM+ sessions will be used to examine digital markers of facial activity, voice, and movement. These may include indicators such as facial expressions (e.g. happiness, sadness, fear), speech prevalence, and head movement. Analyses will be conducted using open-source tools based on the approach described by Galatzer-Levy et al. (2020).

5.4 ANALYSIS METHODS

5.4.1 Primary analysis

The primary outcome will be summarized using number of participants (*n*), minimum and maximum; and means, standard deviations (*SD*) for normally distributed data, or medians and inter-quartile ranges for non-normally distributed data. To compare the two treatment groups at baseline Standardized Mean Difference will be calculated. The primary analysis will simultaneously assess treatment effect on the average PHQ-ADS score at each time-point. To do so, we will adopt an intention-to-treat (ITT) approach, including all randomized participants. The main conclusion of the trial will be based on the ITT analysis of the primary outcome (i.e. the effect on PHQ-ADS score at the two-month follow-up). To estimate the treatment effect for the time-points T2, T3 and T4, a linear mixed model will be employed for the analysis on PHQ-ADS, which will have time as a fixed effect, baseline measurement of PHQ-ADS as covariate, and participant as random effects. The model will include a time × treatment interaction, such that treatment effects can be estimated at each post-baseline time-point, with the effect at T4 being the primary comparison of interest. The mean difference between two treatment arms at each visit/time together with its 95% confidence interval will be derived from the mixed model.

5.4.2 Secondary analyses of the primary outcome measure

A secondary analysis of the effect of treatment on PHQ-ADS score will be conducted using the per protocol (PP) population, using the same approach as reported above. In addition, a covariate-adjusted mixed model of primary endpoint will be performed by adding pre-specified covariates at baseline (e.g., gender, age, education, prior trauma, COVID-19 related events) into the above model.

Analysis of secondary outcomes with repeated measurements
A linear mixed model as mentioned for the primary analysis (PHQ-ADS) will be carried out for analyzing each of the following clinical outcomes measured at weeks 7, 13 and 21: posttraumatic stress reactions (PCL-5), depressive symptoms (PHQ-9), generalized anxiety (GAD-7), resilience and quality of life (EQ-5D-5L).

Economic outcomes
Health economic analysis will be conducted to determine the cost effectiveness of intervention compared to care as usual at each time-point. Primary analysis will focus on the incremental cost per change in primary outcome (e.g. composite PHQ-ADS) and incremental cost per QALY gained at 2-month follow-up. This involves synthesis of data on outcomes, with intervention and compactor costs and changes in resource utilization and productivity. Between-group comparison of mean costs will be completed using standard t-test with ordinary least squares regression used for adjusted analysis, with the validity of results confirmed using bootstrapping. One way and probabilistic sensitivity analyses will be used to account for uncertainty in trial parameters; cost effectiveness planes and cost effectiveness acceptability curves will be constructed. Pseudonymised data will be sent to the London School of Economics and Political Science, partner in RESPOND under Work Package 3, for the health economics analysis.

Analysis of clinically relevant change in the primary outcome
Clinically relevant change in symptoms of anxiety and depression, as measured by the PHQ-ADS, will be assessed using criteria for reliable and clinically meaningful change. Participants will be classified over time into categories such as

recovery, improvement without recovery, deterioration, or no meaningful change, based on the Reliable Change Index and a predefined clinical cut-off. Results will be summarised by treatment group and timepoint.

All the analyses on secondary outcomes will be repeated by including pre-specified covariates at baseline (e.g., gender, age, education, prior trauma, COVID-19 related events) for both ITT and PP population.

5.4.3. Mediation analysis

We hypothesize that the effects of treatment on the primary outcome as well as on the secondary outcomes will be mediated by its effects on the resilience factor positive appraisal style.

5.4.4 Additional exploratory analyses

Exploratory analyses will be conducted to understand the relationships between traumatic events exposure (as measured by the Brief Trauma Questionnaire) and general stressor exposure (as described in the section 'resilience'). If no relationship between the two is shown, then the two exposures will be treated separately in the computation of resilience. Additionally, resilience scores based on the depression (PHQ-9) and anxiety (GAD-7) subscales of the PHD-ADS will be explored to understand the differential influence of stressor exposure on these domains.

5.5 MISSING DATA

Missing data will be treated as missing at random (MAR). No imputations of missing values will be made, as multilevel models can deal with missing data (Singer & Willett, 2003). In the case only some items are missing for a specific scale, we will perform the Corrected Item mean Substitution method (i.e. the item mean across participants weighted by the participant's mean of completed items; Huisman, 1999), using information from participants belonging to the same treatment arm for the same follow-up time (estimated values above the maximum or below the minimum admissible value will be set to maximum/minimum).

5.6 ADDITIONAL ANALYSES

In addition to the analyses reported above, an Individual-level Participant Data meta-analysis combining observations from all trials will be conducted.

5.7 HARMS

Adverse events are defined as any undesirable experience occurring to a participant during the study, whether or not considered related to the trial procedure or the stepped-care DWM/PM+ intervention. All adverse events reported spontaneously by the participant or observed by the research team will be recorded.

All AEs will be followed until they have abated, or until a stable situation has been reached.

Depending on the event, follow-up may require additional tests or medical procedures as indicated, and/or referral to the general physician or a medical specialist. SAEs need to be reported till end of study. The DWM and PM+ helpers will be supervised and receive weekly supervision by experienced mental health care professionals. If, during the course of the study, participants in the treatment group (DWM/PM+/CAU) or control group (CAU only) show severe psychiatric

symptoms (e.g., psychosis, imminent suicide risk, etc.), or any other symptoms occur that require immediate specialist treatment and follow-up, they will be referred to specialist staff (e.g. psychiatrists) for immediate follow-up. The researcher from Vrije Universiteit Amsterdam will be responsible to monitor this process and make sure the appointment has been made.

5.8 STATISTICAL SOFTWARE

All analyses will be performed using the latest version of RStudio.

REFERENCES

Ashworth M., Shepher M., Christey J., Matthews V., Wright K., Parmentier H., Robinson S. Godfrey E. (2004). A client-generated psychometric instrument: the development of “PSYCHLOPS.” *Counseling and Psychotherapy Research*; **4**(2), 27–32. https://doi.org/10.1093/astrogeo/atw101.

Bryant R. A., Schafer A., Dawson K. S., Anjuri D., Mulili C., Ndogoni L., Koyiet P., SIjbrandij M., Ulate J., Harper Shehadeh M., Hadzi-Pavlovic D., van Ommeren M. (2017). Effectiveness of a brief behavioural intervention on psychological distress among women with a history of gender-based violence in urban Kenya: A randomised clinical trial. *PloS Medicine*; **14**(8), e1002371.

Castor EDC. (2019). Castor Electronic Data Capture. [online] Available at: https://castoredc.com.

Chisholm D., Knapp M., Knudsen H., Amaddeo F., Gaite L., van Wijngaarden B. (2000). Client Socio-Demographic and Service Receipt Inventory-European Version: development of an instrument for international research. EPSILON Study 5. European Psychiatric Services: Inputs Linked to Outcome Domains and Needs. *British Journal of Psychiatry*; **39**, s28-33.

Chmitorz A., Kurth K., Mey L. K., Wenzel M., Lieb K., Tüscher O., Kubiak T., Kalisch R. (2020). Assessment of Microstressors in Adults: Questionnaire Development and Ecological Validation of the Mainz Inventory of Microstressors. *JMIR Mental Health*; **7**(2), e14566. doi: 10.2196/14566. Erratum in: *JMIR Mental Health*. 2020; **7**(5), e18626. PMID: 32130154; PMCID: PMC7063526.

Czachowski S., Seed P., Schofield P., Ashworth M. (2011). Measuring psychological change during cognitive behaviour therapy in primary care: a Polish study using 'PSYCHLOPS' (Psychological Outcome Profiles). *PLoS One*; **6**(12), e27378.

Galatzer-Levy IR, Abbas A, Yadav V, Koesmahargyo V, Aghjayan A, Marecki S, et al. Remote digital measurement of visual and auditory markers of Major Depressive Disorder severity and treatment response. medRxiv; 2020 [cited 2023 Jun 23]. p. 2020.08.24.20178004. Available from: 10.1101/2020.08.24.20178004.

Héðinsson H., Kristjánsdóttir H., Ólason D. Þ., Sigurðsson J. F. (2013). A validation and replication study of the patient-generated measure PSYCHLOPS on an Icelandic clinical population. *European Journal of Psychological Assessment*; **29**(2), 89–95.

Herdman M., Gudex C., Lloyd A., Janssen M. F., Kind P., Parkin D., Bonsel G., Badia X. (2011). Development and preliminary testing of the new five-level version of EQ-5D (EQ-5D-5L). *Quality of Life Research*; **20**(10), 1727-1736.

Hinz A., Klein A. M., Brähler E., Glaesmer H., Luck T., Riedel-Heller S. G., Kerstin W., Hilbert A. (2017). Psychometric evaluation of the Generalized Anxiety Disorder Screener GAD-7, based on a large German general population sample. *Journal of Affective Disorders*; **210**, 338-344.

Huisman J. M. E. (1999). Item nonresponse: occurrence, causes, and imputation of missing answers to test items. Leiden: DSWO Press.

Kalisch R., Müller M. B., Tüscher O. (2015). A conceptual framework for the neurobiological study of resilience. *Behavioral and Brain Sciences*; **38**, e92.

Kalisch R., Baker D., Basten U., *et al*. (2017). The resilience framework as a strategy to combat stress-related disorders. *Nature Human Behaviour*; **1**(11), 784-790.

Kalisch R., Köber G., Binder H., *et al*. (2021). The Frequent Stressor and Mental Health Monitoring-Paradigm: A Proposal for the Operationalization and Measurement of Resilience and the Identification of Resilience Processes in Longitudinal Observational Studies. *Frontiers in Psychology*; **12**, 710493. https://doi.org/10.3389/fpsyg.2021.710493.

Kertz S., Bigda-Peyton J., Bjorgvinsson T. (2013). Validity of the Generalized Anxiety Disorder-7 Scale in an acute psychiatric sample. *Clinical Psychology & Psychotherapy*; **20**(5), 456-464.

Kessler R. C., Andrews G., Colpe L. J., Hiripi E., Mroczek D. K., Normand S. L. T., Walters E. E., Zaslavsky A. M. (2002). Short screening scales to monitor population prevalences and trends in non-specific psychological distress. *Psychological Medicine*; **32**(6), 959-976.

Kroenke K., Spitzer R. L., Williams J. B. (2001). The PHQ-9: validity of a brief depression severity measure. *Journal of General Internal Medicine*; **16**(9), 606-613.

Kroenke K., Wu J., Yu Z., Bair M. J., Kean J., Stump T., Monahan P.O. (2016). Patient Health Questionnaire Anxiety and Depression Scale: Initial Validation in Three Clinical Trials. *Psychosomatic Medicine*; **78**(6), 716-727.

Kroenke K., Baye F., Lourens S. G. (2019). Comparative validity and responsiveness of PHQ-ADS and other composite anxiety-depression measures. *Journal of Affective Disorders*; **246**, 437-443.

Löwe B., Decker O., Müller S., Brähler E., Schellberg D., Herzog W., Herzberg P. Y. (2008). Validation and standardization of the Generalized Anxiety Disorder Screener (GAD-7) in the general population. *Medical care*; **46**(3), 266-274.

Manea L., Gilbody S., McMillan D. (2012). Optimal cut-off score for diagnosing depression with the Patient Health Questionnaire (PHQ-9): a meta-analysis. *CMAJ*; **184**(3), E191-E196.

Price M., Szafranski D. D., van Stolk-Cooke K., Gros D. F. (2016). Investigation of abbreviated 4 and 8 item versions of the PTSD Checklist 5. *Psychiatry Research*; **239**, 124-30.

R Core Team (2021). R: A language and environment for statistical
computing. R Foundation for Statistical Computing, Vienna, Austria.

Rahman A., Riaz N., Dawson K. S., Hamdani S. U., Chiumento A., Sijbrandij M., Minhas F., Bryant R. A., Saeed K., Mark van Ommeren M., Farooq S. (2016). Problem Management Plus (PM+): Pilot trial of a WHO transdiagnostic psychological intervention in conflict-affected Pakistan. *World Psychiatry*; **15**(2), 182-183.

Rutter L. A., Brown T. A. (2017). Psychometric properties of the generalized anxiety disorder scale-7 (GAD-7) in outpatients with anxiety and mood disorders. *Journal of Psychopathology and Behavioral Assessment;* **39**(1), 140-146.

Schnurr P. P., Vielhauer M. J., Weathers F. W. (1995). Brief Trauma Interview. Unpublished.

Schnurr P.P., Spiro A. III, Vielhauer M. J., Findler M. N., Hamblen J. L. (2002). Trauma in the lives of older men: Findings from the Normative Aging Study. *Journal of Clinical Geropsychology;* **8**, 175-187.

Singer J. D., Willett J. B. (2003). Applied longitudinal data analysis: Modeling change and event occurrence. Oxford University Press.

Spitzer R. L., Kroenke K., Williams J. B., Löwe B. (2006). A brief measure for assessing generalized anxiety disorder: the GAD-7. *Archives of Internal Medicine*; **166**(10), 1092-1097.

Stalder, T., Steudte-Schmiedgen, S., Alexander, N., Klucken, T., Vater, A., Wichmann, S., Kirschbaum, C., & Miller, R. (2017). Stress-related and basic determinants of hair cortisol in humans: A meta-analysis. Psychoneuroendocrinology, 77, 261–274.

Staufenbiel, S. M., Penninx, B. W., de Rijke, Y. B., van den Akker, E. L., & van Rossum, E. F. (2015). Determinants of hair cortisol and hair cortisone concentrations in adults. Psychoneuroendocrinology, 60, 182-194.

Suri H. (2011). Purposeful sampling in qualitative research synthesis. *Qualitative Research Journal*; **11**(2), 63-75.

Veer I. M., Riepenhausen A., Zerban M. *et al.* (2021). Psycho-social factors associated with mental resilience in the Corona lockdown. *Translational Psychiatry*; **11**, 67.

Weathers F. W., Litz B. T., Keane T. M., Palmieri P. A., Marx B. P., Schnurr P. P. (2013). The PTSD Checklist for DSM-5 (PCL-5). Scale available from the National Center for PTSD at www.ptsd.va.gov.

Wennig R. Potential problems with the interpretation of hair analysis results. Forensic Sci Int. 2000;107(1–3):5–12.

WHO (2016). Problem Management Plus (PM +): individual psychological help for adults impaired by distress in communities exposed to adversity (Generic field-trial version 1.0). Geneva: WHO.

Zellner A. (1962). An Efficient Method of Estimating Seemingly Unrelated Regressions and Tests for Aggregation Bias. *Journal of the American Statistical Association*; **57**(298), 348-368.
