# Supplementary material for: Effectiveness of a scalable, remotely delivered stepped-care intervention for psychological distress among Polish migrant workers in the Netherlands: The RESPOND randomised controlled trial
Source: PLoS Med. 2026 Aug 17;23(8):e1005197. doi: 10.1371/journal.pmed.1005197 (PMC13480649; doi:10.1371/journal.pmed.1005197)
Supplement: Supplementary file 4 [file pmed.1005197.s002.docx]

**S1 Fig.**
*(A) Mean PHQ-ADS scores over time by PM+ eligibility group (intention-to-treat) with 95% confidence intervals: eligible (n=85) and not eligible (n=10). The number of eligible participants per timepoint was: t_1_=85, t_2_=85, t_3_=71, t_4_=82. (B) Individual PHQ-ADS trajectories for not-eligible participants (n=10).* *Note.* PHQ-ADS = Patient Health Questionnaire-Anxiety and Depression Scale; PM+ = Problem Management Plus; DWM = Doing What Matters in Times of Stress.
