# Supplementary material for: Effectiveness of a scalable, remotely delivered stepped-care intervention for psychological distress among Polish migrant workers in the Netherlands: The RESPOND randomised controlled trial
Source: PLoS Med. 2026 Aug 17;23(8):e1005197. doi: 10.1371/journal.pmed.1005197 (PMC13480649; doi:10.1371/journal.pmed.1005197)
Supplement: Supplementary file 5 [file pmed.1005197.s003.docx]

**S1 Table**
*Baseline Sociodemographic, Clinical, and COVID-Related Characteristics of Participants by Randomisation Group*

| **Variables** | **Randomisation group** | |  | |
| --- | --- | --- | --- | --- |
|  | Intervention  *n*=109 | Control  *n*=109 | | Full sample  *N*=218 |
| Gender, *n (%)* |  |  | |  |
| Female | 86 (78.9) | 76 (70.4) | | 162 (74.7) |
| Male | 23 (21.1) | 30 (27.8) | | 53 (24.4) |
| Other | 0 | 2 (1.9) | | 2 (0.9) |
| Missing | 0 | 1 | | 1 |
| Age, *M (SD)* (*n* missing) (range) | 37.5 (9.1) (2 missing) (20-61) | 40.0 (9.5) (1 missing) (20-61) | | 38.8 (9.4) (3 missing) (20-61) |
| Second languages spoken (Polish excluded), *n (%)* |  |  | |  |
| English | 87 (79.8) | 77 (70.6) | | 164 (78.8) |
| Dutch | 42 (38.5) | 48 (44.0) | | 90 (43.3) |
| Russian | 13 (11.9) | 13 (11.9) | | 26 (12.5) |
| German | 5 (4.6) | 7 (6.4) | | 12 (5.8) |
| Spanish | 3 (2.8) | 2 (1.8) | | 5 (2.4) |
| Italian | 2 (1.8) | 2 (1.8) | | 4 (1.9) |
| Ukrainian | 3 (2.8) | 0 | | 3 (1.4) |
| French | 2 (1.8) | 0 | | 2 (1.0) |
| Slovak | 1 (0.9) | 1 (0.9) | | 2 (1.0) |
| Serbo-Croatian | 0 | 1 (0.9) | | 1 (0.5) |
| Missing | 1 | 9 | | 10 |
| Number of second languages spoken (Polish excluded), *n (%)* |  |  | |  |
| 0 | 15 (13.8) | 15 (13.8) | | 30 (14.4) |
| 1 | 45 (41.3) | 35 (32.1) | | 80 (38.5) |
| 2 | 33 (30.3) | 36 (33.0) | | 69 (33.2) |
| 3 | 13 (11.9) | 13 (11.9) | | 26 (12.5) |
| 4 | 2 (1.8) | 0 | | 2 (1.0) |
| 5 | 0 | 1 (0.9) | | 1 (0.5) |
| Missing | 1 | 9 | | 10 |
| Speaks Dutch and/or English, *n (%)* |  |  | |  |
| Yes | 92 (85.2) | 84 (84.0) | | 176 (84.6) |
| No | 16 (14.8) | 16 (16.0) | | 32 (15.4) |
| Missing | 1 | 9 | | 10 |
| Total duration of stay in the Netherlands (in months), M (SD) (n missing) (range) | 69.1 (57.7) (45 missing) (3-240) | 77.7 (56.3) (61 missing) (3-216) | | 72.8 (57.0) (106 missing) (3-240) |
| With whom to the Netherlands, *n (%)* |  |  | |  |
| Alone | 52 (51.5) | 50 (49.0) | | 102 (50.2) |
| With partner | 26 (25.7) | 29 (28.4) | | 55 (27.1) |
| With partner + child(ren) | 11 (10.9) | 14 (13.7) | | 25 (12.3) |
| With friend(s) | 7 (6.9) | 7 (6.9) | | 14 (6.9) |
| With child(ren) | 3 (3.0) | 1 (1.0) | | 4 (2.0) |
| With parent(s) | 2 (2.0) | 1 (1.0) | | 3 (1.5) |
| Missing | 8 | 7 | | 15 |
| Mental health service consulted *before* leaving own country, *n (%)* |  |  | |  |
| Yes | 40 (39.6) | 37 (36.6) | | 77 (38.1) |
| No | 61 (60.4) | 64 (63.4) | | 125 (61.9) |
| Missing | 8 | 8 | | 16 |
| Mental health service consulted *after* leaving own country. *n (%)* |  |  | |  |
| Yes | 52 (51.5) | 52 (51.5) | | 104 (51.5) |
| No | 49 (48.5) | 49 (48.5) | | 98 (48.5) |
| Missing | 8 | 8 | | 16 |
| Mental health service consulted *before and/or* *after* leaving own country, *n (%)* |  |  | |  |
| Yes | 66 (65.3) | 65 (64.4) | | 131 (64.9) |
| No | 35 (34.7) | 36 (35.6) | | 71 (35.1) |
| Missing | 8 | 8 | | 16 |
| Educational level, *n (%)* |  |  | |  |
| Primary education | 2 (2.0) | 2 (2.0) | | 4 (2.0) |
| Secondary education | 42 (41.6) | 43 (42.6) | | 85 (42.1) |
| Professional education | 11 (10.9) | 8 (7.9) | | 19 (9.4) |
| University degree | 46 (45.5) | 48 (47.5) | | 94 (46.5) |
| Missing | 8 | 8 | | 16 |
| Work sector – country of origin, *n (%)* |  |  | |  |
| Agriculture | 6 (6.1) | 2 (2.0) | | 8 (4.1) |
| Arts | 2 (2.0) | 2 (2.0) | | 4 (2.0) |
| Cleaning | 1 (1.0) | 1 (1.0) | | 2 (1.0) |
| Commercial | 15 (15.3) | 16 (16.3) | | 31 (15.8) |
| Construction and installation | 5 (5.1) | 4 (4.1) | | 9 (4.6) |
| Education | 11 (11.2) | 9 (9.2) | | 20 (10.2) |
| Employment bursaries financed by Municipalities, NGOs or other systems | 1 (1.0) | 0 | | 1 (0.5) |
| Entertainment, sports and media | 1 (1.0) | 0 | | 1 (0.5) |
| Finance and economy | 5 (5.1) | 8 (8.2) | | 13 (6.6) |
| Healthcare | 8 (8.2) | 8 (8.2) | | 16 (8.2) |
| Industry (incl. mechanical and electrical sector) | 6 (6.1) | 11 (11.2) | | 17 (8.7) |
| Military, civil services, police | 1 (1.0) | 0 | | 1 (0.5) |
| Office and administrative support | 8 (8.2) | 8 (8.2) | | 16 (8.2) |
| Sales and services and hospitality (incl. restaurants and bars) | 10 (10.2) | 12 (12.2) | | 22 (11.2) |
| Student | 14 (14.3) | 8 (8.2) | | 22 (11.2) |
| Transport (goods and people) | 3 (3.1) | 6 (6.1) | | 9 (4.6) |
| Volunteering | 0 | 1 (1.0) | | 1 (0.5) |
| Not applicable (I was not working) | 1 (1.0) | 2 (2.0) | | 3 (1.5) |
| Missing | 11 | 11 | | 22 |
| Work sector – current, *n (%)* |  |  | |  |
| Administrative and support sectors |  |  | |  |
| Office and administrative support | 5 (5.2) | 6 (6.1) | | 11 (5.6) |
| Employment bursaries financed by Municipalities, NGOs or other systems | 1 (1.0) | 1 (1.0) | | 2 (1.0) |
| Volunteering | 1 (1.0) | 0 | | 1 (0.5) |
| Commercial sectors |  |  | |  |
| Commercial | 4 (4.2) | 10 (10.1) | | 14 (7.2) |
| Entertainment, sports and media | 3 (3.1) | 0 | | 3 (1.5) |
| Sales and services and hospitality (incl. restaurants and bars) | 3 (3.1) | 6 (6.1) | | 9 (4.6) |
| Finance and economy | 3 (3.1) | 1 (1.0) | | 4 (2.1) |
| Education, health care, and uniformed professions |  |  | |  |
| Education | 3 (3.1) | 7 (7.1) | | 10 (5.1) |
| Healthcare | 10 (10.4) | 9 (9.1) | | 19 (9.7) |
| Military, civil services, police | 1 (1.0) | 0 | | 1 (0.5) |
| Physical and industrial sectors |  |  | |  |
| Agriculture | 7 (7.3) | 4 (4.0) | | 11 (5.6) |
| Cleaning | 5 (5.2) | 4 (4.0) | | 9 (4.6) |
| Construction and installation | 6 (6.2) | 3 (3.0) | | 9 (4.6) |
| Industry (incl. mechanical and electrical sector) | 15 (15.6) | 15 (15.2) | | 30 (15.4) |
| Transport (goods and people) | 14 (14.6) | 19 (19.2) | | 33 (16.9) |
| Other |  |  | |  |
| Arts | 0 | 1 (1.0) | | 1 (0.5) |
| Student | 3 (3.1) | 2 (2.0) | | 5 (2.6) |
| Not applicable (I do have the right to work, but I do not currently work) | 12 (12.5) | 9 (9.1) | | 21 (10.8) |
| Not applicable (I do not have the right to work) | 0 | 2 (2.0) | | 2 (1.0) |
| Missing | 13 | 10 | | 23 |
| Hours work/week, M (SD) (n missing) (range)* | 35.3 (10.7) (0 missing) (2-70) | 35.9 (9.1) (1 missing) (0-52) | | 35.6 (9.9) (1 missing) (0-70) |
| Work schedule, *n (%)** |  |  | |  |
| Regular daytime hours | 54 (66.7) | 51 (61.4) | | 105 (64.0) |
| Shift work (changing hours) | 27 (33.3) | 32 (38.6) | | 59 (36.0) |
| Missing | 0 | 3 | | 3 |
| Income, *n (%)* |  |  | |  |
| Yes | 91 (91.9) | 91 (91.9) | | 182 (91.9) |
| No | 8 (8.1) | 8 (8.1) | | 16 (8.1) |
| Missing | 10 | 10 | | 20 |
| *Income, if yes:* source, *n (%)* |  |  | |  |
| Fixed contract directly with employer | 30 (44.1) | 35 (45.5) | | 65 (44.5) |
| Temporary contract directly with employer | 14 (20.6) | 12 (15.6) | | 26 (17.8) |
| Employment agency | 11 (16.2) | 15 (19.5) | | 26 (17.8) |
| Governmental aid / social grant | 5 (7.4) | 9 (11.7) | | 14 (9.6) |
| Self-employed | 6 (8.8) | 6 (7.8) | | 12 (8.2) |
| Old age pension and/or supplementary pension | 1 (1.5) | 0 | | 1 (0.7) |
| Volunteering reimbursement | 1 (1.5) | 0 | | 1 (0.7) |
| Missing | 23 | 14 | | 37 |
| *Income source, if employment agency:* contract type, *n (%)* |  |  | |  |
| Phase A | 8 (72.7) | 6 (42.9) | | 14 (56.0) |
| Phase B | 1 (9.1) | 5 (35.7) | | 6 (24.0) |
| Phase C | 1 (9.1) | 3 (21.4) | | 4 (16.0) |
| I don’t know | 1 (9.1) | 0 | | 1 (4.0) |
| Missing | 0 | 1 | | 1 |
| Relationship status, *n (%)* |  |  | |  |
| In a steady relationship or married | 73 (67.0) | 67 (61.5) | | 140 (64.2) |
| Single | 28 (25.7) | 30 (27.5) | | 58 (26.6) |
| Divorced or separated | 7 (6.4) | 12 (11.0) | | 19 (8.7) |
| Widowed | 1 (0.9) | 0 | | 1 (0.5) |
| Number of children, *n (%)* |  |  | |  |
| 0 | 61 (56.0) | 60 (55.6) | | 121 (55.8) |
| 1 | 23 (21.1) | 16 (14.8) | | 39 (18.0) |
| 2 | 17 (15.6) | 22 (20.4) | | 39 (18.0) |
| 3 | 6 (5.5) | 9 (8.3) | | 15 (6.9) |
| 4 | 2 (1.8) | 1 (0.9) | | 3 (1.4) |
| Missing | 0 | 1 | | 1 |
| Country of residence, *n (%)* |  |  | |  |
| I live permanently in the Netherlands | 102 (93.6) | 102 (94.4) | | 204 (94.0) |
| I live alternatively in the Netherlands and in my country of birth | 7 (6.4) | 5 (4.6) | | 12 (5.5) |
| I live alternatively in the Netherlands and in another country/countries | 0 | 1 (0.9) | | 1 (0.5) |
| Missing | 0 | 1 | | 1 |
| Close relatives abroad, *n (%)* |  |  | |  |
| Yes | 92 (85.2) | 93 (86.9) | | 185 (86.0) |
| No | 16 (14.8) | 14 (13.1) | | 30 (14.0) |
| Missing | 1 | 2 | | 3 |
| Living situation, *n (%)* |  |  | |  |
| Living alone | 11 (10.5) | 15 (14.0) | | 26 (12.3) |
| Living alone with child(ren) | 6 (5.7) | 9 (8.4) | | 15 (7.1) |
| Living with partner | 37 (35.2) | 37 (34.6) | | 74 (34.9) |
| Living with partner and child(ren) | 27 (25.7) | 23 (21.5) | | 50 (23.6) |
| Living with parents | 0 | 2 (1.9) | | 2 (0.9) |
| Living with other relatives | 0 | 1 (0.9) | | 1 (0.5) |
| Living with ex-partner | 4 (3.8) | 1 (0.9) | | 5 (2.4) |
| Living with friend(s) | 3 (2.9) | 3 (2.8) | | 6 (2.8) |
| Living with roommate(s) | 17 (16.2) | 16 (15.0) | | 33 (15.6) |
| Missing | 4 | 2 | | 6 |
| Household size, *n (%)* |  |  | |  |
| 1 | 13 (12.4) | 22 (21.0) | | 35 (16.7) |
| 2 | 42 (40.0) | 37 (35.2) | | 79 (37.6) |
| 3 | 26 (24.8) | 20 (19.0) | | 46 (21.9) |
| 4 | 15 (14.3) | 15 (14.3) | | 30 (14.3) |
| 5 | 4 (3.8) | 9 (8.6) | | 13 (6.2) |
| 6 or more | 5 (4.8) | 2 (1.9) | | 7 (3.3) |
| Missing | 4 | 4 | | 8 |
| Living accommodation, *n (%)* |  |  | |  |
| Rented apartment or house for myself (and my family) | 55 (53.4) | 56 (53.3) | | 111 (53.4) |
| Bought apartment or house for myself (and my family) | 29 (28.2) | 26 (24.8) | | 55 (26.4) |
| Shared housing | 15 (14.6) | 18 (17.1) | | 33 (15.9) |
| Accommodation center | 1 (1.0) | 2 (1.9) | | 3 (1.4) |
| Other (e.g. camping, worker’s hotel) | 2 (1.9) | 2 (1.9) | | 4 (1.9) |
| I don't have accommodation (e.g. sleeping at a friends' house, in a homeless shelter, or on the street) | 1 (1.0) | 1 (1.0) | | 2 (1.0) |
| Missing | 6 | 4 | | 10 |
| *Living accommodation, if shared housing:* shared bedroom, *n (%)*† |  |  | |  |
| No | 8 (72.7) | 12 (100) | | 20 (87.0) |
| Yes, with partner and/or child(ren) | 1 (9.1) | 0 | | 1 (4.3) |
| Yes, with others | 2 (18.2) | 0 | | 2 (8.7) |
| Anxiety/depression symptoms (PHQ-ADS score, 0-48), *M (SD)* (range) | 23.89 (9.16) (8-48) | 25.49 (10.44) (5-46) | | 24.69 (9.83) (5-48) |
| Anxiety symptoms (GAD-7 score, 0-21), *M (SD)* (range) | 10.72 (4.85) (3-21) | 11.44 (5.44) (1-21) | | 11.08 (5.15) (1-21) |
| Probable anxiety disorder (GAD-7 ≥10), *n (%)* | 63 (57.8) | 67 (61.5) | | 130 (59.6) |
| Depression symptoms (PHQ-9 score, 0-27), *M (SD)* (range) | 13.17 (5.09) (5-27) | 14.05 (5.65) (4-26) | | 13.61 (5.38) (4-27) |
| Probable depressive disorder (PHQ-9 ≥10), *n (%)* | 79 (72.5) | 80 (73.4) | | 159 (72.9) |
| Post-traumatic stress symptoms (PCL-5 score, 0–32), *M (SD)* (range) | 17.54 (6.88) (3-31) | 19.23 (6.79) (2-32) (2 missing) | | 18.38 (6.87) (2-32) (2 missing) |
| Probable PTSD (PCL-5 ≥19), *n (%)* | 56 (51.4) | 63 (58.9) | | 119 (55.1) |
| Traumatic experiences (brief BTQ score, 0-10), *M (SD)* (range) | 1.82 (1.88) (0-8) | 2.39 (2.48) (0-10) | | 2.10 (2.21) (0-10) |
| At least 1 traumatic experience (BTQ), *n (%)* | 79 (72.5) | 80 (73.4) | | 159 (72.9) |
| Ever infected with COVID-19, *n (%)* |  |  | |  |
| Yes, confirmed by a formal COVID-19 test | 57 (52.3) | 67 (62.0) | | 124 (57.1) |
| Most likely, but no formal test was done | 24 (22.0) | 14 (13.0) | | 38 (17.5) |
| No | 28 (25.7) | 27 (25.0) | | 55 (25.3) |
| Missing | 0 | 1 | | 1 |
| *Ever infection, if not ‘No’:* Severity of COVID-19 symptoms, *n (%)* |  |  | |  |
| No or mild symptoms (not hospitalized) | 16 (20.0) | 16 (19.8) | | 32 (19.9) |
| Moderate symptoms (not hospitalized) | 63 (78.8) | 61 (75.3) | | 124 (77.0) |
| Severe symptoms (hospitalized) | 1 (1.2) | 4 (4.9) | | 5 (3.1) |
| Missing | 1 | 0 | | 1 |
| Worry about own infection with COVID-19, *n (%)* |  |  | |  |
| Not at all | 54 (49.5) | 51 (46.8) | | 105 (48.2) |
| Slightly | 41 (37.6) | 41 (37.6) | | 82 (37.6) |
| Considerably | 13 (11.9) | 13 (11.9) | | 26 (11.9) |
| Extremely | 1 (0.9) | 4 (3.7) | | 5 (2.3) |
| Worry about infecting others with COVID-19, *n (%)* |  |  | |  |
| Not at all | 29 (26.9) | 31 (28.7) | | 60 (27.8) |
| Slightly | 50 (46.3) | 38 (35.2) | | 88 (40.7) |
| Considerably | 16 (14.8) | 27 (25.0) | | 43 (19.9) |
| Extremely | 13 (12.0) | 12 (11.1) | | 25 (11.6) |
| Missing | 1 | 1 | | 2 |
| Adherence to COVID-19 measures, *n (%)* |  |  | |  |
| Not at all | 7 (6.5) | 8 (7.5) | | 15 (7.0) |
| Most of the time | 56 (51.9) | 49 (45.8) | | 105 (48.8) |
| All of the time | 45 (41.7) | 50 (46.7) | | 95 (44.2) |
| Missing | 1 | 2 | | 3 |
| COVID-19 vaccination status, *n (%)* |  |  | |  |
| Not vaccinated | 26 (24.3) | 26 (24.3) | | 52 (24.3) |
| Vaccinated | 81 (75.7) | 81 (75.7) | | 162 (75.7) |
| Missing | 2 | 2 | | 4 |
| *If unvaccinated:* Willingness to vaccinate, *n (%)* |  |  | |  |
| No | 26 (100) | 26 (100) | | 52 (100) |
| *Quarantine experience*, *n (%)* |  |  | |  |
| Yes | 60 (56.6) | 63 (58.9) | | 123 (57.7) |
| No | 46 (43.4) | 44 (41.1) | | 90 (42.3) |
| Missing | 3 | 2 | | 5 |
| Close contact: mild/moderate COVID-19 infection, *n (%)* |  |  | |  |
| Yes | 86 (79.6) | 82 (76.6) | | 168 (78.1) |
| No | 22 (20.4) | 25 (23.4) | | 47 (21.9) |
| Missing | 1 | 2 | | 3 |
| Close contact: severe COVID-19 infection, *n (%)* |  |  | |  |
| Yes | 29 (27.1) | 14 (13.3) | | 43 (20.3) |
| No | 78 (72.9) | 91 (86.7) | | 169 (79.7) |
| Missing | 2 | 4 | | 6 |
| Close contact: death due to COVID-19, *n (%)* |  |  | |  |
| Yes | 18 (16.8) | 11 (10.5) | | 29 (13.7) |
| No | 89 (83.2) | 94 (89.5) | | 183 (86.3) |
| Missing | 2 | 4 | | 6 |
| Government COVID-19 related financial support, *n (%)* |  |  | |  |
| Yes | 5 (4.7) | 5 (4.8) | | 10 (4.7) |
| No | 102 (95.3) | 100 (95.2) | | 202 (95.3) |
| Missing | 2 | 4 | | 6 |
| Income loss due to COVID-19, *n (%)* |  |  | |  |
| Yes | 27 (25.5) | 21 (20.4) | | 48 (23.0) |
| No | 68 (64.2) | 72 (69.9) | | 140 (67.0) |
| Don't know | 11 (10.4) | 10 (9.7) | | 21 (10.0) |
| Missing | 3 | 6 | | 9 |
| Difficulty accessing basic needs due to COVID-19, *n (%)* |  |  | |  |
| Yes | 14 (13.5) | 18 (18.2) | | 32 (15.8) |
| No | 84 (80.8) | 76 (76.8) | | 160 (78.8) |
| Don't know | 6 (5.8) | 5 (5.1) | | 11 (5.4) |
| Missing | 5 | 10 | | 15 |
| Increased responsibilities due to COVID-19, *n (%)* |  |  | |  |
| Yes | 13 (12.5) | 21 (21.0) | | 34 (16.7) |
| No | 86 (82.7) | 72 (72.0) | | 158 (77.5) |
| Don't know | 5 (4.8) | 7 (7.0) | | 12 (5.9) |
| Missing | 5 | 9 | | 14 |
| Negative psychological impact of COVID-19, *n (%)* |  |  | |  |
| Yes | 57 (54.3) | 62 (62.6) | | 119 (58.3) |
| No | 23 (21.9) | 11 (11.1) | | 34 (16.7) |
| Don't know | 25 (23.8) | 26 (26.3) | | 51 (25.0) |
| Missing | 4 | 10 | | 14 |
| Experienced stigma/ discrimination due to COVID-19, *n (%)* |  |  | |  |
| Yes | 26 (24.8) | 23 (23.5) | | 49 (24.1) |
| No | 72 (68.6) | 69 (70.4) | | 141 (69.5) |
| Don't know | 7 (6.7) | 6 (6.1) | | 13 (6.4) |
| Missing | 4 | 11 | | 15 |
| Unable to access essential service due to COVID-19, *n (%)* |  |  | |  |
| Yes | 21 (20.2) | 12 (12.2) | | 33 (16.3) |
| No | 81 (77.9) | 81 (82.7) | | 162 (80.2) |
| Don't know | 2 (1.9) | 5 (5.1) | | 7 (3.5) |
| Missing | 5 | 11 | | 16 |
| Administrative delays due to COVID-19, *n (%)* |  |  | |  |
| Yes | 45 (43.3) | 46 (46.9) | | 91 (45.0) |
| No | 47 (45.2) | 41 (41.8) | | 88 (43.6) |
| Don't know | 12 (11.5) | 11 (11.2) | | 23 (11.4) |
| Missing | 5 | 11 | | 16 |

*Note.* * Questions about weekly hours and shift work were only presented to participants who reported current employment (*n* = 167; 81 intervention, 86 control), excluding “Student,” “Not applicable (I do have the right to work, but I do not currently work),” “Not applicable (I do not have the right to work),” and missing data.
† This question was only presented to participants who initially selected “Shared housing” (*n* = 23). After regrouping open-text responses, 10 additional participants were categorized as living in shared housing but did not receive this follow-up question.
Abbreviations: M = mean; SD = standard deviation; PHQ-ADS = Patient Health Questionnaire-Anxiety and Depression Scale; PHQ-9 = Patient Health Questionnaire-9; GAD-7 = Generalized Anxiety Disorder-7; PCL-5 = PTSD Checklist for DSM-5; BTQ = Brief Trauma Questionnaire.
