# Supplementary material for: Effectiveness of a scalable, remotely delivered stepped-care intervention for psychological distress among Polish migrant workers in the Netherlands: The RESPOND randomised controlled trial
Source: PLoS Med. 2026 Aug 17;23(8):e1005197. doi: 10.1371/journal.pmed.1005197 (PMC13480649; doi:10.1371/journal.pmed.1005197)
Supplement: Supplementary file 6 [file pmed.1005197.s004.docx]

**S2 Table**
*Primary and Secondary Outcome Summary Statistics, Estimated Marginal Means, and Effect Sizes per Timepoint (Intention-to-Treat, Covariate-Adjusted Linear Mixed Model)*

|  |  | **Intervention** | | | **Control** | | |  |  |  |
| --- | --- | --- | --- | --- | --- | --- | --- | --- | --- | --- |
| **Outcome** | **Timepoint** | ***N*** | ***M (SD)*** | **EMM  (95% CI)** | ***N*** | ***M (SD)*** | **EMM  (95% CI)** | **Difference (95% CI)** | ***p*-value** | **Cohen’s *d*** |
| PHQ-ADS | *t*_1_ (baseline, week 1) | 109 | 23.9 (9.2) |  | 109 | 25.5 (10.4) |  |  |  |  |
|  | *t*_2_ (post-DWM, week 7) | 95 | 17.5 (10.2) | 19.1  (14.2, 24.0) | 98 | 24.7 (11.0) | 24.2  (19.4, 29.0) | -5.07  (-7.57, -2.57) | < 0.001 | -0.48 |
|  | *t*_3_ (post-PM+, week 13) | 83 | 15.1 (9.8) | 16.7  (11.8, 21.7) | 94 | 21.1 (12.2) | 20.7  (15.9, 25.6) | -4.00  (-6.64, -1.36) | 0.003 | -0.36 |
|  | *t*_4_ (follow-up, week 21) | 94 | 14.4 (10.0) | 14.8  (9.9, 19.7) | 101 | 22.1 (12.6) | 21.8  (17.0, 26.6) | -7.04  (-9.55, -4.53) | < 0.001 | -0.62 |
| PHQ-9 | *t*_1_ (baseline, week 1) | 109 | 13.2 (5.1) |  | 109 | 14.0 (5.6) |  |  |  |  |
|  | *t*_2_ (post-DWM, week 7) | 95 | 9.7 (5.5) | 10.1  (7.3, 12.9) | 98 | 13.8 (6.3) | 13.2  (10.4, 15.9) | -3.08  (-4.5, -1.65) | < 0.001 | -0.52 |
|  | *t*_3_ (post-PM+, week 13) | 84 | 8.5 (5.6) | 8.7  (5.9, 11.5) | 94 | 11.7 (6.9) | 11.0  (8.2, 13.8) | -2.29  (-3.79, -0.79) | 0.003 | -0.36 |
|  | *t*_4_ (follow-up, week 21) | 94 | 8.1 (5.6) | 7.8  (5.0, 10.6) | 101 | 12.1 (7) | 11.6  (8.8, 14.3) | -3.77  (-5.2, -2.35) | < 0.001 | -0.59 |
| GAD-7 | *t*_1_ (baseline, week 1) | 109 | 10.7 (4.9) |  | 109 | 11.4 (5.4) |  |  |  |  |
|  | *t*_2_ (post-DWM, week 7) | 95 | 7.8 (5.2) | 8.9  (6.4, 11.4) | 98 | 10.9 (5.4) | 10.9  (8.5, 13.4) | -2.04  (-3.36, -0.72) | 0.002 | -0.38 |
|  | *t*_3_ (post-PM+, week 13) | 83 | 6.8 (5) | 8.0  (5.4, 10.5) | 94 | 9.3 (6.1) | 9.6  (7.1, 12.1) | -1.65  (-3.05, -0.26) | 0.020 | -0.30 |
|  | *t*_4_ (follow-up, week 21) | 94 | 6.2 (4.9) | 6.9  (4.3, 9.4) | 101 | 10 (6.2) | 10.2  (7.7, 12.6) | -3.32  (-4.64, -2.00) | < 0.001 | -0.59 |
| PCL-5 | *t*_1_ (baseline, week 1) | 109 | 17.5 (6.9) |  | 107 | 19.2 (6.8) |  |  |  |  |
|  | *t*_2_ (post-DWM, week 7) | 94 | 13.7 (7.7) | 14.4 (10.7, 18.2) | 98 | 17.5 (7.7) | 16.6 (12.9, 20.4) | -2.18 (-4.08, -0.28) | 0.024 | -0.29 |
|  | *t*_3_ (post-PM+, week 13) | 81 | 11.8 (7.1) | 13 (9.2, 16.8) | 89 | 16.3 (8.7) | 15.3 (11.6, 19.1) | -2.31 (-4.35, -0.28) | 0.026 | -0.29 |
|  | *t*_4_ (follow-up, week 21) | 94 | 10.9 (7.9) | 11.6 (7.9, 15.4) | 99 | 13.9 (8.9) | 13.4 (9.7, 17.1) | -1.79 (-3.69, 0.11) | 0.065 | -0.21 |

*Note.* Observed descriptive values, presented as N and M (SD), are unadjusted. Model-based estimates, including EMMs, between-group differences, p-values, and Cohen’s d, are adjusted for the respective baseline outcome score, gender, age, education level, relationship status, work hours per week (based on work sector), trauma history (any vs none), and COVID-19 infection history. Covariates were derived from baseline questionnaire data and, where required, recoded or grouped for analysis.
Abbreviations: M = mean; SD = standard deviation; EMM = estimated marginal mean; CI = confidence interval; PHQ-ADS = Patient Health Questionnaire-Anxiety and Depression Scale; PHQ-9 = Patient Health Questionnaire-9; GAD-7 = Generalized Anxiety Disorder-7; PCL-5 = PTSD Checklist for DSM-5; DWM = Doing What Matters in Times of Stress; PM+ = Problem Management Plus.
